# Variations in phenological, physiological, plant architectural and yield-related traits, their associations with grain yield and genetic basis

**DOI:** 10.1093/aob/mcad003

**Published:** 2023-01-19

**Authors:** Yibo Li, Fulu Tao, Yuanfeng Hao, Jingyang Tong, Yonggui Xiao, Zhonghu He, Matthew Reynolds

**Affiliations:** Key Laboratory of Land Surface Pattern and Simulation, Institute of Geographic Sciences and Natural Resources Research, CAS, Beijing 100101, China; University of Chinese Academy of Sciences, Beijing 100049, China; Key Laboratory of Land Surface Pattern and Simulation, Institute of Geographic Sciences and Natural Resources Research, CAS, Beijing 100101, China; University of Chinese Academy of Sciences, Beijing 100049, China; Natural Resources Institute Finland (Luke), Helsinki, Finland; Institute of Crop Sciences, Chinese Academy of Agricultural Sciences, Beijing 100081, China; Institute of Crop Sciences, Chinese Academy of Agricultural Sciences, Beijing 100081, China; Institute of Crop Sciences, Chinese Academy of Agricultural Sciences, Beijing 100081, China; Institute of Crop Sciences, Chinese Academy of Agricultural Sciences, Beijing 100081, China; International Maize and Wheat Improvement Center (CIMMYT), Texcoco, Mexico

**Keywords:** Genetic variation, ideotypes, photosynthetic traits, *Triticum aestivum*, yield potential

## Abstract

**Background and Aims:**

Physiological and morphological traits play essential roles in wheat (*Triticum aestivum*) growth and development. In particular, photosynthesis is a limitation to yield. Increasing photosynthesis in wheat has been identified as an important strategy to increase yield. However, the genotypic variations and the genomic regions governing morphological, architectural and photosynthesis traits remain unexplored.

**Methods:**

Here, we conducted a large-scale investigation of the phenological, physiological, plant architectural and yield-related traits, involving 32 traits for 166 wheat lines during 2018–2020 in four environments, and performed a genome-wide association study with wheat 90K and 660K single nucleotide polymorphism (SNP) arrays.

**Key Results:**

These traits exhibited considerable genotypic variations in the wheat diversity panel. Higher yield was associated with higher net photosynthetic rate (*r* = 0.41, *P* < 0.01), thousand-grain weight (*r* = 0.36, *P* < 0.01) and truncated and lanceolate shape, but shorter plant height (*r* = −0.63, *P* < 0.01), flag leaf angle (*r* = −0.49, *P* < 0.01) and spike number per square metre (*r* = −0.22, *P* < 0.01). Genome-wide association mapping discovered 1236 significant stable loci detected in the four environments among the 32 traits using SNP markers. Trait values have a cumulative effect as the number of the favourable alleles increases, and significant progress has been made in determining phenotypic values and favourable alleles over the years. Eleven elite cultivars and 14 traits associated with grain yield per plot (GY) were identified as potential parental lines and as target traits to develop high-yielding cultivars.

**Conclusions:**

This study provides new insights into the phenotypic and genetic elucidation of physiological and morphological traits in wheat and their associations with GY, paving the way for discovering their underlying gene control and for developing enhanced ideotypes in wheat breeding.

## INTRODUCTION

Wheat (*Triticum aestivum*) accounts for about 20 % of humans’ daily protein and calorie intake globally (FAO, 2019). The world’s population is expected to reach 9.6 billion by 2050. However, the global growth rate of wheat productivity is only 1.1 % per annum (Dixon *et al.*, 2009), and has even stagnated in some regions (Ray *et al.*, 2019). An annual gain of only about 2 % in grain yield may meet the projected global requirement for wheat (Li *et al.*, 2019a). Therefore, increasing annual yield gain is critical for food security (Lopes *et al.*, 2012; Abbai *et al.*, 2020; Xiao *et al.*, 2022). Today, the optimization of agronomic management and sustainable intensification is increasingly accompanied by genomic and phenomics technologies to further improve yield productivity (Godfray *et al.*, 2010; Fu *et al.*, 2020; Pang *et al.*, 2020; Welcker *et al.*, 2022).

A quantitative understanding of the mechanisms influencing yield gains is important for major food crops (Rizzo *et al.*, 2022). Since the green revolution between 1950 and the late 1960s, improved grain yield has mostly been due to an increase in harvest index, now close to the theoretical maximum. Another important strategy to increase crop yield further and solve the food crisis is improving the photosynthetic efficiency of crops. This strategy represents the core of a possible second green revolution (Xu & Shen, 2002; Driever *et al.*, 2014). Improvement in any of the canopy photosynthesis contributors reflects a potential increase in yield and biomass production (Takai *et al.*, 2013). As the primary determinant of plant productivity, photosynthesis still has the potential to improve radiation use efficiency (RUE) (Long *et al.*, 2006; Zhu *et al.*, 2010; Parry *et al.*, 2011; Raines, 2011; Li *et al.*, 2022a). Studies have shown a positive relationship between photosynthesis, biomass and yield (Fischer *et al.*, 1998; Kruger & Volin, 2006). Theoretically, photosynthesis could be improved by increasing the photosynthetic rate per unit leaf area and optimizing light interception and utilization by modifying architecture and photosynthetic duration (Driever *et al.*, 2014). By unravelling the genetics of complicated characteristics and having a better understanding of the molecular processes of genes that support desirable features, the novel genomic areas or candidate genes discovered could be utilized to enhance crops (Cui *et al.*, 2011; Li *et al.*, 2022b). Many genes and hotspot genomic regions influencing target traits for crop improvement have been identified thanks to recent improvements in DNA sequencing technologies (Azadi *et al.*, 2015). By matching crop genotypes to target environments, adopting agroecological genetics and genomics perspectives may maximize communal yield (Abbai *et al.*, 2020). Studies of photosynthesis using molecular modification techniques, such as improving ribulose-1,5 bisphosphate carboxylase/oxygenase activity, faster regeneration of ribulose-1,5-bisphosphate, and introducing carbon-concentrating mechanisms, have proposed increases in the photosynthetic rate per unit leaf area (Parry *et al.*, 2011; Bailey *et al.*, 2019). Thanks to high-throughput phenotyping, we can now map the genetic regions (and genes) that control variation in physiological traits, such as photosynthesis, that would otherwise be impossible to score in large panels under field conditions (Flood *et al.*, 2011). Natural variations in the rate of photosynthesis have been reported in previous studies (Hubbart *et al.*, 2007; Kanemura *et al.*, 2007; Ohsumi *et al.*, 2007; Gu *et al.*, 2012), indicating that photosynthesis improvement is certainly recognized as an important target and is expected to be possible, even though genetic analysis for photosynthesis lags behing that for sink size (Takai *et al.*, 2013). Therefore, identifying genomic regions associated with high RUE and photosynthetic capacity will help in breeding high-yielding cultivars (Driever *et al.*, 2014).

RUE optimization can be achieved by improving light conversion efficiency into harvestable grains and by increasing the interception of photosynthetically active radiation (PAR) (Beer *et al.*, 2010; Li *et al.*, 2021). Flag leaves, as the ‘functional leaves’ in wheat production, are the primary photosynthetic organ contributing 45–58 % of photosynthetic performance during the grain-filling stage (Sharma *et al.*, 2003; Khaliq & Irshad, 2008). Flag leaf angle (FLANG) determines the amount of incident light received for photosynthesis by leaves. Breeders have utilized this trait to optimize plant architecture (Morinaka *et al.*, 2006). Studies have also shown a positive correlation in cereals of flag leaf width (FLW), flag leaf length (FLL) and flag leaf area (FLA) with thousand-grain weight (TGW) (Wang *et al.*, 2011b). Thus, a comprehensive understanding of flag leaf physiological and morphological traits will provide new insights related to RUE and yield. Identifying quantitative trait loci (QTLs) controlling flag leaf traits is an essential way to enhance marker-assisted selection (MAS) for wheat yield improvement through QTL pyramiding (Yang *et al.*, 2016).

A crop ideotype consists of several morphological and physiological traits that contribute to enhanced yield or better performance than in the current crop cultivars. Recognizing crop ideotypic traits has become a priority for breeding high-yielding cultivars and for designing ideotypes (Tao *et al.*, 2017a; Bodner *et al.*, 2018). Some agronomic traits, such as crop architecture, phenological date, spike and grain-related morphological characteristics, also play crucial roles in yield formation (Jaiswal *et al.*, 2016; Sun *et al.*, 2017). Thus, understanding the natural variation among traits may promote new variation for desirable traits, in which case additional genetic modifications may be targeted to further improve crop yields. The introduction of novel adaptive alleles into genetic germplasms may improve grain production of old or newly produced wheat cultivars to further balance global supply and demand (Rahimi *et al.*, 2019). MAS, relying on identifying agronomic trait loci and the characterization of their genetic architecture, has been applied for this purpose. Moreover, genome-wide association studies (GWAS) have become popular as a method to identify marker–trait correlations (Wang *et al.*, 2014; Li *et al.*, 2018a). High-resolution GWAS mapping has been applied to crops to investigate the complex genetic architecture of polygenic traits (Cavanagh *et al.*, 2013; Fu *et al.*, 2020). Complex polygenic traits in wheat, such as yield and bread-making quality, have been improved through QTLs/genes identified in association mapping studies (Crossa *et al.*, 2007; Arif *et al.*, 2012). However, few studies have comprehensively investigated the natural variation in phenological and physiological processes, plant architecture, yield-related traits, as well as their genetic basis.

Therefore, the objectives of this study were to (1) investigate the phenotypic and genetic relationships among physiological processes and yield, (2) identify stable loci for grain yield per plot (GY) and physiological process traits using GWAS based on high-density single nucleotide polymorphism (SNP) markers, (3) detect the available loci of traits in breeding for high yield and photosynthesis, and (4) characterize the 166 elite wheat cultivars and identify potential parent sources to develop new, high-yielding cultivars.

## MATERIALS AND METHODS

### Plant materials and field trials

This study’s association panel comprised 166 representative elite wheat (*Triticum aestivum* L.) cultivars chosen from more than 400 cultivars (Supplementary Data Table S1), including 141 from the Yellow and Huai River Valley Zone (YHV), the major wheat-producing region of China (15.3 million hectares, accounting for ~65 % of national wheat production), and 22 from five other countries (Liu *et al.*, 2017; Zhu *et al.*, 2019; Fu *et al.*, 2020). These cultivars represent the genetic diversity of the YHV. These cultivars were divided into five groups as follows: nine released during 1947–1979 (P1), 13 in the 1980s (P2), 36 in the 1990s (P3), 59 in the 2000s (P4) and 13 in the 2010s (P5). The association panel was grown in four environments and years, including Zhoukou (33°37ʹN, 114°38ʹE; ZK19) and Xinxiang (35°18ʹN, 113°51ʹE; XX19) during 2018–2019, and Luohe (33°36ʹN, 113°58ʹE; LH20) and Xinxiang (35°18ʹN, 113°51ʹE; XX20) during 2019–2020. Field trials were conducted in a randomized block design with two replications at all locations under well-watered conditions. Irrigation was applied at least three times between four critical stages (sowing at GS21, tillering at GS27, booting at GS31 and flowering at GS73) using diffuse irrigation. The area of each plot was 4.2 m^2^ (3 m in length, 1.4 m in width). Sowing density was 270 seeds m^−2^ with a row spacing of 20 cm. Agricultural management, including fertilizer application, irrigation, weed management, insect pest control throughout the growing season and harvesting for all environments, were kept the same in all four environments.

### Phenotypic trait evaluation

At the flowering stage, the main tillers of five representative plants from each plot were used for phenotypic evaluation of the flag leaf. The distance from the tip to the base was regarded as flag leaf length (FLL, cm), and the widest section of the flag leaf was flag leaf width (FLW, cm). Flag leaf area (FLA, cm^2^) was calculated using the equation 0.77 × FLL × FLW (Li *et al.*, 2021). Flag leaf angle (FLANG, °) was measured as the angle between the flag leaf midrib and the stem below the spike, with a smaller leaf angle indicating more upright leaves. Flag leaf biomass (FLB, g) was measured after drying for 72 h at 75 °C in an oven to a constant weight. Flag specific leaf area (FSLA, cm^2^ g^−1^) was defined as leaf area ratio to leaf biomass. Chlorophyll content SPAD meter readings (Minolta Camera Co., Osaka, Japan) were used to assess the relative chlorophyll contents at the flowering stage. SPAD readings from five plants per plot were averaged at each flag leaf’s top, middle and bottom.

Leaf gas exchanges and chlorophyll fluorescence parameters of flag leaves, including light-saturated net photosynthetic rate (Pn, μmol CO_2_ m^−2^ s^−1^), stomatal conductance (Gs, mol H_2_O m^−2^ s^−1^), transpiration rate (Tr, mmol H_2_O m^−2^ s^−1^), intercellular CO_2_ concentration (Ci, µmol CO_2_ m^−2^ s^−1^) and the maximum quantum yield of photosystem II (PSII) photochemistry (Fvʹ/Fmʹ), were measured during clear sky mornings (0900–1100 h) using an infrared gas analyser (LI-6400XT; Li-Cor, Lincoln, NE, USA) with a fluorescence leaf chamber (LI-6400-40; Li-Cor). The saturating photosynthetic photon flux density was set to 1200 µmol m^−2^ s^−1^. Water use efficiency (WUE) was calculated as the ratio of Pn to Tr, and intrinsic water use efficiency (iWUE) as the ratio of Pn to Gs.

The gas exchange parameters, such as photosynthetic rate (Pn) and other related traits (Gs), are sensitive to microclimate fluctuations, such as vapour pressure deficit (VPD) or leaf temperature (Cowan, 1977; Buckley *et al.*, 2002; Supplementary Data Fig. S1). The modified methodology for microclimatic differences utilizes a statistical covariant model using the environmental variable as a quantitative co-regressor (Gu *et al.*, 2012). The photosynthetic trait value of the *i*th genetic line was expressed in a statistical covariant model using the environmental characteristics as a quantitative co-regressor:


$$\begin{array}{l} \mu_{ijk}=\mu +G_{i}+E_{j}+\;{\left(GE\right)}_{ij}+B_{k}+bx_{ijk}+e_{ijk} \end{array}$$ (1)

where *u* is the general mean. *G*_*i*_ is the genetic effect of the *i*th genotype, *E*_*ij*_ is the treatment effect, which represents the four environments, (*GE*)_*ij*_ is the genetype × treatment interaction, *B*_*k*_ is the block effect, *b* is the effect of the environmental variable, *x*_*ijk*_ is the environmental variable during measurement and *e*_*ijk*_ is the residual effect. This method allows the observed photosynthetic traits to be statistically adjusted to the same value (such as the average) of the climatic variable (*T*_leaf_ and VPD) that changed during the field measurements. Here, we used VPD as the most crucial environmental component in this study (Supplementary Data Fig. S1).

Plant height (PH, cm) was measured at anthesis as the distance from the top of the canopy to the soil; height was measured for five plants in each plot and averaged. Leaf area index (LAI) was recorded using a Sunscan Canopy Analysis System (Delta T Devices Ltd, Cambridge, UK) with 64 photodiodes. Ten spikes were randomly harvested in each plot at anthesis, 10 d after flowering and 20 d after flowering to calculate the grain-filling rate (GFR). The stay-green trait, an indicator of maintaining green character, was determined as the percentage of decline in SPAD value at the grain-filling stage compared with the flowering stage. Days to anthesis and maturity from sowing were noted when more than 50 % of the plants of each plot displayed anthesis and maturity at Zadoks GS65 and GS92 stages, respectively (Zadoks, 1974). Moreover, the thermal time from sowing to flowering stage (TTF) and from sowing to maturity stage (TTM) were calculated. The shape of the wheat spike was acuminate, fusiform, linear, truncated and lanceolate (Bonnett, 1966; Backhaus *et al.*, 2022), assigned values of 1, 2, 3, 4 and 5, respectively. The larger the spike shape, the more it tends to be truncated and lanceolate in shape. The smaller the spike shape, the more likely it is to be fusiform and acuminate in shape. A detailed image and description are provided in Supplementary Data Fig. S2.

The leaves, spikes and stems were clipped and weighed to determine the fresh weight at the anthesis stage. They were dried in an oven for 72 h at 75 °C to a constant weight. Leaf water content (LWC), spike water content (SPWC) and stem water content (STWC) were calculated as the ratio of the corresponding water content of dry biomass. Leaf dry weights (LDWS), spike dry weights (SPDWS), stem dry weights (STDWS) and total dry weights (TDW) were measured after drying. After harvest, the kernel number per spike (KN, kernels per spike) and the spike number per square meter (spikes m^–2^) were measured. After air-drying, thousand-grain weight (TGW) and grain yield per plot (GY) were calculated.

### Phenotypic data analysis

Analysis of variance (ANOVA) was performed for the phenotypic data using SAS software (Version 9.2; SAS Institute Inc., Cary, NC, USA). Broad-sense heritability was estimated using the formula:


$$\begin{array}{l} h^{2}=\sigma_{G}^{2}/(\sigma_{G}^{2}+\sigma_{GE}^{2}/e\;+\sigma_{\varepsilon }^{2}/(re)) \end{array}$$ (2)

where *e* and *r* are the number of environments and replications per environment, respectively. Mean squares were used to estimate the variance components for genotypes ($\sigma_{G}^{2}$), genotype × environment interaction ($\sigma_{GE}^{2}$) and residual error ($\sigma_{\varepsilon }^{2}$), respectively. Best linear unbiased estimations (BLUE) for phenotypic data across the four environments were extracted by implementing the ANOVA function in the QTL IciMapping software (Version 4.1). Correlation analyses and *t*-tests were performed using SAS software.

The random forest model (*randomForest* package in R) was used to assess trait contributions to GY and TGW. The relative effect of the parameter estimates for each predictor was compared with the impact of all parameter estimates in the model to evaluate the relative importance of traits. Self-organizing maps (SOMs, *kohonen* package in R) were then used to identify the traits suitable for GY and evaluate the favourable traits of 166 elite cultivars. SOMs represent a non-linear multivariate statistical method helpful in visualizing multidimensional data. This method allows the transformation of high-dimensional data to low-dimensional data, preserving the input data’s main characteristics. By visual comparison of the maps, cultivars with similar distributions were detected to identify the trait correlations. Thus, this method was used to visualize and explore data properties and separate the data set into clusters of similar characteristics.

### DNA extraction and physical map construction

DNA isolation and genotyping were performed as reported elsewhere (Li *et al.*, 2019a; Fu *et al.*, 2020). A modified cetyltrimethyl ammonium bromide (CTAB) protocol was used to extract genomic DNA from young leaves. The DNA samples were genotyped with the Illumina 90K wheat SNP array (containing 81 587 SNPs) and the Affymetrix 660K wheat SNP array (containing 630 517 SNPs) by CapitalBio Technology Co., Ltd (http://www.capitalbiotech.com/). Heterozygous genotypes were considered as missing data. SNPs with minor allele frequency (MAF) <5 % and missing data >20 % were excluded from further analysis. Linkage disequilibrium (LD) among SNPs was analysed using the full matrix and sliding window options implemented in Tassel software (Version 5.0) by 12 323 SNPs evenly distributed on 21 wheat chromosomes. Population structure was analysed using 2000 evenly distributed polymorphic SNPs with the Structure software (Version 2.3.4). A neighbour-joining tree was constructed, and principal compenents analysis (PCA) was performed with Tassel to verify the population stratification (Liu *et al.*, 2017).

### Genome-wide association study

The GWAS was performed with the mean values of two replicates in each environment, the BLUE values across four environments for each trait, and the SNP markers from the wheat 90K and 660K SNP arrays. The analysis was conducted using GAPIT (Genome Association and Prediction Integrated Tool; Version 3). Kinship was set as the random effects and principal components (PCs) as fixed effects to control the population structure and familial relatedness. Manhattan plots and quantile–quantile plots (*Q*-*Q* plots) were generated using the R package ‘CMplot’. Bonferroni–Holm correction for multiple testing (α = 0.05) was too conserved for the present study’s traits. Therefore, markers with an adjusted −log_10_(*P*-value) ≥ 3.0 were considered to show significant marker–trait associations (MTAs) to maximize the chances of identifying all possible QTLs. This threshold was also used in some wheat association studies on complex traits (Gao *et al.*, 2015; Li *et al.*, 2018b; Fu *et al.*, 2020). The most significant SNP markers were chosen for each common locus as the representative markers. A locus detected in at least two environments was declared stable. Loci or QTLs reported in previous studies were considered the same as those in the present study if the tightly linked or significantly associated markers were within less than one LD.

### Analyses of allele frequencies and effects of alleles

QTL allelic frequencies were analysed based on the SNP at each QTL peak. The alleles increasing flag leaf length, flag leaf width, flag leaf area, flag leaf biomass, flag leaf specific leaf area, chlorophyll content SPAD meter reading, light-saturated net photosynthetic rate, stomatal conductance, intercellular CO_2_ concentration, transpiration rate, water use efficiency, intrinsic water use efficiency, maximum quantum yield of PSII photochemistry, leaf area index, grain filling rate, stay green trait, spike shape, leaf water content, spike water content, stem water content, leaf dry weights, spike dry weights, stem dry weights, total dry weights, spike number per square metre, kernel number per spike, thousand-grain weight and grain yield, but decreasing plant height, flag leaf angle, thermal time from sowing to flowering stage and thermal time from sowing to maturity stage, were defined as favourable alleles. QTL allelic frequencies and effects were analysed based on the representative markers of each locus. The average BLUE values for two genotypes at each locus were used to compare the subgroup effects by a *t*-test. The association panel was grouped based on the favourable alleles for each trait. Further, the correlation between the average phenotypic values of each group (average of BLUE values of accessions within each group) and the number of favourable alleles was analysed with the ‘rcorr’ function of the ‘Hmisc’ package in R (Version 3.6.2).

## RESULTS

### Phenotypic variation, heritability and contribution to yield of wheat traits

The 32 traits among 166 wheat accessions across the four environments showed substantial variations (Table 1), following a normal distribution (Supplementary Data Fig. S3). The coefficient of variation (CV) of phenotypic data ranged from 3.74 to 55.10 %, averaging 23.17 % across four environments. Large phenotypic trait diversity among the accessions is ideal for conducting GWAS. ANOVA revealed significant effects of genotype, environment and genotype × environment interactions on all the traits. Some traits presented high broad-sense heritabilities (*h*^2^), such as TDWS (0.91), PH (0.94), GFR (0.84), TTM (0.84), FLW (0.85), FLANG (0.84) and TGW (0.91), indicating that most of these traits were mainly controlled by genetic factors.

**Table 1.** Natural variation, broad-sense heritability and ANOVA for 32 traits across 166 wheat cultivars grown under four environments.

Trait	Descriptive analysis				Analysis of variance		
	Mean	SD	CV	*h* ^2^	Genotype (G)	Environment (E)	G × E interaction
LWC	0.615	0.123	19.921	0.513	0.009***	4.831***	0.013***
STWC	0.664	0.062	9.387	0.588	0.004***	0.830***	0.002***
SPWC	0.540	0.124	22.937	0.380	0.008***	4.167***	0.008***
LDWS	0.408	0.145	35.591	0.875	0.327***	4.528***	0.046***
STDWS	1.234	0.506	40.969	0.918	3.510***	33.430***	0.311***
SPDWS	0.668	0.290	43.418	0.850	0.617***	30.245***	0.107***
TDWS	2.300	0.870	37.824	0.914	8.828***	114.376***	0.827***
Pn	21.378	2.984	13.957	0.618	29.334***	157.363***	16.617***
Gs	0.406	0.123	30.196	0.567	0.0383***	397.838***	0.027***
Tr	4.377	1.167	26.655	0.483	1560.667***	103027.400***	1522.654***
Ci	277.696	28.952	10.426	0.561	2.948***	196.401***	2.048***
WUE	5.200	1.243	23.896	0.553	3.330***	229.081***	2.330***
iWUE	57.059	14.303	25.067	0.542	499.572***	17204.040***	372.040***
Fvʹ/Fmʹ	0.602	0.050	8.256	0.458	0.007***	0.542***	0.006***
PH	93.041	12.822	13.781	0.938	1096.407***	3067.014***	72.145***
LAI	4.527	0.581	12.831	0.634	1.191***	7.332***	0.590***
GFR	0.003	0.001	27.083	0.838	0.00***	0.00***	0.00***
SGT	0.401	0.221	55.104	0.655	0.153***	4.325***	0.077***
TTF	1748.705	67.611	3.866	0.825	15886.880***	606800.900***	3104.403***
TTM	2395.350	89.535	3.738	0.836	28217.510***	1723054.000***	5217.478***
SS	3.467	1.180	34.031	0.620	4.607***	5.442***	2.679***
FLL	19.985	4.534	22.689	0.797	47.135***	5541.081***	11.355***
FLW	1.700	0.239	14.047	0.852	0.256***	6.063***	0.043***
FLA	22.234	7.415	33.349	0.711	92.409***	14503.800***	34.508***
FLB	0.143	0.034	24.141	0.694	0.004***	0.014***	0.002***
FSLA	175.442	67.386	38.409	0.444	4404.124***	1116339.75***	5131.530***
FLANG	2.015	0.691	34.311	0.837	46.460***	642.406***	12.498***
SPAD	54.829	3.268	5.960	0.765	2.429***	9.524***	0.458***
SN	680.791	169.901	24.956	0.692	166.990***	1915.948***	15.897***
KN	40.327	5.516	13.678	0.505	71224.880***	6338188.000***	28664.100***
TGW	41.344	5.396	13.051	0.908	55.014***	4846.434***	48.658***
GY	3.396	0.612	18.035	0.868	1.375***	78.803***	0.191***

Mean, mean value; SD, standard deviation; CV, coefficient of variation; *h*^2^, broad-sense heritability. ***P < 0.001. LWC, leaf water content; SPWC, spike water content; STWC, stem water content; LDWS, leaf dry weights; SPDWS, spike dry weights; STDWS, stem dry weights; TDWS, total dry weights; Pn, light-saturated net photosynthetic rate; Gs, stomatal conductance; Tr, transpiration rate; Ci, intercellular CO_2_ concentration; WUE, water use efficiency; iWUE, intrinsic water use efficiency; Fvʹ/Fmʹ, maximum quantum yield of PSII photochemistry; PH, plant height; LAI, leaf area index; GFR, grain filling rate; SGT, stay green trait; TTF, thermal time from sowing to flowering stage; TTM, thermal time from sowing to maturity stage; SS, spike shape; FLL, flag leaf length; FLW, flag leaf width; FLA, flag leaf area; FLB, flag leaf biomass; FSLA, flag leaf specific leaf area; FLANG, flag leaf angle; SPAD, chlorophyll content SPAD meter reading; SN, spike number per square metre; KN, kernel number per spike; TGW, thousand-grain weight; GY, grain yield.

Correlation analysis for the BLUE value of each trait indicated that TGW was significantly and positively correlated with net photosynthetic rate (Pn), stomatal conductance (Gs), spike shape (SS), leaf water content (LWC), spike water content (SPWC), leaf dry weight (LDWS), flag leaf width (FLW) and SPAD, but negatively correlated with thermal time from sowing to flowering stage (TTF), flag specific leaf area (FSLA) and spike number (SN) (Supplementary Data Fig. S4). GY was significantly and positively correlated with net photosynthetic rate (Pn), stomatal conductance (Gs), transpiration rate (Tr), spike shape (SS), spike water content (SPWC), flag leaf width (FLW), SPAD and TGW, but negatively correlated with plant height (PH), flag leaf length (FLL), flag leaf angle (FLANG) and spike number (SN).

### Contributions of wheat traits to GY and TGW

**Fig. 1.**
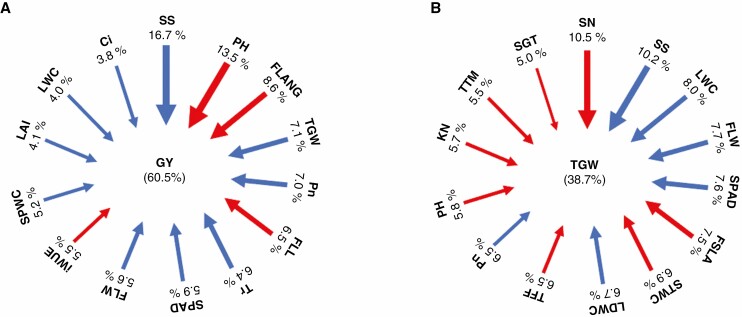
Random forest analysis predictors of grain yield (A) and TGW (B) in 209 wheat cultivars. The relative importance of each predictor is ranked in order and presented for GY (A) and TGW (B). Arrow colours indicate the direction of correlation (blue, positive; red, negative) for the continuous variables. The values represent the contribution of the trait grain yield or TGW. LWC, leaf water content; SPWC, spike water content; STWC, stem water content; LDWS, leaf dry weights; Pn, light-saturated net photosynthetic rate; Tr, transpiration rate; Ci, intercellular CO_2_ concentration; iWUE, intrinsic water use efficiency; PH, plant height; LAI, leaf area index; SGT, stay green trait; TTF, thermal time from sowing to flowering stage; TTM, thermal time from sowing to maturity stage; SS, spike shape; FLL, flag leaf length; FLW, flag leaf width; FSLA, flag leaf specific leaf area; FLANG, flag leaf angle; SPAD, chlorophyll content SPAD meter reading; SN, spike number per square meter; TGW, thousand-grain weight; GY, grain yield.

The relative importance of traits in determining GY and TGW was applied with a random forest model with 500 classification trees. Model accuracy was 60.5 and 38.7 % for GY and TGW, respectively (Fig. 1). Fourteen morphological and physiological traits conferring GY and TGW were selected based on the random forest results. Spike shape, plant height, flag leaf angle and thousand-grain weight were the most significant predictors of grain yield, accounting for 45.9 % of the total variation. Spike number, spike shape, leaf water content and flag leaf width were the most significant predictors of thousand-grain weight, accounting for 36.4 % of the total variation. Plant height, spike shape, photosynthetic rate, SPAD, flag leaf width and leaf water content contributed significantly to GY and TGW. Thus, the random forest model suggested that promoting photosynthesis traits, spike shape, SPAD and leaf water content and reducing plant height could improve GY and TGW.

### Genomic variation, LD and population structure

**Fig. 2.**
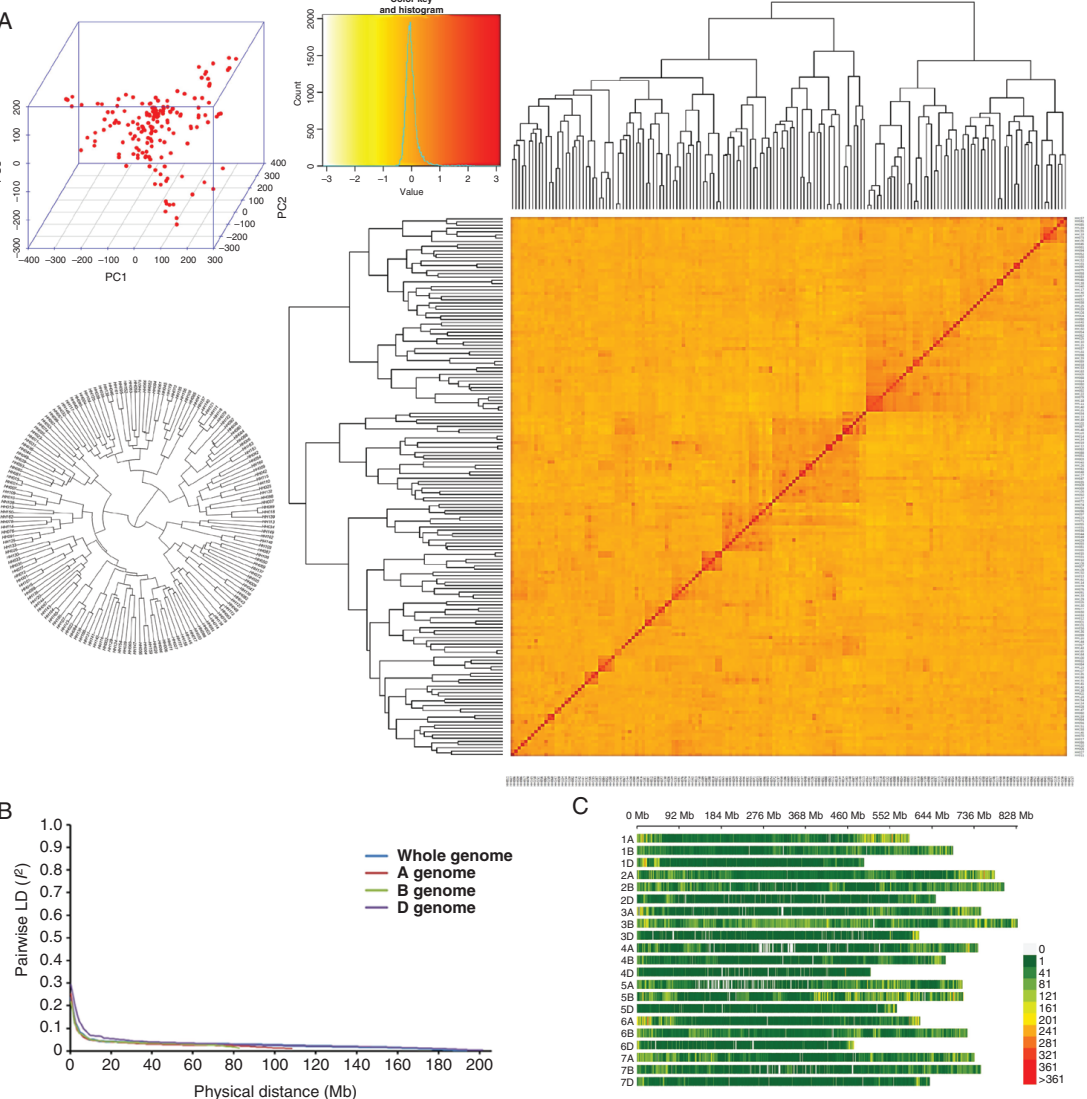
(A) Population structure of 166 wheat accessions revealed by principal component (top left), neighbour-joining tree (bottom left) and kinship (right) analyses. (B) Linkage disequilibrium (LD) decay across the whole genome and A, B and D sub-genomes and (C) distribution of SNPs with minor allele frequency >0.05 and missing data <80 %.

A total of 373 106 high-quality SNPs from the two SNP arrays were used for GWAS. Approximately 39.8, 49.3 and 10.8 % of the markers were in sub-genomes A, B and D, respectively. The number of SNPs per chromosome ranged from 2374 on 4D to 46 708 on 3B. These markers covered a total physical distance of 14 061.15 Mb, with a genome-wide average of 26 SNPs per Mb. The principal component, neighbour-joining phylogenetic tree and kinship analyses are presented in Fig. 2A–C, namely subgroup I (62 cultivars), subgroup II (54 cultivars) and subgroup II (50 cultivars). LD decay varied among the sub-genomes and across the chromosomes. LD decay in the B sub-genome dropped quickly. The average genome-wide extent of LD was 8 Mb, with an average of 6, 4 and 11 for the A, B and D sub-genomes, respectively.

### Genome-wide association studies

**Fig. 3.**
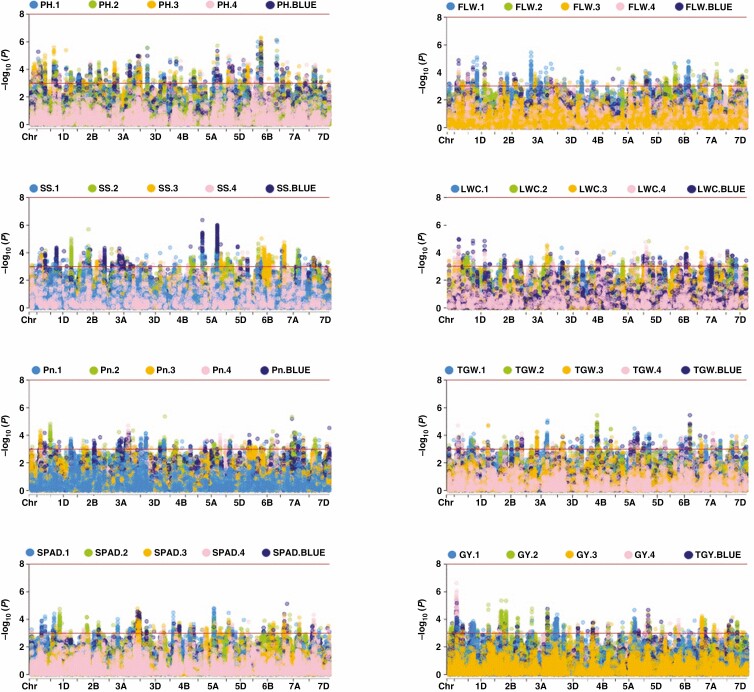
Manhattan plots for a genome-wide association study of the traits for GY and TGW in 166 wheat accessions under multiple environments. PH, plant height; SS, spike shape; Pn, light-saturated net photosynthetic rate; SPAD, chlorophyll content SPAD meter reading; FLW, flag leaf width; LWC, leaf water content; TGW, thousand-grain weight; GY, grain yield.

We performed a GWAS for photosynthetic, morphological and agronomic traits using the mixed linear model (MLM) method. These significant and stable SNPs were located on 21 chromosomes and explained 4.93–17.77 % of the phenotypic variance among environments (Fig. 3; Supplementary Data Fig. S5). A total of 1236 stable loci were detected for the 32 traits. In SNP-GWAS, 59, 42, 44, 54, 38, 42, 32, 19, 13, 8, 11, 9, 6, 7, 115, 34, 58, 24, 53, 34, 39, 28, 23, 6, 68, 67, 73, 70, 35, 25, 55 and 45 loci were detected for flag leaf length (FLL), flag length width (FLW), flag leaf area (FLA), flag leaf biomass (FLB), flag leaf specific area (FSLA), flag leaf angle (FLANG), SPAD, net photosynthetic rate (Pn), stomatal conductance (Gs), intercellular CO_2_ concentration (Ci), transpiration rate (Tr), water use efficiency (WUE), intrinsic water use efficiency (iWUE), maximum quantum yield of PSII photochemistry (Fvʹ/Fmʹ), plant height (PH), leaf area index (LAI), grain filling rate (GFR), stay green trait (SGT), thermal time from sowing to flowering stage (TTF), thermal time from sowing to maturity stage (TTM), spike shape (SS), leaf water content (LWC), spike water content (SPWC), stem water content (STWC), leaf dry weights (LDWS), spike dry weights (SPDWS), stem dry weights (STDWS), total dry weights (TDWS), spike number per square metre (SN), kernel number per spike (KN), thousand-grain weight (TGW) and grain yield (GY), respectively. Manhattan plots for 32 traits (Fig. 3; Fig. S5) using MLM in the four environments and BLUE values showed the location of SNPs and the associated SNPs, and *Q*-*Q* plots for the traits are shown in Fig. S6. The loci of 32 traits detected in at least two out of the five environments (including BLUE) are summarized in Table S2. To conclude, the GWAS results are reliable and efficient in detecting the loci for GY and related traits.

### Pleiotropic loci

**Fig. 4.**
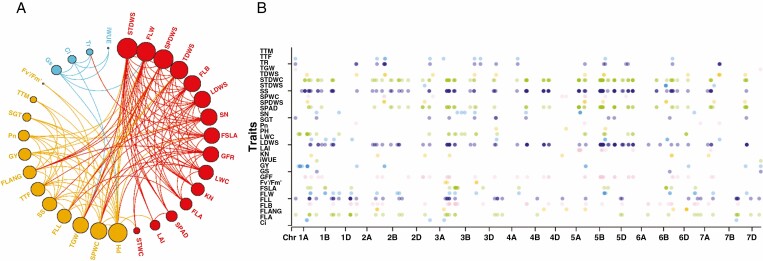
The pleiotropic loci for traits of both traits controlled by the same SNP (A) and the position of pleiotropic loci (B). LWC, leaf water content; SPWC, spike water content; STWC, stem water content; LDWS, leaf dry weights; SPDWS, spike dry weights; STDWS, stem dry weights; TDWS, total dry weights; Pn, light-saturated net photosynthetic rate; Gs, stomatal conductance; Tr, transpiration rate; Ci, intercellular CO_2_ concentration; WUE, water use efficiency; iWUE, intrinsic water use efficiency; Fvʹ/Fmʹ, maximum quantum yield of PSII photochemistry; PH, plant height; LAI, leaf area index; GFR, grain filling rate; SGT, stay green trait; TTF, thermal time from sowing to flowering stage; TTM, thermal time from sowing to maturity stage; SS, spike shape; FLL, flag leaf length; FLW, flag leaf width; FLA, flag leaf area; FLB, flag leaf biomass; FSLA, flag leaf specific leaf area; FLANG, flag leaf angle; SPAD, chlorophyll content SPAD meter reading; SN, spike number per square metre; KN, kernel number per spike; TGW, thousand-grain weight; GY, grain yield.

We further investigated the pleiotropic loci for these traits (Fig. 4). A total of 47 pleiotropic loci were associated with three or more traits and GY/TGW on chromosomes 1A (7 loci), 1B (2 loci), 1D (2 loci), 2A (3 loci), 2B (4 loci), 3A (2 loci), 3B (4 loci), 3D, 4A, 4B (4 loci), 4D, 5A (3 loci), 5B (4 loci), 6B (3 loci), 6D, 7A (2 loci), 7B and 7D (2 loci) based on the common loci detected by GWAS (Supplementary Data Table S3). The interval 703.20–708.77 Mb on chromosome 5A was associated with plant height (PH), flag leaf angle (FLANG), GY, TGW, stem dry weights (STDWS), flag leaf biomass (FLB), leaf dry weights (LDWS), stem dry weights (STDWS) and total dry weights (TDWS), sharing the same region with GY. Twenty-three pleiotropic loci were associated with GY, among which 12 were related to plant height (PH), seven to flag leaf length (FLL) and six to grain filling rate (GFR). Seven spike shape (SS) loci on chromosomes 1A (*GENE_1785_626*), 1B (*AX_109097017*), 2B (*AX_109602295*), 3A (*AX_108765521*), 4B (*AX_111005064*) and 5A (*AX_108899874*, *RAC875_c18335_443*) were located in pleiotropic loci. Four net photosynthetic rate (Pn) loci on chromosomes 1A (*AX_109095224*), 2A (*AX_111046029*), 3A (*AX_108765521*) and 3D (*AX_111656541*) were also located in pleiotropic loci. Our observations indicated that 11 pleiotropic loci for the thermal time from sowing to flowering stage (TTF) or flag leaf length (FLL), nine for stem dry weights (STDWS), and four for spike dry weights (SPDWS) or SPAD are crucial in determining GY or TGW.

### Distributions and the cumulative effect of favourable alleles

Favourable allele frequencies of the identified QTLs associated with 32 traits ranged from 0.05 to 0.94 (average 0.48). The frequencies of the favourable allele of QTLs for net photosynthetic rate (Pn), stomatal conductance (Gs), intercellular CO_2_ concentration (Ci), water use efficiency (WUE), flag length width (FLW), flag leaf angle (FLANG), plant height (PH), leaf area index (LAI), thermal time from sowing to flowering stage (TTF), spike shape (SS), leaf water content (LWC), spike water content (SPWC), stem water content (STWC), TGW and GY were higher (average from 0.53 to 0.92) than those for transpiration rate (Tr), intrinsic water use efficiency (iWUE), maximum quantum yield of PSII photochemistry (Fvʹ/Fmʹ), flag leaf length (FLL), flag leaf area (FLA), flag leaf biomass (FLB), flag leaf specific area (FSLA), SPAD, grain filling rate (GFR), stay green trait (SGT), thermal time from sowing to maturity stage (TTM), leaf dry weights (LDWS), spike dry weights (SPDWS), stem dry weights (STDWS), total dry weights (TDWS), spike number per square metre (SN), kernel number per spike (KN) and GW (0.18–0.49). These results indicate that most of the accessions possessed alleles that increased net photosynthetic rate (Pn), stomatal conductance (Gs), intercellular CO_2_ concentration (Ci), water use efficiency (WUE), flag length width (FLW), leaf area index (LAI), spike shape (SS), leaf water content (LWC), spike water content (SPWC), stem water content (STWC), TGW and GY, but reduced flag leaf angle (FLANG), plant height (PH) and thermal time from sowing to flowering stage (TTF) (Supplementary Data Fig. S7).

**Fig. 5.**
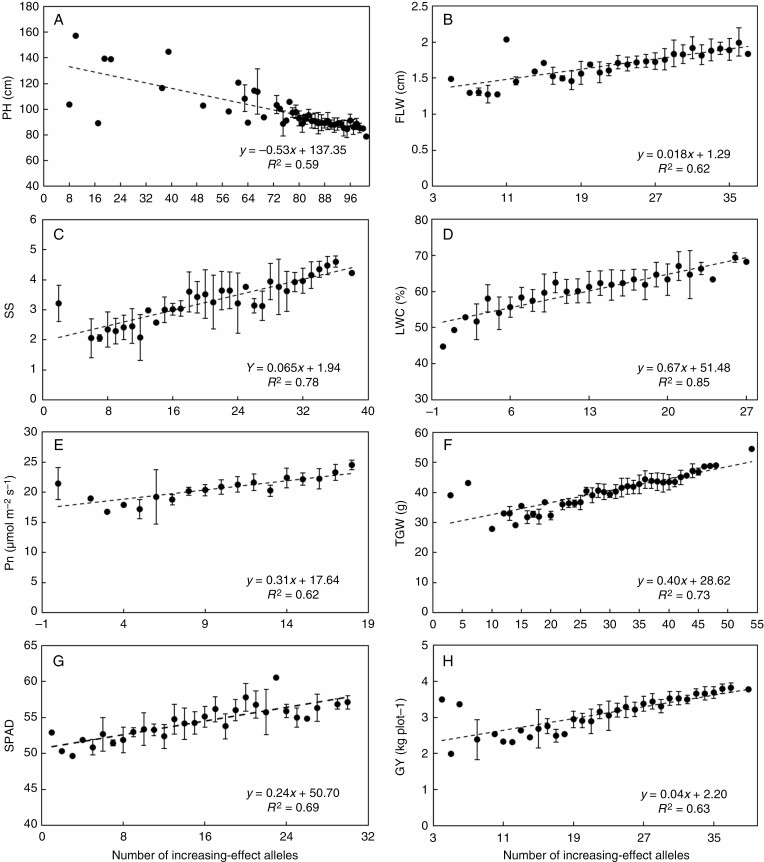
Effect and distribution of favourable alleles of trait-associated markers contributing significantly to GY and TGW. PH, plant height; SS, spike shape; Pn, light-saturated net photosynthetic rate; SPAD, chlorophyll content SPAD meter reading; FLW, flag leaf width; LWC, leaf water content; TGW, thousand-grain weight; GY, grain yield.

The number of increasing-effect alleles in each accession was simulated further to investigate the effects of combined alleles on those traits. Linear regression analysis was performed using the BLUE values to further investigate the relationships between trait values and the number of trait-increasing alleles. Favourable alleles for 32 traits at each locus showed significant and positive effects on the phenotypic trait values (Fig. 5; Supplementary Data Fig. S8). Significant correlations (*P* < 0.01) were observed between trait values and the number of trait-increasing alleles. For many traits, the coefficients of determination (*R*^2^) between the trait’s values and the number of favourable alleles in each accession were >0.62. This result suggests that QTLs with additive effects controlled many traits. Stem water content (STWC) and thermal time from sowing to flowering stage (TTF) had an *R*^2^ < 0.54, indicating that the environment affected the expression of QTLs more for these traits.

### Genetic progress

**Fig. 6.**
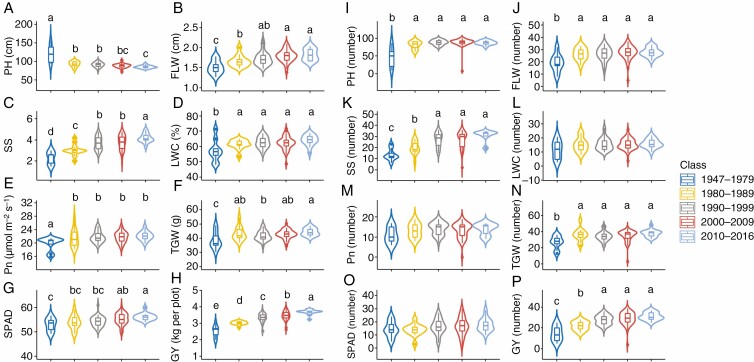
Genetic progress of the traits contributing significantly to GY and TGW. Violin plots A–H, phenotypic changes in PH, FLW, SS, LWC, Pn, TGW, SPAD and GY, respectively; I–P, changes in number of increasing-effect alleles for PH, FLW, SS, LWC, Pn, TGW, SPAD and GY, respectively. PH, plant height; SS, spike shape; Pn, light-saturated net photosynthetic rate; SPAD, chlorophyll content SPAD meter reading; FLW, flag leaf width; LWC, leaf water content; TGW, thousand-grain weight; GY, grain yield. Different lowercase letters indicate significantly different at 0.05 level.

The genetic progress of 32 traits was investigated to explore the role of yield-associated loci in improving GY (Fig. 6; Supplementary Data Fig. S9). The cultivars released after 2010 (13) demonstrated an increase in Pn (11.78 %), Gs (36.62 %), Tr (29.89 %), Ci (7.65 %), Fvʹ/Fmʹ (4.83 %), LAI (6.51 %), SGT (10.43 %), LWC (11.87 %), SPWC (10.05 %), LDWS (26.21 %), SPDWS (27.71 %), 5.69 % (TDWS), FLW 20.28 %), FLB (2.89 %), SPAD (5.97 %), KN (4.92 %) and TGW (15.50 %) relative to the cultivars released before 1980 (nine), accompanied by a 47.1 % increase in GY. However, the cultivars released after 2010 (13) showed a decrease in WUE (13.48 %), iWUE (20.14 %), PH (29.65 %), GFR (14.21 %), STDWS (6.80 %), FLL (13.06 %), FLA (11.47 %), Pn (11.78 %), FSLA (15.50 %), FLANG (43.04 %) and SN (15.99 %) relative to the cultivars released before 1980 (nine). In addition, the year of release (five groups) had a significant effect on the number of favourable alleles for phenotypic traits, such as plant height (PH), spike shape (SS), flag length width (FLW), spike number per square metre (SN), TGW and GY, but not net photosynthetic rate, leaf area index (LAI), stay green trait (SGT), thermal time from sowing to maturity stage (TTM), leaf water content (LWC) or biomass-related traits (Fig. 6; Fig. S10).

### Ideotypes for wheat breeding

**Fig. 7.**
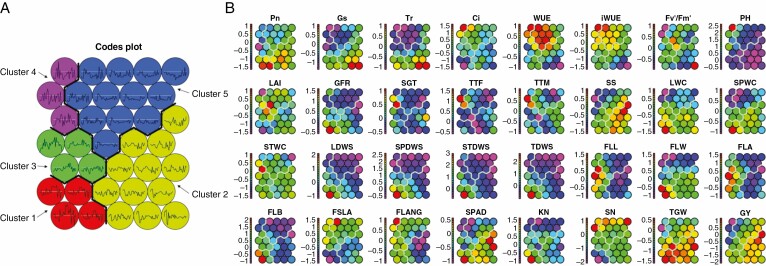
Clustering of 166 wheat cultivars using a self-organizing map (SOM) algorithm (A) and the matrices of components (B). LWC, leaf water content; SPWC, spike water content; STWC, stem water content; LDWS, leaf dry weights; SPDWS, spike dry weights; STDWS, stem dry weights; TDWS, total dry weights; Pn, light-saturated net photosynthetic rate; Gs, stomatal conductance; Tr, transpiration rate; Ci, intercellular CO_2_ concentration; WUE, water use efficiency; iWUE, intrinsic water use efficiency; Fvʹ/Fmʹ, maximum quantum yield of PSII photochemistry; PH, plant height; LAI, leaf area index; GFR, grain filling rate; SGT, stay green trait; TTF, thermal time from sowing to flowering stage; TTM, thermal time from sowing to maturity stage; SS, spike shape; FLL, flag leaf length; FLW, flag leaf width; FLA, flag leaf area; FLB, flag leaf biomass; FSLA, flag leaf specific leaf area; FLANG, flag leaf angle; SPAD, Chlorophyll content SPAD meter reading; SN, spike number per square meter; KN, kernel number per spike; TGW, thousand-grain weight; GY, grain yield.

Data for the 32 wheat traits were inputted into the SOM system to classify the traits for wheat ideotype breeding based on the 166 wheat lines. The number of nodes was set as 35, the number of rows as eight and the number of columns as five. The SOM results are presented in Supplementary Data Table S4 and Fig. 7. Figure 7A shows five different clusters with similar composition of trait values. Figure 7B shows 32 component maps representing the component values by visually identifying the correlation among traits for the 35 nodes. Clusters 1, 2, 3, 4 and 5 consisted of 13, 60, 20, 15 and 58 cultivars, respectively. Cultivars in Cluster 1 were characterized by high GY, TGW, net photosynthetic rate (Pn), stomatal conductance (Gs), transpiration rate (Tr), intercellular CO_2_ concentration (Ci), maximum quantum yield of PSII photochemistry (Fvʹ/Fmʹ), grain filling rate (GFR), stay green trait (SGT), thermal time from sowing to maturity stage (TTM), flag length width (FLW), SPAD, spike shape (SS), leaf water content (LWC), spike water content (SPWC), stem water content (STWC), leaf dry weights (LDWS), spike dry weights (SPDWS), stem dry weights (STDWS), total dry weights (TDWS), flag leaf biomass (FLB) and kernel number per spike (KN) values; low water use efficiency (WUE), intrinsic water use efficiency (iWUE), plant height (PH), leaf area index (LAI), thermal time from sowing to flowering stage (TTF), flag leaf specific area (FSLA) and spike number per square metre (SN) values; and median flag leaf length (FLL), flag leaf angle (FLANG) and flag leaf area (FLA) values. These traits were relatively important in determining grain yield and thousand weight. The selected top-performing cultivars (Sunong 6, Zheng 9023, Zhoumai 30, Zhou 8425B, Zimai 12, Lumai 23, Linmai 2, Lankao 906, Linmai 4, Jining 16, Wanmai 33 and Lankao 2) were from the Yellow and Huai Valleys Winter Wheat Zone where the trials were conducted, suggesting that these wheat lines have some adaptability to different environments. Our findings suggest the use of cultivars in Cluster 1 as potential parents to develop high-yielding cultivars with desirable traits.

## DISCUSSION

### Natural variation of all traits

MAS has several advantages over conventional breeding, constrained mainly by time and resources; however, the desirable traits originate mostly by chance without fully understanding the underlying physiological mechanisms (Fischer *et al.*, 1998). Complex morphological and physiological traits at the tissue or whole-plant level led to high RUE; therefore, a comprehensive evaluation based on multiple traits can help analyse yield formation and screen germplasm for breeding (Ding *et al.*, 2018; Li *et al.*, 2019a). Moreover, there is still much to learn from the natural variation in photosynthetic capacity and performance, which demands further exploration of the underlying physiological and genetic mechanisms among species and cultivars (Flood *et al.*, 2011). A previous study indicated underutilized photosynthetic capacity among existing wheat cultivars using 64 elite wheat cultivars (Driever *et al.*, 2014). Although introgressions to improve photosynthesis have been performed since the 1960s, the process remains quite limited (Ojima, 1974). The limited availability of critical targets and well-defined molecular markers associated with traits have constrained breeding programmes (Flood *et al.*, 2011). Our study is the largest investigation of the phenological, physiological, plant architectural and yield-related traits of wheat to date. We identified 14 critical traits with the random forest model as predictors of grain yield variations, and therefore as potential traits for breeding consideration.

Intraspecific crop diversity could be a valuable biological resource for better understanding and maintaining crop resilience to extreme weather (Heider *et al.*, 2021). We aimed to uncover the natural variation among 166 wheat cultivars to identify physiological parameters and morphological traits significantly correlated with biomass accumulation and yield, laying a foundational framework for gene discovery. The loci for plant height were associated with many traits in this study (Fig. 4). The QTLs for height are coincident with those for grain yield (Foulkes *et al.*, 2011). Reduced plant height during the Green Revolution may have been accompanied by screening for traits favouring yield accumulation. Our data confirmed substantial variations among the different traits under four environmental conditions, indicating genetic diversity that can be potentially exploited for wheat yield improvement. Besides, the high heritability (*h*^2^) of many traits observed in this study suggests their potential in wheat breeding, which requires identification of the associated candidate QTLs for MAS. Therefore, conducting a GWAS for high RUE using an SNP array will enrich the genotype–phenotype map and identify valuable gene resources for molecular breeding to enhance productivity in the face of increasing food demand (Li *et al.*, 2020).

### Role of photosynthesis in yield gains

Wheat yield potential has previously increased mainly due to improvements in harvest index rather than increased biomass. Although further large increases in HI are unlikely, there is an opportunity to increase productive biomass and harvestable grain (Reynolds *et al.*, 2011; Li *et al.*, 2022a). Even small increases in net photosynthesis rate could result in large increases in biomass and, thus, yield. Therefore, increasing photosynthetic capacity and efficiency are bottlenecks to improving crop biomass production and yield potential (Zhu *et al.*, 2010; Qu *et al.*, 2017). Improvements in features related to canopy architecture, such as semidwarf architecture, more erect leaves and larger LAI, have played a significant role in traditional crop breeding; however, no studies have addressed the improvement in leaf photosynthetic capacity per se (Peng *et al.*, 1999; Hedden, 2003). Our random forest models demonstrated that net photosynthetic rate and SPAD contributed greatly to GY and TGW (Fig. 1). The SPAD measurement can be utilized as an appropriate tool for photosynthetic genetic analysis (Takai *et al.*, 2010). Therefore, complex physiological traits related to photosynthetic efficiency must be incorporated as additional criteria to accelerate the genetic gains related to yield.

Previous studies that assessed the variations in wheat traits measured limited germplasm samples (Xue *et al.*, 2002; Sadras *et al.*, 2012) and used different experimental approaches (Chytyk *et al.*, 2011). Only a few researchers have reported QTLs associated with photosynthetic characteristics (Teng *et al.*, 2004; Adachi *et al.*, 2011), probably because the measurement of gas exchange parameters is laborious. In addition, the environment, especially the microclimate during growth and measurement, inevitably fluctuates under natural conditions and affects the phenotypic values (Flood *et al.*, 2011; Gu *et al.*, 2012). Therefore, in this study, we used a statistical approach via VPD to correct for microclimate variations. Based on this, we identified a significant positive correlation between photosynthesis rate and stomatal conductance, indicating that photosynthesis rate and stomatal conductance were significantly correlated with GY and TGW. In a panel of 215 elite rice cultivars, crop biomass accumulation was positively correlated with photosynthetic traits (Qu *et al.*, 2017). However, no correlation was found between photosynthetic capacity and GY in 64 elite wheat cultivars (Driever *et al.*, 2014).

Biomass accumulation, an integrative measure of carbon fixation by above-ground structures, can indicate canopy photosynthesis (Qu *et al.*, 2017). A previous study indicated that improved water use efficiency might be related to improved radiation interception efficiency, photosynthetic efficiency and a longer green-canopy duration (stay-green traits), all of which were found to be improved in modern cultivars (Voss-Fels *et al.*, 2019). Our results indicated Pn was positively correlated with LDWS and SPDWS, and TGW was positively correlated with LDW, SPDWS, STDWS and TDWS. Meanwhile, chlorophyll fluorescence parameters directly or indirectly reflect plant photosynthesis and are important for investigating the functional mechanism of PSII (Baker, 2008; Liang *et al.*, 2009; Yin *et al.*, 2010). There is a positive correlation between relative chlorophyll fluorescence in the leaf and GY (Von Korff *et al.*, 2008). However, the current study revealed a positive correlation between the chlorophyll fluorescence parameter Fvʹ/Fmʹ and LWC, despite the experiments being conducted under well-watered conditions. The changes in photosynthetic characteristics associated with breeding progress are valuable benchmarks for further improvement (Sadras *et al.*, 2012). Furthermore, exploiting existing natural variation in photosynthetic capacity and biomass accumulation laid a foundation for increasing yield. Our results indicated GY increased linearly from 1957 to 2016, but had no significant correlation with Pn or biomass, indicating photosynthesis rate could be further exploited.

### Wheat ideotypes

A feasible method to improve yield potential is the development of new cultivars (Sakuma & Schnurbusch, 2020). However, the genetic basis of grain yield is largely unclear, and the application of MAS for grain yield in current breeding remains limited. The breeding of crop ideotypes was first proposed by Donald (1968). Understanding the basic physiology of the traits associated with yield potential can guide hybridization and selection strategies for these relatively heritable constituent parameters (Reynolds *et al.*, 2011). Yield gains are presumably a function of improved coordination among physiological agronomic traits (He & Bonjean, 2010; Valluru *et al.*, 2017; Abbai *et al.*, 2020). The results indicated that these traits contributed to yield formation to some extent. Studies have suggested that the best plant architecture consists of more erect leaves in the upper canopy, and more horizontal leaves in the middle and lower canopy (Parry *et al.*, 2011; Ort *et al.*, 2015). Consistent with previous studies, our findings demonstrated a positive correlation between vertical flag leaf angle and GY. However, SOM results showed that the ideal plant architectural flag leaf angle was not the lowest when multiple traits were considered, indicating specific feedback among characteristics (Reynolds *et al.*, 2015; Li *et al.*, 2020). It is noteworthy that Donald’s ideotypes and our study focused on above-ground parts, while roots are important and main organs that absorb water and minerals and detect soil stress signals (Paez-Garcia *et al.*, 2015). Their traits determine absorption capacity and stress response (Fang *et al.*, 2017; Li *et al.*, 2019b). Therefore, root traits and the trade-offs strategies between above- and below-ground traits need to be further studied in the future.

Another way to increase wheat yield productivity is to optimize the source–sink ratio (Bustos *et al.*, 2013). In the current study, the ideal type confirmed a high correlation between the sink (KN, TGW and GY) and the source (Pn, SPAD and Fvʹ/Fmʹ). Increasing grain filling duration for more light interception and utilization will also improve yield potential (Brooks *et al.*, 2001; Semenov & Stratonovitch, 2013). For wheat, flag leaves account for 45–58 % of the photosynthetic performance during the grain-filling stage (Khaliq *et al.*, 2008). Earlier flowering should be improved in parallel in areas with high temperatures around 40% of the temperature zones experience high temperatures of more than 30 °C during grain filling that affects grain filling rate and final yield (Rane *et al.*, 2007; Reynolds *et al.*, 2011). Previous studies have suggested that early flowering could shift the grain-filling period to relatively cooler conditions to avoid heat stress, leading to increased reproductive growth duration and wheat yield (Tao & Zhang, 2013; Tao *et al.*, 2017b). Our current findings indicated that TGW and GY were significantly and positively correlated with spike shape, but negatively correlated with spike number. The lanceolate panicle was related to high grain yield. A trade-off exists in grain weight and grain number (Sadras, 2007; Sadras and Slafer, 2012; Xiao *et al.*, 2022). We found a positive correlation between plant height and grain number (Supplementary Data Fig. S3). A previous report from a similar environment has suggested that significant improvement in yield was mainly because grain weight contributes to yield potential and stability (Zheng *et al.*, 2011).

Plant water status is another important factor that plays a critical role in yield formation. Maintaining a certain relative water content could improve GY and stability (Matin *et al.*, 1989; Teulat *et al.*, 2003). Physiological and morphological traits, such as flag leaf photosynthesis, transpiration and yield, are closely related to plant water status (González *et al.*, 2008). We tried to keep the same field management under well-watered conditions in our experiment. LWC and SPWC showed a significant relationship with TGW and GY. To conclude, the best-yielding cultivars revealed higher water content, early flowering with longer reproductive growth duration, higher flag leaf width, photosynthetic capacity, high grain filling rate and stay-green features but lower flag leaf length, flag leaf angle, plant height and spike numbers. Our findings provide information about ideotypic traits, supporting breeding of climate-resilient crop cultivars.

### Genetic correlation between grain yield and yield-related traits

Combined phenomics and genomic methods are necessary to evaluate the progress of breeding strategies (Rizzo *et al.*, 2022; Welcker *et al.*, 2022). Studies have reported GY-related QTLs on all 21 wheat chromosomes (Kumar *et al.*, 2007; Wang *et al.*, 2011a; Azadi *et al.*, 2015; Sukumaran *et al.*, 2015). Our findings identified 45 stable loci for GY. The 1A locus (*AX_110507437*) for GY in our present study is at a similar position to gwm357 on the consensus linkage map (Maccaferri *et al.*, 2015), indicating that these two loci are probably the same. Six loci (*AX_110387060*, *AX_110418502*, *AX_94546135*, *AX_111210290*, *AX_111492146* and *AX_95257733*) for GY (Li *et al.*, 2018a) were consistent with those identified in this study on chromosomes 1A, 1A, 2A, 2B, 3A and 3D, respectively. Maccaferri *et al* (2015) also detected loci related to KN on chromosomes 1A, 1B, 2A, 2B, 2D, 3A, 4B, 5A, 5B, 6B, 7A and 7B; the loci on 1A, 1B, 2A, 2D, 3A and 5A are likely to be the same as *AX_111579941*, *IWB52449*, *IWB58750*, *IWB57054*, *AX_108992368* and *IWB8258* in our study since they are located in the same positions. The 1A QTL has a position similar to another KN locus identified in our study (*AX_108992368*) (Azadi *et al.*, 2015; Gao *et al.*, 2015). The locus *AX_111579941* on 1A is about one LD from a QTL (Wang *et al.*, 2011a). In addition, loci related to TGW such as *AX_111102999*, *AX_109862024*, *GENE_1785_118*, *AX_111548406*, *AX_110958315*, *AX_108769612*, *AX_94761192* and *AX_108929087* on 2B, 2B, 3B, 4B, 5A, 5B, 6B and 6B were found at similar positions (Li *et al.*, 2018a). The locus *AX_110958315* on 5A is about one LD from a TGW-related QTL (Gao *et al.*, 2015). By contrast, the locus *AX_111548406* on 4B for TGW was identified at a similar position (Liu *et al.*, 2014). As for flag leaf traits, a pleiotropic locus for FLL and FLA is at the same position on chromosome 2B as *AX_111634394* (Wu *et al.*, 2016). Six identical loci to our study were on chromosomes 1A, 1D, 2A, 2B, 3A, 4A, 4D, 5B, 7A and 7B for FLL (Li *et al.*, 2018a). An FLL-related QTL on chromosome 2B overlapped with our FLL locus *AX_111634394* (Wu *et al.*, 2016). Identified *Qflw-3A* (FLW), *Qflw-7D* (FLW) and *Qfla-3A* (FLA) associated with flag leaf morphology are at positions similar to *AX_94541532*, *AX_110227474* and *AX_109838665* in our study (Yan *et al.*, 2020), respectively. The loci *AX_94541532* and *AX_111476049* are the same as FLW-QTLs on chromosome 6B (Wu *et al.*, 2016). In addition, some *Rht* loci can cause a reduction in plant height, which has an impact on grain yield (Xiao *et al.*, 2022). *Rht-D1b* is widely present in the Yellow and Huai Valleys Winter Wheat Zone (Gao *et al.*, 2017). The locus *AX_89703298* related to PH on chromosome 4D of this study is at the same position as *Rht-D1* (Xue *et al.*, 2002), indicating the influence of *Rht-D1b* on PH in the current study (Gao *et al.*, 2015; Sun *et al.*, 2017; Li *et al.*, 2018a). Loci *AX_94638736* and *AX_110476771* on chromosome 2A are at a position similar to *QUIL.caas-2AS.1* and *QPH.caas-2AL* (co-localized with *QUIL.caas-2AL*), respectively (Li *et al.*, 2018a). QTLs for PH were identified on 3A, close to our locus *AX_108900466* related to PH (Cui *et al.*, 2011; Lopes *et al.*, 2012). The 5A locus *BobWhite_c47401_491* is about one LD from a QTL (Li *et al.*, 2016), and near *Rht12* (Reynolds *et al.*, 2015). *Vrn-B1* is at a position similar to locus *AX_94715126* (Fu *et al.*, 2005). However, there is no reported correlation between PH and vernalization. The remaining loci are likely to be potentially novel MTAs responsible for PH. These identified significant SNPs indicated the reliability of the GWAS.

Crop yield is a quantitative trait controlled by many other plant traits, mainly polygenic in nature (Wu *et al.*, 2012; Li *et al.*, 2020). The potential yield increase associated with these traits remains relatively untapped. In this study, a significant correlation was observed between the number of favourable alleles and yield-related traits, suggesting that pyramiding the favourable alleles effectively could improve those traits and support breeding of ideotypes (Fig. 5). The GWAS of our study was also reliable and efficient in detecting loci for GY and related traits. Theoretical analysis indicated that a 50 % increase in wheat yield potential is possible through genetic improvement of RUE. A crop’s structural and reproductive aspects must be ameliorated in parallel to achieve the desired impact (Ort *et al.*, 2015). We found that the loci related to GY and TGW were pleiotropically correlated with PH, photosynthetic capacity, flag leaf traits, GFR and SS. Through co-localization, early flowering is likely to benefit TGW at lower temperatures. In addition, the year of release had a significant effect on some easily observed agronomic traits (PH, FLW, FLANG, TGW and GY), but no effect on complex traits (Pn, Gs, GFR, SGT and biomass traits), suggesting that there should be still opportunities for further improvement of those traits. Thus, our study identifies cultivars with favourable alleles as potential parents and molecular markers closely linked to the above QTLs for assisted selection to combine more favourable alleles and develop new wheat cultivars with ideal traits.

## CONCLUSIONS

Our GWAS is shown to be a powerful approach for genetic dissection of phenological, physiological, plant architectural and yield-related traits in wheat based on a high-resolution physical map. For the first objective, grain yield was positively correlated with the net photosynthetic rate, spike shape and thousand-grain weight but negatively correlated with plant height, flag leaf angle and spike number per square metre. For the second objective, 1236 significant stable loci associated with these were traits identified by SNP-GWAS, and which provide invaluable sources. Linear regression indicated apparent cumulative effects of favourable alleles for increasing corresponding traits. For the third objective, complex traits such as Pn could be further used to break through the current wheat yield plateau. The identified QTLs had considerable additive effects on trait performance (Fig. 5) with predictable phenotypes; thus, these QTLs have great potential to be exploited in breeding new high-yielding cultivars. For the fourth objective, 11 Chinese cultivars could be used as parents for new high-yielding cultivars with desirable traits. Our study highlighted that variations in different traits, their associations with grain yield and their genetic basis are important for yield improvement through MAS.

## SUPPLEMENTARY DATA

Supplementary data are available online at https://academic.oup.com/aob and consist of the following. Fig. S1: Correlation between net photosynthesis, intercellular CO_2_ concentration, stomatal conductance, transpiration rate and vapour pressure deficit VPD or Tleaf. Fig. S2: Image information and description of different spike shapes. Fig. S3: Phenotypic variations for the 32 wheat traits measured under multiple environments. Fig. S4: Correlation of 32 wheat traits by best linear unbiased estimations for each trait across four environments. Fig. S5: Manhattan plots for a genome-wide association study of the traits in 166 wheat accessions under multiple environments. Fig. S6: Quantile–quantile plots of GWAS for 32 traits. Fig. S7: Favourable allele frequencies of the identified QTLs. Fig. S8: Effect and distribution of favourable alleles of trait-associated markers. Fig. S9: Phenotypic changes in the trait values for 166 wheat cultivars released over the past 70 years. Fig. S10: Changes in numbers of increasing-effect alleles for 32 trait values over the past 70 years. Table S1: Information of the 166 wheat accessions used in GWAS. Table S2: Loci for 32 traits identified by GWAS. Table S3: Distribution of pleiotropic loci associated with three or more grain yield-related traits on wheat chromosomes. Table S4: 32 wheat trait values within each cluster.

mcad003_suppl_Supplementary_Material
