# Supplementary material for: Variations in phenological, physiological, plant architectural and yield-related traits, their associations with grain yield and genetic basis
Source: Ann Bot. 2023 Jan 19;131(3):503–19. doi: 10.1093/aob/mcad003 (PMC10072080; doi:10.1093/aob/mcad003)
Supplement: mcad003_suppl_Supplementary_Material [file mcad003_suppl_supplementary_material.docx]

**Supplementary materials**

**Variations in** **wheat phenological, physiological, plant architectural and yield-related traits, their associations with grain yield and genetic basis**

Supplementary material for this article is available at …

**Figure contents:**

**Fig. S1.** Correlation between net photosynthesis (Pn, umol CO_2_ m^-2^ s^-1^), intercellular CO_2_ concentration (Ci, umol CO_2_ m^-2^s^-1^), stomatal conductance (Gs, mol H_2_O m^-2^ s^-1^), transpiration rate (Tr, mmol H_2_O m^-2^ s^-1^) and vapour pressure deficit VPD (the left column of plots) or Tleaf (The right column of plots).

**Fig. S2.** The information on the image and description of different spike shapes.

**Fig. S3.** Phenotypic variations for the 32 wheat traits measured under multiple environments.

**Fig. S4.** Correlation of 32 wheat traits by best linear unbiased estimations for each trait across four environments.

**Fig. S5.** Manhattan plots for a genome-wide association study of the traits in 166 wheat accessions under multiple environments.

**Fig. S6.** Quantile - Quantile (Q-Q) plots of GWAS for 32 traits.

**Fig. S7.** Favorable allele frequencies of the identified QTLs.

**Fig. S8.** Effect and distribution of favorable alleles of trait-associated markers.

**Fig. S9.** Phenotypic changes in the traits values for 166 wheat cultivars released over the past 70 years (1947-2016).

**Fig. S10.** Changes in numbers of increasing-effect alleles for 32 traits values over the past 70 years (1947-2016).

**Table contents:**

**Table S1**. Information of the 166 wheat accessions used in GWAS.

**Table S2**. Loci for 32 traits identified by GWAS.

**Table S3**. Distribution of pleiotropic loci associated with three or more grain yield related traits on wheat chromosomes.

**Table S4**. 32 wheat traits values within each cluster (1, 2, 3, 4 and 5).


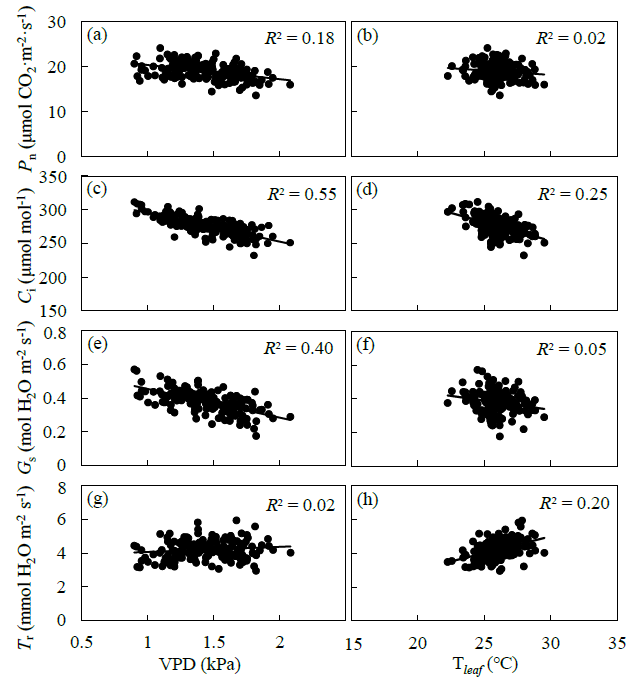


**Fig. S1.** Correlation between net photosynthesis (Pn, umol CO_2_ m^-2^ s^-1^), intercellular CO_2_ concentration (Ci, umol CO_2_ m^-2^s^-1^), stomatal conductance (Gs, mol H_2_O m^-2^ s^-1^), transpiration rate (Tr, mmol H_2_O m^-2^ s^-1^) and vapour pressure deficit VPD (the left column of plots) or Tleaf (The right column of plots).


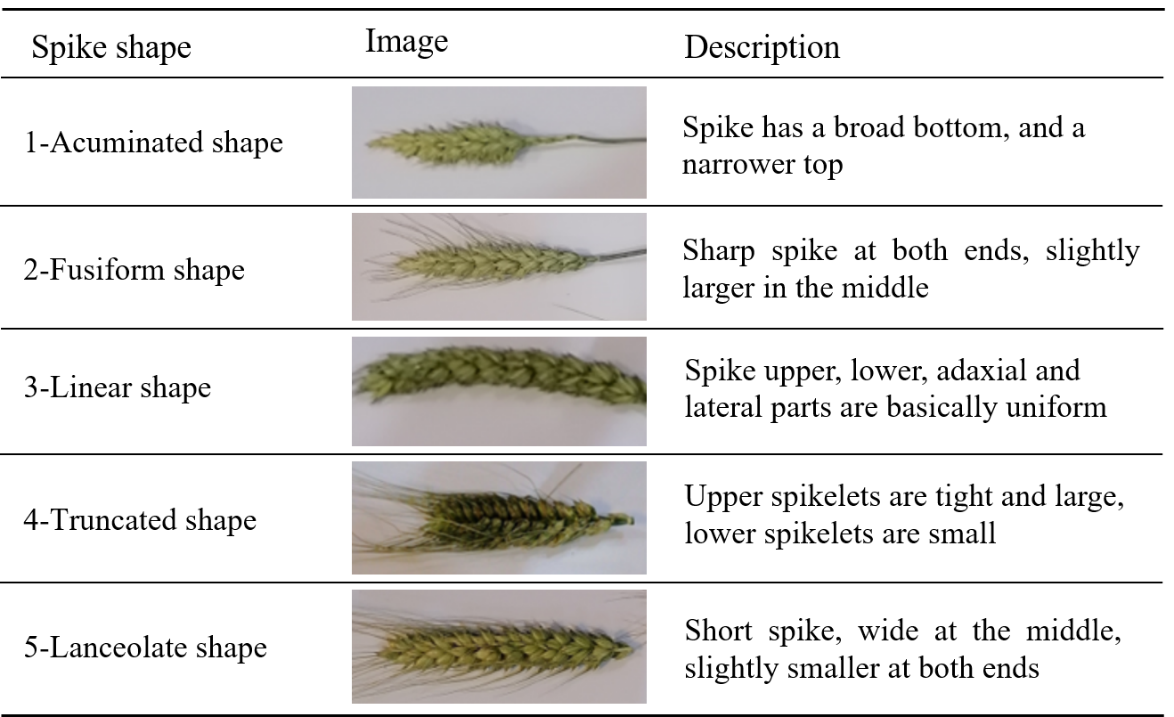


**Fig. S2.** The information on the image and description of different spike shapes.


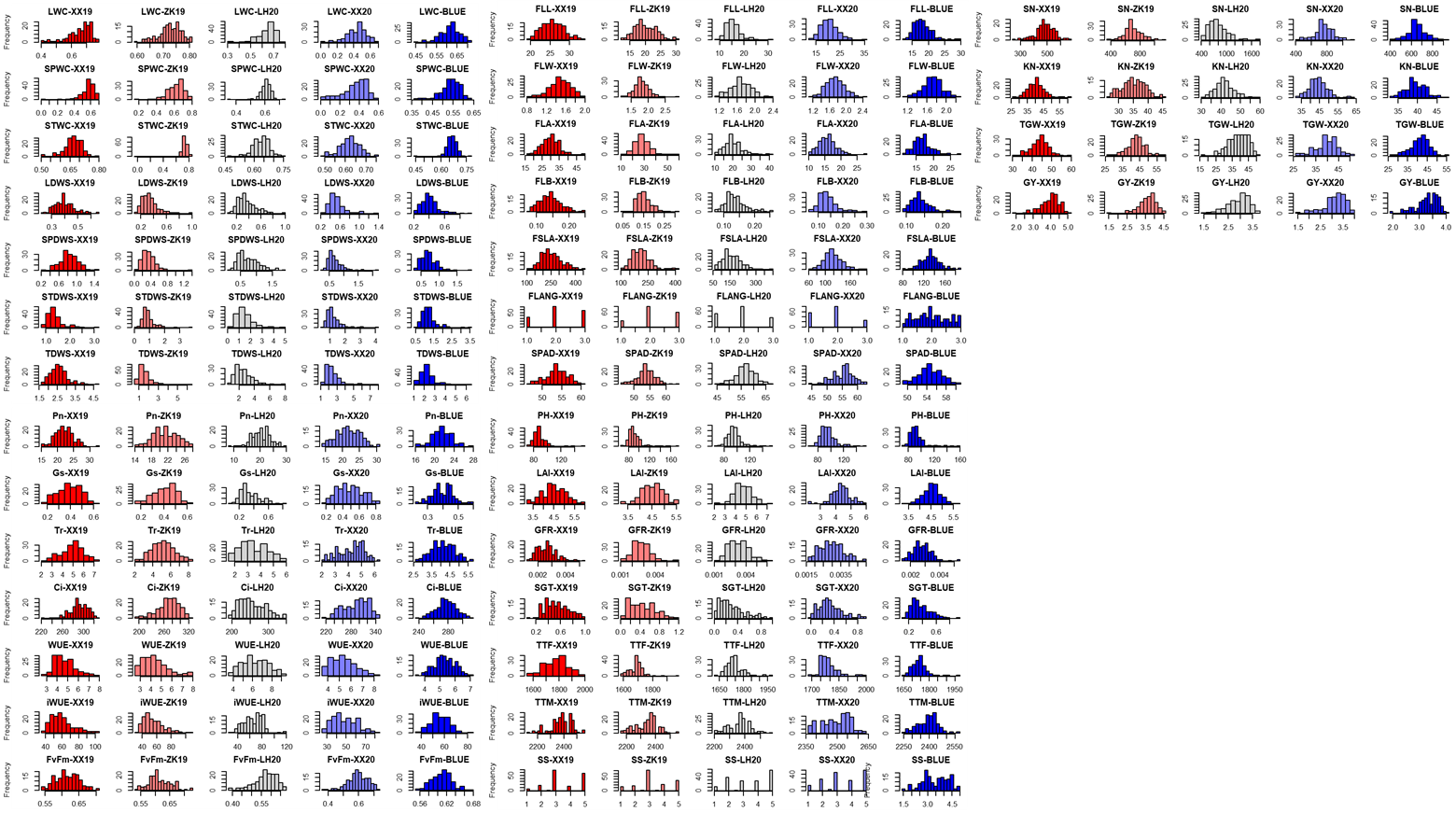


**Fig. S3.** Phenotypic variations for the 32 wheat traits measured under multiple environments.

BLUE, best linear unbiased estimation.


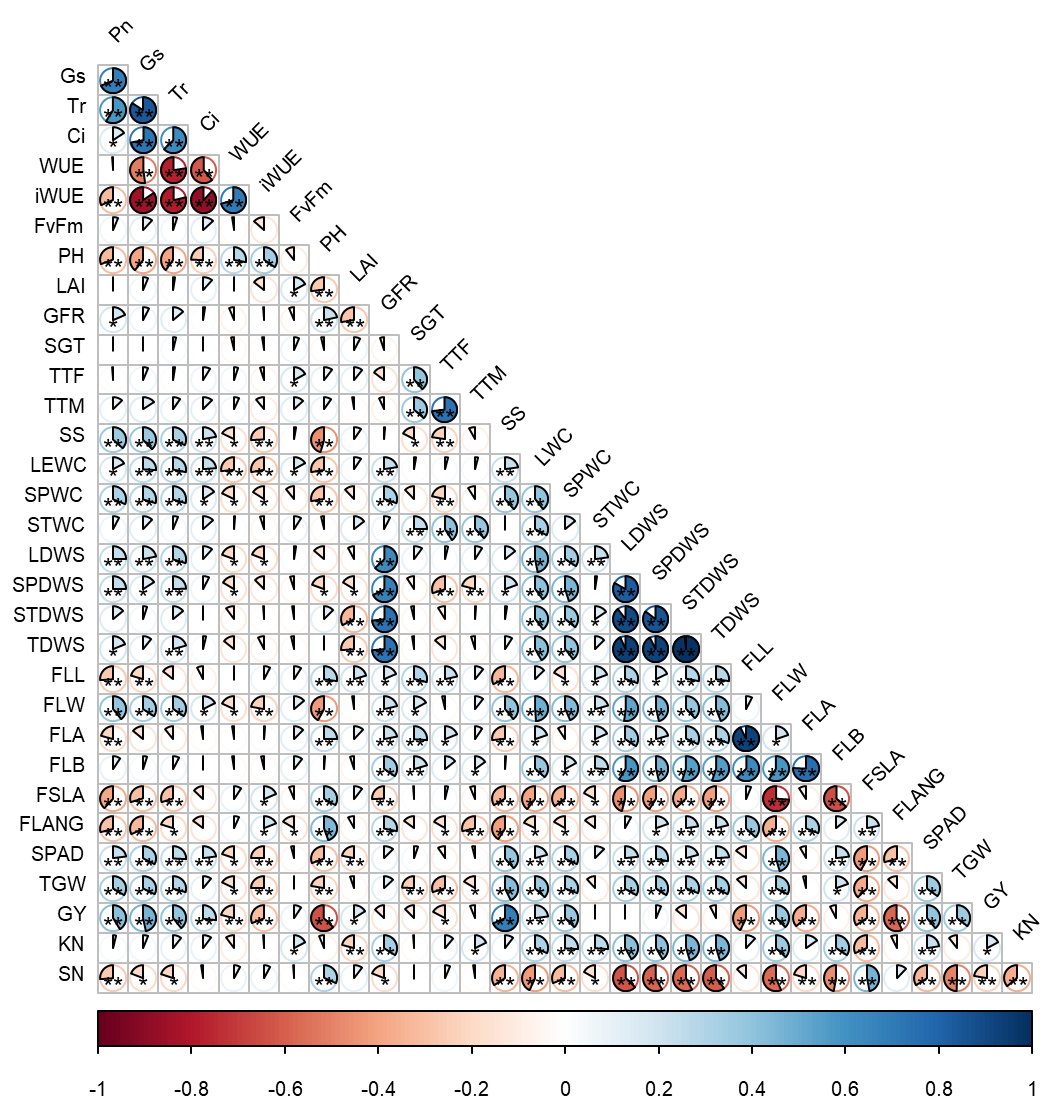


**Fig. S4.** Correlation of 32 wheat traits by best linear unbiased estimations for each trait across four environments.

Note: Pn, light-saturated net photosynthetic rate; Gs, stomatal conductance; Tr, transpiration rate; Ci, intercellular CO_2_ concentration; WUE, water use efficiency; iWUE, intrinsic water use efficiency; Fv’/Fm’, the maximum quantum yield of PSII photochemistry; PH, plant height; LAI, leaf area index; GFR, grain filling rate; SGT, stay green trait; TTF, thermal time from sowing to flowering stage; TTM, thermal time from sowing to maturity stage; SS, spike shape; LWC, leaf water content; SPWC, spike water content; STWC, stem water content; LDWS, leaf dry weights; SPDWS, spike dry weights; STDWS, stem dry weights; TDWS, total dry weights; FLL, flag leaf length; FLW, flag leaf width; FLA, flag leaf area; FLB, flag leaf biomass; FSLA, flag leaf specific leaf area; FLANG, flag leaf angle; SPAD, Chlorophyll content SPAD meter reading; KN, kernels number per spike; SN, spikes number per square meter; TGW, thousand-grain weight; GY, grain yield. The same below


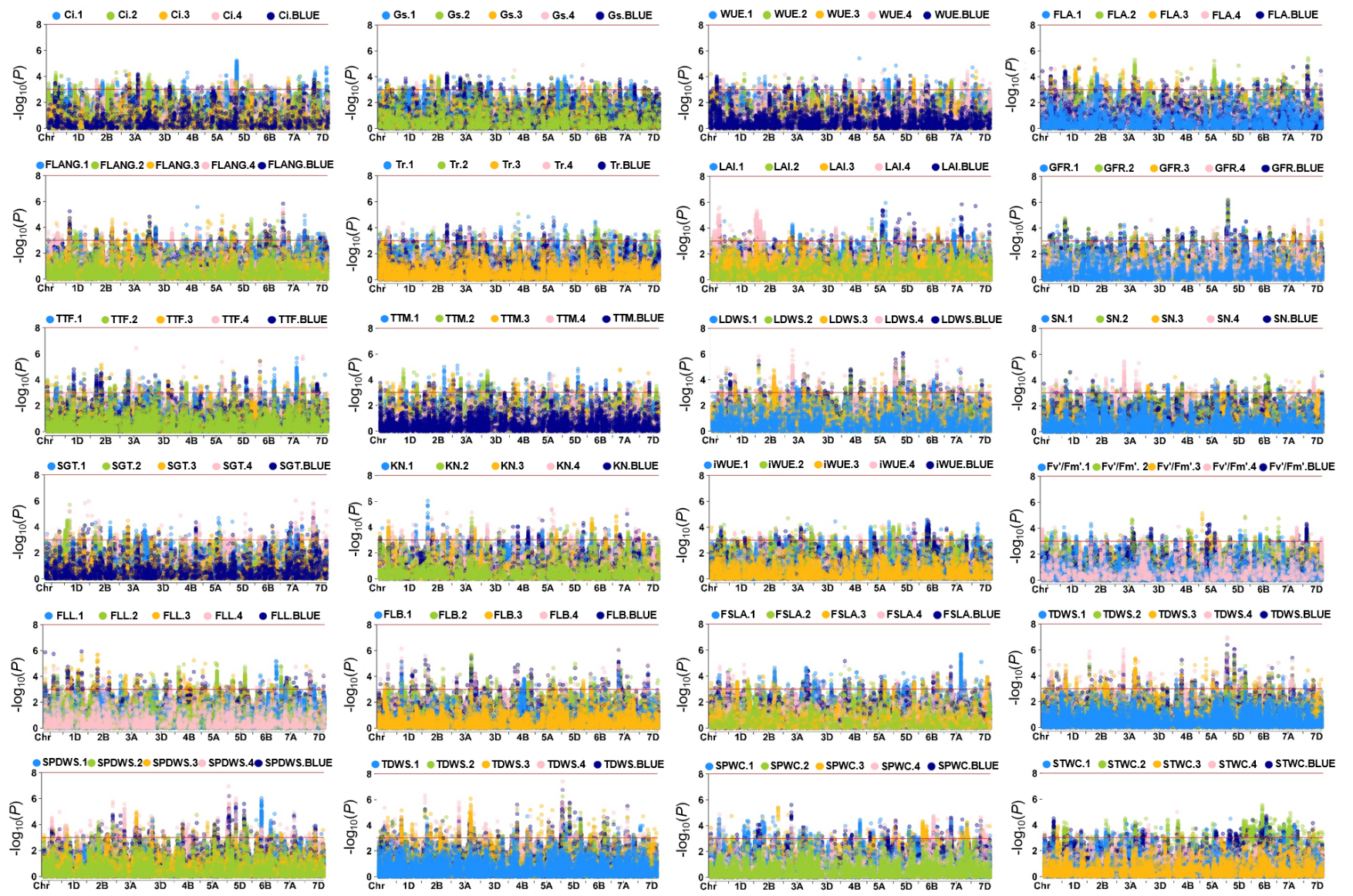


**Fig. S5.** Manhattan plots for a genome-wide association study of the traits in 166 wheat accessions under multiple environments.


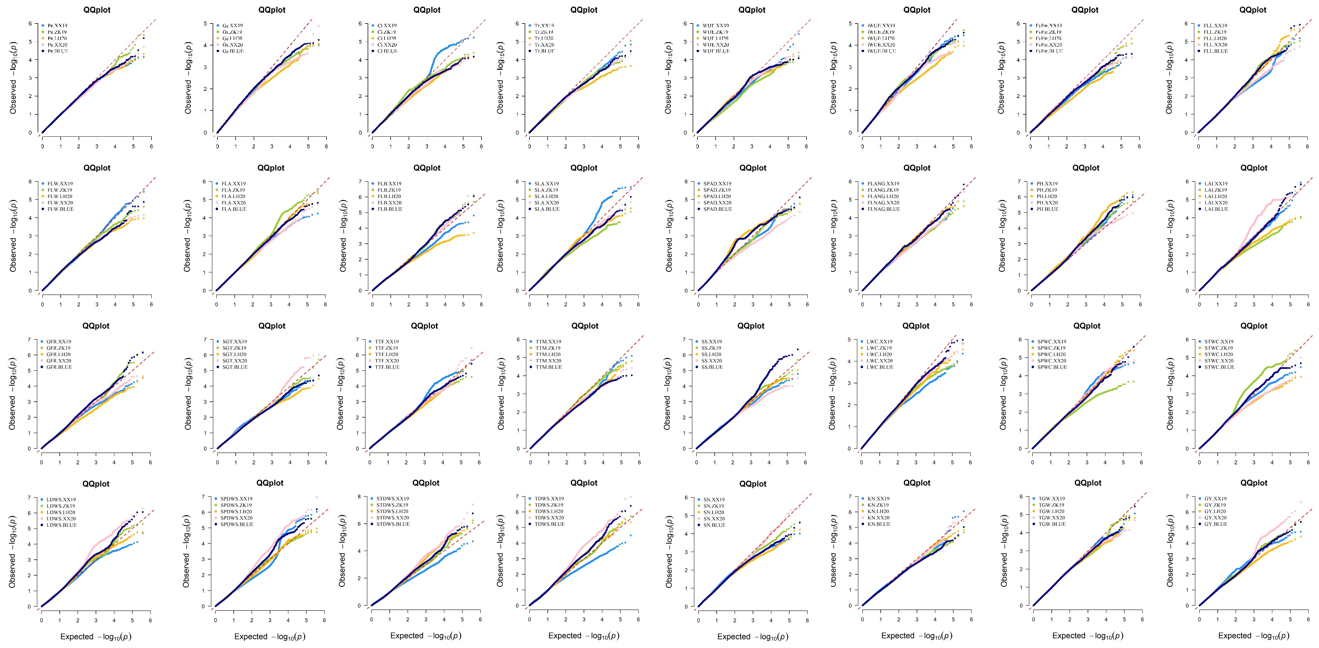
**Fig. S6.** Quantile - Quantile (Q-Q) plots of GWAS for 32 traits.


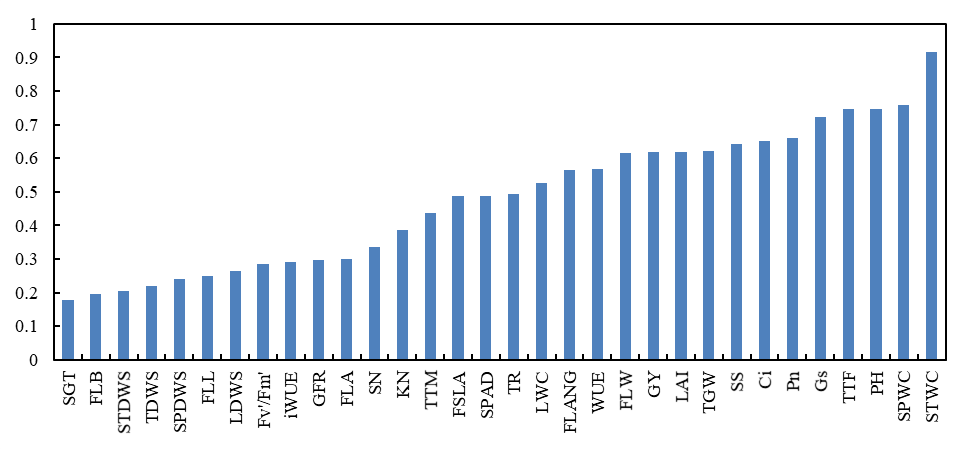


**Fig. S7.** Favorable allele frequencies of the identified QTLs.

**
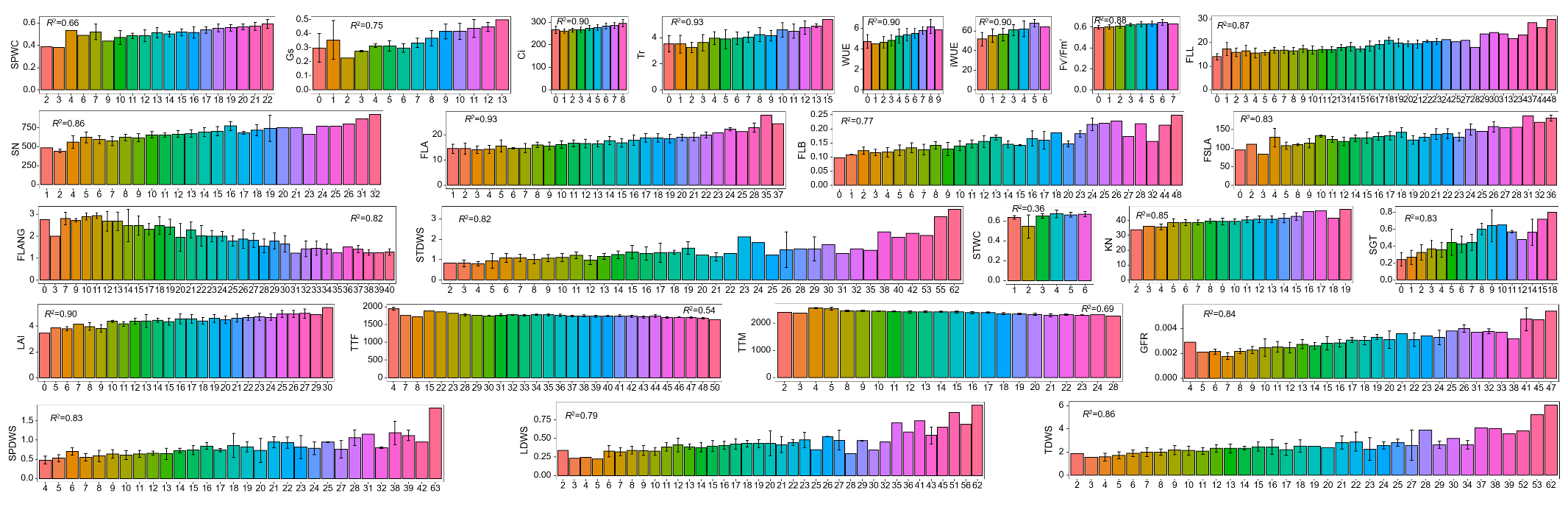
**

**Fig. S8.** Effect and distribution of favorable alleles of trait-associated markers.


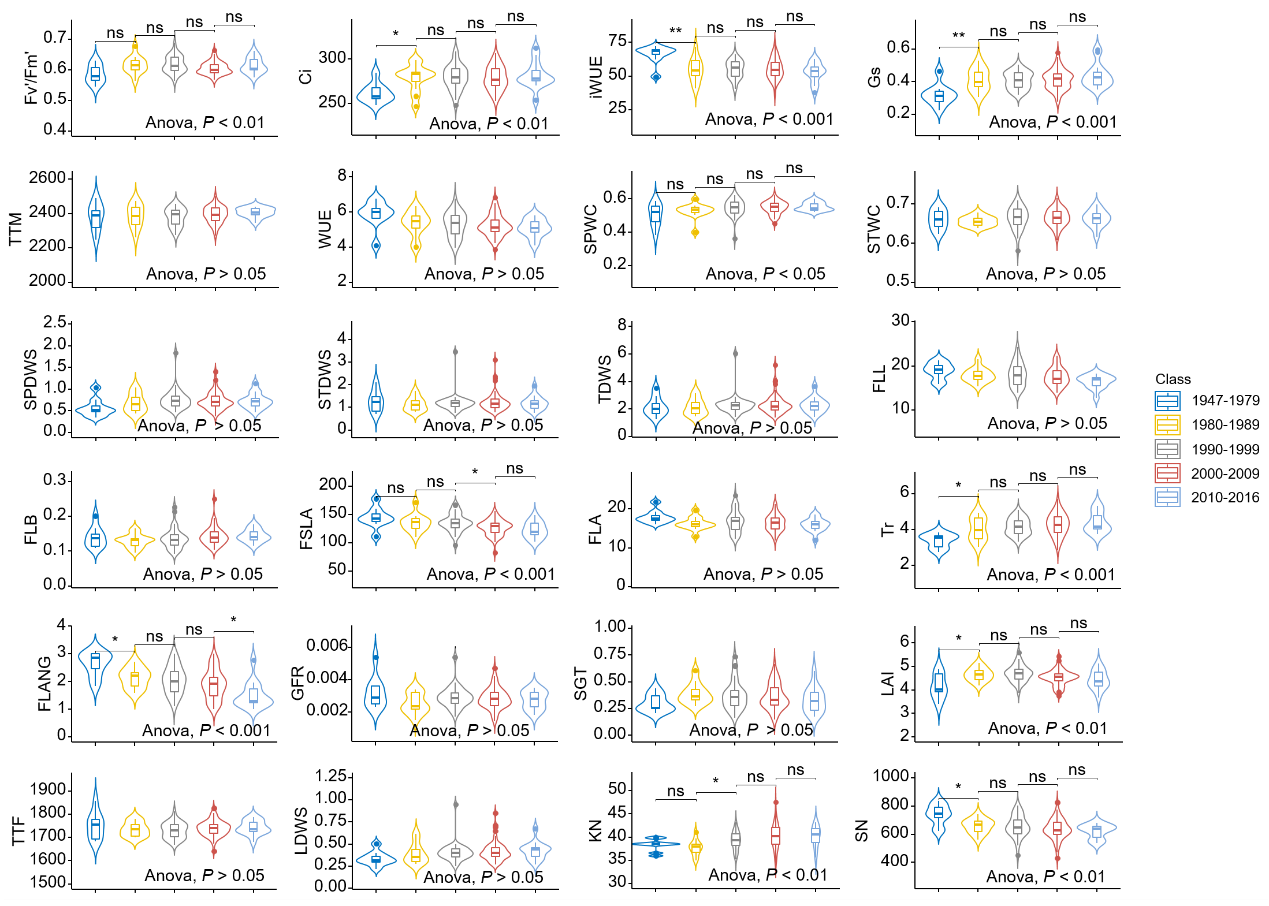


**Fig. S9.** Phenotypic changes in the traits values for 166 wheat cultivars released over the past 70 years (1947-2016).

Note: P1, P2, P3, P4 and P5 present 1947-1979, the 1980s, the 1990s, the 2000s and 2010-2016, respectively. ns, not significant; *, *P*<0.05; **, *P*<0.01; ***, *P*<0.001


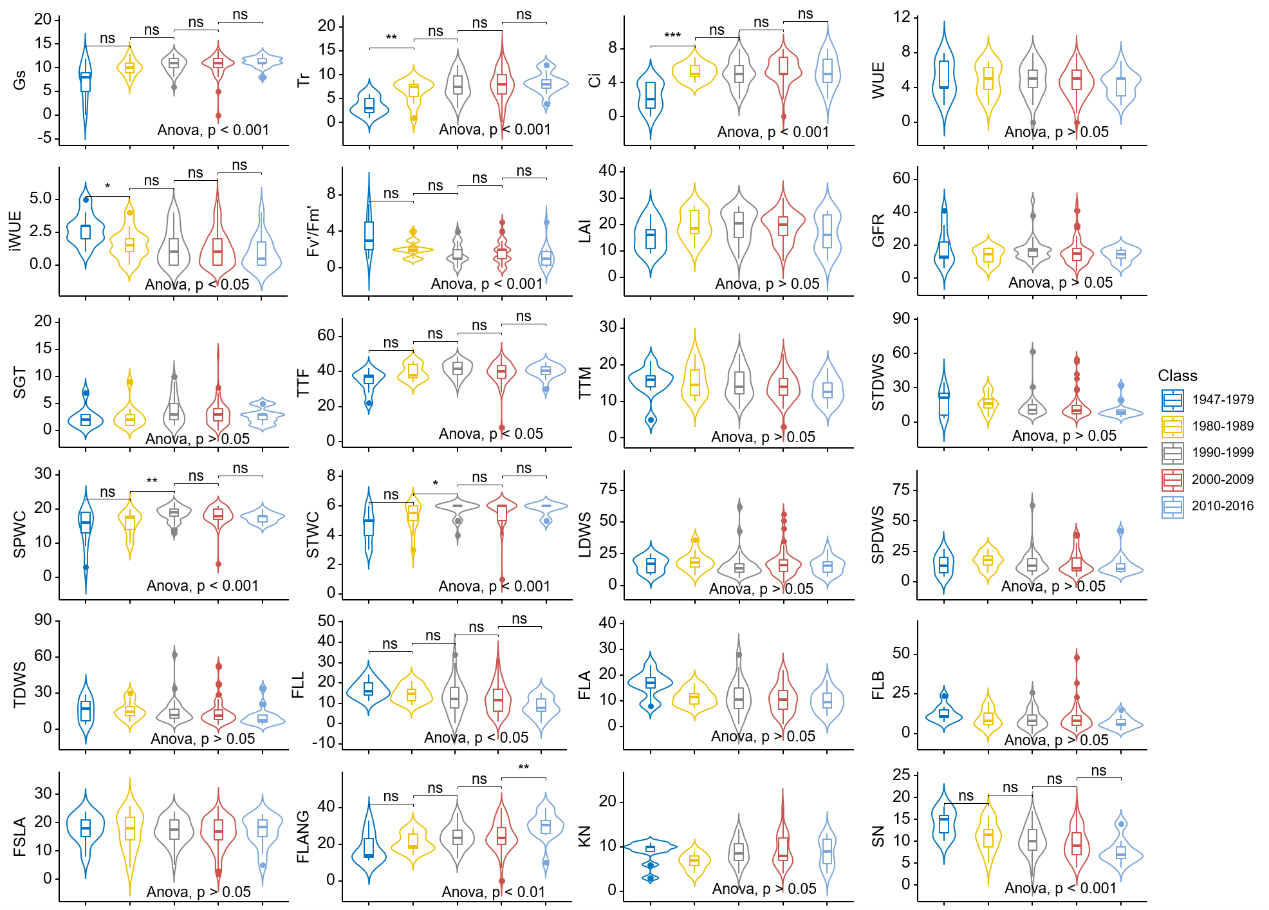


**Fig. S10.** Changes in numbers of increasing-effect alleles for 32 traits values over past 70 years (1947-2016).

Note: P1, P2, P3, P4 and P5 present 1947-1979, the 1980s, the 1990s, the 2000s and 2010-2016, respectively.

**Table S1**. Information of the 166 wheat accessions used in GWAS.

| ID | Variety | Origin | Released year | Population structure | Cluster by SOM |
| --- | --- | --- | --- | --- | --- |
| HH001 | Yumai47 | Henan | 1997 (P3) | 2 | 2 |
| HH002 | Bainong3217 | Henan | 1981 (P2) | 2 | 3 |
| HH003 | Shannong78-59 | Shaanxi | 2002 (P4) | 3 | 3 |
| HH004 | Zero-0 | Turkey | n.a. | 1 | 4 |
| HH005 | Jimai20 | Shandong | 2003 (P4) | 1 | 5 |
| HH006 | Sunong6 | Anhui | n.a. | 2 | 1 |
| HH007 | Zheng9023 | Henan | 2001 (P4) | 2 | 1 |
| HH008 | Huaimai18 | Jiangsu | 1999 (P3) | 1 | 2 |
| HH009 | Yumai50 | Henan | 1998 (P3) | 3 | 3 |
| HH010 | Yanzhan4110 | Henan | 2003 (P4) | 2 | 2 |
| HH011 | Xinong2000-7 | Shaanxi | 2006 (P4) | 2 | 2 |
| HH012 | Yumai34 | Henan | 1994 (P3) | 1 | 5 |
| HH013 | Xiaoyan22 | Shaanxi | 1998 (P3) | 3 | 2 |
| HH014 | Shixin828 | Hebei | 2013 (P5) | 2 | 2 |
| HH015 | Xinong291 | Shaanxi | n.a. | 1 | 5 |
| HH016 | Linhan2 | Shanxi | n.a. | 2 | 3 |
| HH017 | Neixiang188 | Henan | 2003 (P4) | 2 | 2 |
| HH018 | Wennong5 | Shandong | 2003 (P4) | 1 | 2 |
| HH019 | Gaoyou503 | Hebei | 1997 (P3) | 2 | 5 |
| HH020 | Shan253 | Shaanxi | 2001 (P4) | 2 | 3 |
| HH021 | Wanmai29 | Anhui | 1997 (P3) | 2 | 2 |
| HH022 | Wanmai19 | Anhui | 1994 (P3) | 2 | 5 |
| HH023 | Zhoumai26 | Henan | 2012 (P5) | 3 | 2 |
| HH024 | Huaimai20 | Jiangsu | 2003 (P4) | 1 | 5 |
| HH025 | Luyuan502 | Shandong | 2011 (P5) | 1 | 2 |
| HH026 | Zhoumai19 | Henan | 2004 (P4) | 2 | 2 |
| HH027 | Xinong979-005 | Shaanxi | 2005 (P4) | 2 | 5 |
| HH028 | Xinong1376 | Shaanxi | 1995 (P3) | 3 | 3 |
| HH029 | Huapei5 | Henan | 2006 (P4) | 3 | 5 |
| HH030 | Xinmai19 | Henan | 2006 (P4) | 2 | 2 |
| HH031 | Zhoumai23 | Henan | 2008 (P4) | 2 | 2 |
| HH032 | Lumai7 | Shandong | 1981 (P2) | 3 | 2 |
| HH033 | Xinmai9 | Henan | 1996 (P3) | 2 | 5 |
| HH034 | Norin67 | Japan | 1944 (P1) | 1 | 4 |
| HH035 | Zhongyu5 | Henan | 2001 (P4) | 3 | 3 |
| HH036 | Zhoumai12 | Henan | 1999 (P3) | 3 | 3 |
| HH037 | Zhoumai25 | Henan | 2010 (P5) | 3 | 2 |
| HH038 | Dorico | Italy | 1993 (P3) | 1 | 2 |
| HH039 | Kitanokaori | Japan | 2003 (P4) | 3 | 4 |
| HH040 | Yumai2 | Henan | 1983 (P2) | 3 | 5 |
| HH041 | Lumai8 | Shandong | 1985 (P2) | 1 | 2 |
| HH042 | Taishan5 | Shandong | 1983 (P2) | 1 | 5 |
| HH043 | Bainong64 | Henan | 2000 (P4) | 2 | 5 |
| HH044 | Zhoumai28 | Henan | 2013 (P5) | 3 | 2 |
| HH045 | Mantol | Italy | n.a. | 1 | 5 |
| HH046 | 85Zhong33 | Henan | 1990 (P3) | 3 | 5 |
| HH047 | Jimai21 | Shandong | 2004 (P4) | 1 | 2 |
| HH048 | Shi4185 | Hebei | 1999 (P3) | 3 | 5 |
| HH049 | Zhoumai18 | Henan | 2004 (P4) | 3 | 2 |
| HH050 | Heng7228 | Hebei | 2005 (P4) | 3 | 2 |
| HH051 | Luohan2 | Henan | 2001 (P4) | 2 | 5 |
| HH052 | Barra | Italy | n.a. | 1 | 5 |
| HH053 | Zhoumai32 | Henan | 2014 (P5) | 3 | 2 |
| HH054 | Abbondanza | Italy | 1950 (P1) | 1 | 3 |
| HH055 | Luomai21 | Henan | 2009 (P4) | 3 | 2 |
| HH056 | Shan354 | Shaanxi | 1998 (P3) | 3 | 5 |
| HH057 | Han6172 | Hebei | 2001 (P4) | 3 | 2 |
| HH058 | Aca801 | Argentina | 2002 (P4) | 1 | 5 |
| HH059 | Jinan13 | Shandong | 1980 (P2) | 1 | 5 |
| HH060 | Yumai21 | Henan | 1992 (P3) | 1 | 5 |
| HH061 | Libero | Italy | 1993 (P3) | 1 | 5 |
| HH062 | Zixuan2 | Shandong | 1970 (P1) | 1 | 5 |
| HH063 | Xinong88 | Shaanxi | 1995 (P3) | 2 | 2 |
| HH064 | Huaimai21 | Jiangsu | 2006 (P4) | 3 | 2 |
| HH065 | Zhoumai30 | Henan | 2016 (P5) | 3 | 1 |
| HH066 | Sagittario | Italy | 1994 (P3) | 1 | 5 |
| HH067 | Aifeng3 | Shaanxi | 1971 (P1) | 1 | 4 |
| HH068 | Yannong19 | Shandong | 2001 (P4) | 1 | 2 |
| HH069 | Yannong18 | Shandong | 1999 (P3) | 3 | 5 |
| HH070 | Yumai35 | Henan | 1995 (P3) | 3 | 3 |
| HH071 | Zhoumai22 | Henan | 2007 (P4) | 3 | 2 |
| HH072 | Linkang12 | Shanxi | n.a. | 3 | 5 |
| HH073 | Luami15 | Shandong | 1992 (P3) | 3 | 2 |
| HH074 | Hengguan33 | Hebei | n.a. | 3 | 2 |
| HH075 | ProINTAColibr1 | Argentina | 1998 (P3) | 1 | 4 |
| HH076 | Xiaoyan81 | Shaanxi | 2005 (P4) | 2 | 5 |
| HH077 | Xinmai9408 | Henan | 2003 (P4) | 2 | 3 |
| HH078 | Shannong981 | Shaanxi | n.a. | 2 | 3 |
| HH079 | Jimai22 | Shandong | 2006 (P4) | 1 | 2 |
| HH080 | Lumai14 | Shandong | 1990 (P3) | 1 | 5 |
| HH081 | Zhoumai13 | Henan | 1999 (P3) | 3 | 2 |
| HH082 | Yumai49 | Henan | 2000 (P4) | 2 | 2 |
| HH083 | Aca601 | Argentina | 2002 (P4) | 1 | 5 |
| HH084 | An1331 | Anhui | n.a. | 2 | 5 |
| HH085 | Lumai11 | Shandong | 1988 (P2) | 1 | 5 |
| HH086 | Shan512 | Shaanxi | 2004 (P4) | 2 | 5 |
| HH087 | Kanto107 | Japan | 1995 (P3) | 1 | 3 |
| HH088 | Shanmai509 | Shaanxi | n.a. | 3 | 5 |
| HH089 | Lumai21 | Shandong | 1996 (P3) | 1 | 5 |
| HH090 | NideraBaguette20 | Argentina | n.a. | 1 | 4 |
| HH091 | Shanyou225 | Shaanxi | 1992 (P3) | 2 | 2 |
| HH092 | Jinmai61 | Shanxi | 1999 (P3) | 1 | 5 |
| HH093 | Zhou8425B | Henan | 1988 (P2) | 3 | 1 |
| HH094 | Genio | Italy | 1992 (P3) | 1 | 5 |
| HH095 | KleinJabal1 | Argentina | 2003 (P4) | 1 | 4 |
| HH096 | Shijiazhuang8 | Hebei | 2001 (P4) | 3 | 5 |
| HH097 | Zhoumai11 | Henan | 1997 (P3) | 3 | 2 |
| HH098 | Su0663 | Anhui | n.a. | 2 | 2 |
| HH099 | Jishi02-1 | Hebei | 2004 (P4) | 2 | 5 |
| HH100 | Fu936 | Anhui | 1993 (P3) | 2 | 5 |
| HH101 | Zhengmai366 | Henan | 2005 (P4) | 2 | 2 |
| HH102 | Zimai12 | Shandong | 2001 (P4) | 1 | 1 |
| HH103 | Norin61 | Japan | 1944 (P1) | 1 | 4 |
| HH104 | Lumai9 | Shandong | 1980 (P2) | 1 | 5 |
| HH105 | Lumai23 | Shandong | 1996 (P3) | 1 | 1 |
| HH106 | Shijiazhuang15 | Hebei | 2005 (P4) | 3 | 5 |
| HH107 | Wanmai52 | Anhui | 2004 (P4) | 2 | 5 |
| HH108 | Yumai63 | Henan | 2003 (P4) | 2 | 5 |
| HH109 | Yumai18 | Henan | 1990 (P3) | 2 | 2 |
| HH110 | Jimai19 | Shandong | 2001 (P4) | 1 | 2 |
| HH111 | Liangxing66 | Shandong | 2008 (P4) | 1 | 5 |
| HH112 | Shan229 | Shaanxi | 1993 (P3) | 2 | 5 |
| HH113 | Taishan1 | Shandong | 1979 (P1) | 1 | 4 |
| HH114 | Wunong148 | Shaanxi | 2000 (P4) | 2 | 3 |
| HH115 | Jinan17 | Shandong | 1993 (P3) | 1 | 5 |
| HH116 | Fengchan3 | Shaanxi | 1966 (P1) | 2 | 3 |
| HH117 | Guan35 | Hebei | 2004 (P4) | 3 | 2 |
| HH118 | Wennong14 | Shandong | 2010 (P5) | 1 | 5 |
| HH119 | Linmai2 | Shandong | 2004 (P4) | 1 | 1 |
| HH120 | Aikang58 | Henan | 2005 (P4) | 3 | 2 |
| HH121 | Liangxing99 | Shandong | 2004 (P4) | 1 | 2 |
| HH122 | Wanmai38 | Anhui | 1997 (P3) | 1 | 2 |
| HH123 | Wanmai50 | Anhui | 2004 (P4) | 2 | 2 |
| HH124 | Lankao906 | Henan | 2001 (P4) | 3 | 1 |
| HH125 | Lampo | Italy | 1994 (P3) | 1 | 5 |
| HH126 | Yumai57 | Henan | 1999 (P3) | 2 | 3 |
| HH127 | Jinhe9123 | Hebei | 2008 (P4) | 3 | 2 |
| HH128 | Lumai6 | Shandong | 1980 (P2) | 2 | 5 |
| HH129 | Xiaoyan54 | Shaanxi | 2000 (P4) | 2 | 5 |
| HH130 | Zhoumai31 | Henan | 2010 (P5) | 2 | 2 |
| HH131 | Zhoumai16 | Henan | 2002 (P4) | 3 | 2 |
| HH132 | Wanmai53 | Anhui | 2004 (P4) | 2 | 2 |
| HH133 | Xiaoyan6 | Shaanxi | 1981 (P2) | 2 | 5 |
| HH134 | Shixin733 | Hebei | 2001 (P4) | 2 | 2 |
| HH135 | Zhongmai875 | Henan | 2014 (P5) | 3 | 2 |
| HH136 | Gaocheng8901 | Hebei | 1998 (P3) | 1 | 5 |
| HH137 | Sunstate | Australia | 1993 (P3) | 1 | 3 |
| HH138 | Zhong892 | Henan | n.a. | 3 | 3 |
| HH139 | Shiyou17 | Hebei | 2007 (P4) | 1 | 5 |
| HH140 | Shannong20 | Shandong | 2010 (P5) | 1 | 2 |
| HH141 | Zhongmai871 | Henan | 2012 (P5) | 3 | 2 |
| HH142 | Neixiang5 | Henan | 1955 (P1) | 3 | 4 |
| HH143 | Funo | Italy | 1944 (P1) | 2 | 3 |
| HH144 | Zhengzhou3 | Henan | 1965 (P1) | 2 | 4 |
| HH145 | Zhengyin1 | Henan | 1965 (P1) | 2 | 3 |
| HH146 | Yumai7 | Henan | 1982 (P2) | 3 | 2 |
| HH147 | Yumai13 | Henan | 1991 (P3) | 1 | 2 |
| HH148 | Bima1 | Shaanxi | 1947 (P1) | 1 | 4 |
| HH149 | Bima4 | Shaanxi | 1947 (P1) | 1 | 4 |
| HH150 | Shan715 | Shaanxi | 2005 (P4) | 3 | 5 |
| HH151 | Linmai4 | Shandong | 2006 (P4) | 1 | 1 |
| HH152 | Zhongyu9 | Henan | 2004 (P4) | 3 | 2 |
| HH153 | PH82-2 | Shandong | 1991 (P3) | 2 | 5 |
| HH154 | Lankao241 | Henan | n.a. | 3 | 1 |
| HH155 | Wan23094 | Anhui | 2007 (P4) | 2 | 2 |
| HH156 | Jining16 | Shandong | 2004 (P4) | 1 | 1 |
| HH157 | Lumai5 | Shandong | 1984 (P2) | 1 | 2 |
| HH158 | Wanmai33 | Anhui | 1997 (P3) | 2 | 1 |
| HH159 | NideraBaguette10 | Argentina | n.a. | 1 | 4 |
| HH160 | Yannong15 | Shandong | 1976 (P1) | 1 | 5 |
| HH161 | KleinFlecha | Argentina | 2003 (P4) | 1 | 4 |
| HH162 | Shanmai94 | Shaanxi | 2006 (P4) | 2 | 5 |
| HH163 | 11CA40 | Henan | 2011 (P5) | 1 | 2 |
| HH164 | Lankao242 | Henan | n.a. | 3 | 1 |
| HH165 | Shan150 | Shaanxi | 2000 (P4) | 2 | 5 |
| HH166 | Zhongmai895 | Henan | 2012 (P5) | 3 | 2 |

**Table S2**. Loci for 32 traits identified by GWAS.

| Trait | ENV* | SNP | Chromosome | Allele | Physical positions* | P.value | PVG |
| --- | --- | --- | --- | --- | --- | --- | --- |
| FLL | 2 3 4 B | AX_109621606 | 1A | C/T | 9.65-10.16 | 1.37E-06 | 0.14 |
| FLL | 3 4 B | AX_109287750 | 1A | C/T | 504.41-516.57 | 1.83E-06 | 0.14 |
| FLL | 3 B | AX_95103899 | 1A | G/C | 530.23-530.23 | 1.32E-04 | 0.08 |
| FLL | 1 2 | AX_109954861 | 1B | A/G | 9.58-9.58 | 2.64E-04 | 0.07 |
| FLL | 4 B | AX_110395082 | 1B | C/A | 21.51-21.51 | 3.05E-04 | 0.07 |
| FLL | 3 B | AX_94614751 | 1B | C/G | 573.57-573.57 | 3.51E-05 | 0.10 |
| FLL | 3 4 B | AX_109287909 | 1B | C/G | 643.18-645.14 | 1.80E-05 | 0.11 |
| FLL | 3 B | AX_111630180 | 1D | C/G | 8.01-11.52 | 1.52E-04 | 0.08 |
| FLL | 3 B | AX_109881112 | 1D | C/A | 48.45-49.74 | 1.90E-04 | 0.08 |
| FLL | 3 B | AX_94853121 | 1D | T/C | 423.04-432.71 | 4.85E-05 | 0.10 |
| FLL | 3 4 B | AX_109407628 | 2A | A/G | 7.80-7.80 | 1.17E-06 | 0.14 |
| FLL | 3 B | AX_111137210 | 2A | T/C | 29.87-32.53 | 5.39E-05 | 0.09 |
| FLL | 4 B | AX_108756384 | 2A | G/C | 711.97-711.97 | 6.49E-04 | 0.07 |
| FLL | 3 B | AX_110044278 | 2A | T/C | 731.25-732.58 | 6.24E-04 | 0.07 |
| FLL | 3 B | AX_109930261 | 2B | C/G | 29.14-29.15 | 1.33E-04 | 0.08 |
| FLL | 3 B | AX_111634394 | 2B | C/T | 49.65-50.55 | 2.54E-05 | 0.10 |
| FLL | 3 B | AX_95652945 | 2B | G/A | 65.10-65.10 | 1.13E-04 | 0.09 |
| FLL | 3 B | AX_89345252 | 2B | T/G | 744.18-753.97 | 3.90E-04 | 0.07 |
| FLL | 4 B | AX_110558769 | 3A | A/G | 580.42-580.42 | 5.73E-04 | 0.07 |
| FLL | 4 B | AX_109838665 | 3A | T/G | 644.81-645.76 | 7.38E-04 | 0.07 |
| FLL | 3 B | AX_109431705 | 3A | G/A | 721.66-724.70 | 2.07E-04 | 0.08 |
| FLL | 3 B | AX_109958385 | 3B | C/A | 42.47-42.47 | 8.46E-05 | 0.09 |
| FLL | 3 B | AX_94509137 | 3B | A/G | 651.74-651.74 | 9.99E-04 | 0.06 |
| FLL | 3 B | AX_94698727 | 3B | A/G | 813.95-813.95 | 5.56E-04 | 0.07 |
| FLL | 4 B | AX_94922328 | 3D | T/C | 508.39-508.39 | 7.83E-04 | 0.06 |
| FLL | 3 B | AX_111068370 | 3D | G/C | 604.69-606.61 | 6.93E-05 | 0.09 |
| FLL | 4 B | AX_110077614 | 4A | T/C | 24.90-24.90 | 7.12E-04 | 0.07 |
| FLL | 3 B | AX_111629578 | 4A | T/C | 590.43-590.43 | 6.07E-05 | 0.09 |
| FLL | 3 B | AX_109498050 | 4A | A/T | 664.17-664.34 | 6.58E-04 | 0.07 |
| FLL | 3 B | AX_111164343 | 4A | A/G | 701.99-701.99 | 1.94E-04 | 0.08 |
| FLL | 3 B | AX_108856162 | 4B | G/A | 196.78-196.78 | 9.01E-04 | 0.06 |
| FLL | 3 B | AX_109520590 | 4B | G/A | 292.53-292.53 | 6.77E-04 | 0.07 |
| FLL | 3 B | AX_109896021 | 4B | G/A | 323.34-323.34 | 6.92E-04 | 0.07 |
| FLL | 3 B | AX_111476370 | 4B | G/A | 397.05-397.05 | 9.42E-04 | 0.06 |
| FLL | 4 B | D_contig26957_307 | 4B | A/C | 409.74-409.74 | 1.70E-04 | 0.08 |
| FLL | 3 B | AX_94701329 | 4B | C/T | 629.25-629.25 | 4.48E-05 | 0.10 |
| FLL | 3 B | AX_110528269 | 4D | T/C | 312.59-312.59 | 3.88E-04 | 0.07 |
| FLL | 3 B | AX_111731847 | 4D | C/G | 347.52-358.48 | 1.09E-04 | 0.09 |
| FLL | 3 B | AX_111480164 | 4D | A/G | 498.01-509.42 | 3.34E-04 | 0.07 |
| FLL | 2 B | AX_109532277 | 5A | C/T | 698.37-699.47 | 7.72E-04 | 0.06 |
| FLL | 3 B | AX_110656433 | 5B | A/C | 278.00-278.00 | 6.37E-04 | 0.07 |
| FLL | 3 B | AX_94622383 | 5B | A/G | 683.14-684.58 | 2.84E-04 | 0.08 |
| FLL | 4 B | AX_111137392 | 5D | G/C | 266.33-266.33 | 3.32E-04 | 0.07 |
| FLL | 3 B | AX_94388289 | 5D | C/G | 319.22-319.22 | 9.88E-04 | 0.06 |
| FLL | 3 B | AX_109992302 | 5D | G/A | 403.68-403.68 | 6.17E-04 | 0.07 |
| FLL | 2 3 B | AX_111597613 | 5D | C/A | 542.68-545.12 | 9.73E-05 | 0.09 |
| FLL | 3 B | AX_86176492 | 6D | G/A | 2.41-2.41 | 9.70E-04 | 0.06 |
| FLL | 1 2 | AX_109202589 | 6D | T/G | 392.03-392.03 | 1.44E-04 | 0.08 |
| FLL | 1 2 | AX_109955189 | 6D | A/G | 400.72-405.49 | 1.11E-04 | 0.08 |
| FLL | 3 B | AX_108915587 | 7A | C/T | 115.32-116.62 | 1.27E-04 | 0.08 |
| FLL | 4 B | Kukri_c717_98 | 7A | G/A | 227.09-227.09 | 1.46E-04 | 0.08 |
| FLL | 3 B | AX_111087383 | 7A | G/C | 251.84-251.84 | 1.53E-04 | 0.08 |
| FLL | 2 B | AX_111048466 | 7A | C/A | 545.74-545.74 | 3.04E-04 | 0.08 |
| FLL | 4 B | AX_94603127 | 7A | A/G | 672.03-672.03 | 1.30E-04 | 0.08 |
| FLL | 3 B | AX_109920673 | 7B | A/T | 428.48-428.48 | 6.57E-05 | 0.09 |
| FLL | 4 B | AX_86162674 | 7B | C/G | 647.76-648.38 | 8.42E-05 | 0.09 |
| FLL | 2 4 B | wsnp_Ex_c5268_9320618 | 7B | T/C | 701.42-702.32 | 2.43E-04 | 0.08 |
| FLL | 4 B | AX_94788487 | 7D | T/G | 278.34-278.34 | 9.20E-04 | 0.06 |
| FLL | 4 B | AX_94749119 | 7D | A/C | 580.57-580.57 | 9.29E-05 | 0.09 |
| FLW | 2 3 4 B | AX_95104798 | 1A | A/G | 534.05-551.98 | 1.34E-05 | 0.11 |
| FLW | 1 3 | AX_111800689 | 1B | G/T | 40.36-40.39 | 6.22E-04 | 0.07 |
| FLW | 1 3 | AX_94919611 | 1B | C/T | 321.24-321.24 | 6.15E-04 | 0.07 |
| FLW | 3 4 B | AX_111510499 | 1B | A/C | 341.36-343.88 | 1.39E-04 | 0.09 |
| FLW | 2 3 4 B | AX_94541532 | 1B | G/A | 631.71-633.85 | 6.14E-05 | 0.10 |
| FLW | 1 2 | AX_109861852 | 1D | A/G | 26.41-27.86 | 1.21E-05 | 0.12 |
| FLW | 2 3 4 B | AX_94823257 | 1D | C/G | 460.74-460.86 | 2.36E-05 | 0.11 |
| FLW | 4 B | AX_111545497 | 2A | T/C | 754.46-754.47 | 5.53E-04 | 0.07 |
| FLW | 3 4 B | AX_110395854 | 2B | T/G | 572.32-572.32 | 1.39E-04 | 0.09 |
| FLW | 3 B | wsnp_BF473744B_Ta_2_2 | 2B | C/A | 648.08-648.08 | 2.91E-04 | 0.08 |
| FLW | 3 B | Tdurum_contig59522_262 | 2B | G/A | 665.19-665.19 | 3.95E-04 | 0.07 |
| FLW | 3 B | RFL_Contig4403_1034 | 3A | A/C | 176.56-176.56 | 4.29E-04 | 0.07 |
| FLW | 1 4 | AX_111493015 | 3A | A/G | 268.33-268.33 | 7.78E-04 | 0.07 |
| FLW | 1 B | AX_108952371 | 3A | C/T | 346.96-346.96 | 6.22E-04 | 0.07 |
| FLW | 2 4 | AX_108833035 | 3A | C/A | 731.43-731.43 | 8.92E-04 | 0.07 |
| FLW | 4 B | AX_111655083 | 3B | G/C | 24.42-24.43 | 6.80E-04 | 0.07 |
| FLW | 1 4 | AX_108896845 | 3B | C/T | 40.53-40.53 | 2.41E-05 | 0.11 |
| FLW | 1 3 | AX_109288262 | 3B | T/C | 810.54-810.54 | 2.90E-04 | 0.08 |
| FLW | 4 B | Kukri_c20012_1362 | 4A | G/A | 628.33-628.33 | 9.88E-04 | 0.06 |
| FLW | 4 B | RAC875_c48283_1574 | 4D | A/G | 134.13-134.13 | 2.96E-04 | 0.08 |
| FLW | 1 3 | AX_111100639 | 5B | G/C | 485.45-485.45 | 2.63E-04 | 0.08 |
| FLW | 3 B | RAC875_c10394_828 | 5B | G/A | 528.71-533.51 | 2.25E-04 | 0.08 |
| FLW | 3 B | AX_111497976 | 5B | C/T | 557.35-557.35 | 4.07E-04 | 0.07 |
| FLW | 3 B | AX_109309727 | 5B | T/C | 572.34-572.34 | 3.57E-04 | 0.08 |
| FLW | 3 B | AX_110984791 | 5B | C/T | 576.57-577.30 | 3.08E-04 | 0.08 |
| FLW | 4 B | BS00024814_51 | 5B | A/C | 671.30-671.30 | 3.67E-04 | 0.07 |
| FLW | 4 B | RAC875_c16354_606 | 5D | G/A | 295.58-302.39 | 3.70E-04 | 0.07 |
| FLW | 2 4 B | AX_112289177 | 6A | T/G | 112.59-112.59 | 6.43E-04 | 0.07 |
| FLW | 2 B | AX_109473736 | 6A | A/C | 615.14-615.14 | 2.92E-04 | 0.08 |
| FLW | 4 B | AX_110386434 | 6B | G/C | 22.38-22.39 | 9.52E-04 | 0.06 |
| FLW | 4 B | AX_111481734 | 6B | G/C | 581.94-581.94 | 4.50E-04 | 0.07 |
| FLW | 1 4 B | AX_111476049 | 6B | C/T | 594-599.88 | 2.74E-04 | 0.08 |
| FLW | 1 4 B | AX_109308849 | 6B | T/C | 614.31-618.94 | 9.73E-04 | 0.06 |
| FLW | 4 B | Tdurum_contig52015_1090 | 7A | A/G | 3.85-3.91 | 1.48E-04 | 0.09 |
| FLW | 2 4 B | AX_109404331 | 7A | G/C | 33.51-33.54 | 5.50E-04 | 0.07 |
| FLW | 4 B | AX_111569442 | 7A | C/G | 116.19-116.50 | 2.60E-04 | 0.08 |
| FLW | 4 B | Ra_c105310_668 | 7A | A/G | 679.84-680.33 | 3.35E-04 | 0.08 |
| FLW | 4 B | AX_110773195 | 7D | A/G | 129.28-129.28 | 7.74E-04 | 0.07 |
| FLW | 2 4 B | AX_109147695 | 7D | T/C | 217.93-224.34 | 6.64E-04 | 0.07 |
| FLW | 2 B | AX_110227474 | 7D | C/T | 255.23-258.59 | 5.56E-04 | 0.07 |
| FLW | 2 B | AX_109231044 | 7D | T/C | 345.81-345.81 | 8.31E-04 | 0.07 |
| FLW | 3 B | AX_111032450 | 7D | T/C | 399.43-412.01 | 2.71E-04 | 0.08 |
| FLA | 4 B | AX_109621606 | 1A | C/T | 9.65-10.05 | 3.66E-05 | 0.10 |
| FLA | 4 B | AX_109287750 | 1A | C/T | 504.25-516.57 | 2.38E-05 | 0.11 |
| FLA | 4 B | AX_111501644 | 1B | C/T | 20.72-26.86 | 3.14E-04 | 0.08 |
| FLA | 3 B | AX_94614751 | 1B | C/G | 573.57-573.57 | 1.21E-04 | 0.09 |
| FLA | 4 B | AX_111626571 | 1B | G/C | 643.46-645.14 | 3.63E-05 | 0.10 |
| FLA | 4 B | AX_110621076 | 1D | C/G | 8.47-8.62 | 2.97E-04 | 0.08 |
| FLA | 4 B | AX_111021905 | 1D | T/C | 249.83-249.83 | 6.07E-04 | 0.07 |
| FLA | 3 B | AX_94853121 | 1D | T/C | 423.3-432.71 | 1.85E-04 | 0.08 |
| FLA | 3 4 B | AX_109407628 | 2A | A/G | 7.80-7.80 | 2.27E-05 | 0.11 |
| FLA | 4 B | AX_108756384 | 2A | G/C | 711.97-711.97 | 8.37E-04 | 0.07 |
| FLA | 3 B | AX_111027654 | 2B | G/C | 50.29-50.29 | 9.79E-04 | 0.07 |
| FLA | 4 B | AX_111034643 | 2B | G/A | 115.20-115.20 | 8.29E-04 | 0.07 |
| FLA | 4 B | AX_110586277 | 2B | C/T | 408.34-410.95 | 5.34E-04 | 0.07 |
| FLA | 4 B | AX_108901219 | 2B | G/A | 639.22-639.97 | 5.59E-04 | 0.07 |
| FLA | 4 B | AX_109968096 | 2B | G/A | 742.46-748.70 | 8.86E-04 | 0.07 |
| FLA | 2 B | GENE_2324_85 | 2D | A/G | 628.60-634.47 | 1.84E-04 | 0.08 |
| FLA | 4 B | AX_110558769 | 3A | A/G | 580.42-580.42 | 3.18E-04 | 0.08 |
| FLA | 4 B | AX_109838665 | 3A | T/G | 644.80-645.76 | 9.28E-05 | 0.09 |
| FLA | 2 3 | AX_110472002 | 3A | C/T | 722.63-722.63 | 7.92E-06 | 0.13 |
| FLA | 3 B | AX_109958385 | 3B | C/A | 42.47-43.28 | 2.50E-04 | 0.08 |
| FLA | 4 B | AX_111606754 | 3B | A/G | 670.53-670.53 | 8.09E-04 | 0.07 |
| FLA | 4 B | AX_94922328 | 3D | T/C | 508.39-508.39 | 1.78E-04 | 0.09 |
| FLA | 4 B | AX_110370862 | 3D | C/T | 604.69-604.69 | 2.67E-04 | 0.08 |
| FLA | 4 B | AX_110077614 | 4A | T/C | 24.87-24.90 | 4.68E-04 | 0.07 |
| FLA | 4 B | D_contig26957_307 | 4B | A/C | 409.74-409.74 | 1.36E-04 | 0.09 |
| FLA | 4 B | AX_109445215 | 4D | G/A | 28.58-28.58 | 7.63E-04 | 0.07 |
| FLA | 4 B | AX_89398182 | 4D | G/A | 419.14-421.64 | 2.69E-04 | 0.08 |
| FLA | 4 B | AX_109370388 | 5A | A/G | 440.65-440.68 | 3.85E-04 | 0.08 |
| FLA | 4 B | Tdurum_contig71099_245 | 5B | G/A | 21.18-21.18 | 9.66E-04 | 0.07 |
| FLA | 4 B | AX_110529518 | 5B | T/C | 595.12-595.12 | 5.84E-04 | 0.07 |
| FLA | 4 B | AX_111137392 | 5D | G/C | 266.33-266.33 | 5.97E-04 | 0.07 |
| FLA | 2 3 | AX_94388289 | 5D | C/G | 319.22-319.22 | 1.40E-05 | 0.12 |
| FLA | 2 4 B | AX_111684798 | 6A | A/T | 545.65-545.65 | 4.73E-04 | 0.07 |
| FLA | 4 B | AX_109444200 | 6B | C/T | 0.17-6.25 | 2.61E-04 | 0.08 |
| FLA | 4 B | Excalibur_c20083_433 | 6B | C/A | 531.52-537.46 | 1.77E-04 | 0.09 |
| FLA | 4 B | AX_95170412 | 6D | G/C | 360.61-360.61 | 9.18E-04 | 0.07 |
| FLA | 4 B | Tdurum_contig75811_1629 | 7A | A/G | 3.85-3.85 | 2.26E-04 | 0.08 |
| FLA | 2 3 B | AX_110521898 | 7A | C/T | 115.32-116.62 | 1.10E-04 | 0.09 |
| FLA | 4 B | Kukri_c717_98 | 7A | G/A | 227.09-227.09 | 1.35E-04 | 0.09 |
| FLA | 4 B | AX_94603127 | 7A | A/G | 672.03-672.03 | 1.77E-05 | 0.11 |
| FLA | 4 B | AX_111021586 | 7B | T/C | 647.76-653.03 | 1.51E-05 | 0.12 |
| FLA | 2 4 B | AX_109947486 | 7B | A/G | 701.42-702.32 | 8.81E-05 | 0.09 |
| FLA | 4 B | AX_94788487 | 7D | T/G | 278.34-278.34 | 3.94E-04 | 0.08 |
| FLA | 4 B | AX_94749119 | 7D | A/C | 580.55-580.57 | 3.51E-05 | 0.11 |
| FLB | 4 B | BS00065001_51 | 1A | C/A | 96.43-96.43 | 4.11E-04 | 0.08 |
| FLB | 2 B | AX_109933999 | 1A | T/C | 493.92-493.92 | 4.31E-04 | 0.08 |
| FLB | 4 B | AX_94820947 | 1A | A/G | 551.98-551.98 | 6.09E-05 | 0.10 |
| FLB | 4 B | AX_94396437 | 1B | G/T | 104.72-104.72 | 6.77E-04 | 0.07 |
| FLB | 2 4 B | Tdurum_contig16606_648 | 1B | A/G | 633.14-643.18 | 7.39E-06 | 0.13 |
| FLB | 4 B | AX_111528646 | 1D | A/G | 423.77-423.77 | 8.49E-05 | 0.10 |
| FLB | 4 B | AX_94823257 | 1D | C/G | 460.74-460.86 | 7.23E-05 | 0.10 |
| FLB | 2 4 B | AX_94530488 | 1D | A/G | 485.89-485.89 | 9.22E-04 | 0.07 |
| FLB | 4 B | AX_109407628 | 2A | A/G | 7.80-16.40 | 4.01E-05 | 0.11 |
| FLB | 4 B | BS00075524_51 | 2A | A/G | 42.39-42.39 | 9.81E-04 | 0.07 |
| FLB | 2 4 B | Ku_c5710_310 | 2A | A/G | 605.80-605.80 | 5.36E-06 | 0.13 |
| FLB | 4 B | AX_109279074 | 2A | A/G | 734.16-734.35 | 5.11E-04 | 0.08 |
| FLB | 4 B | AX_110987531 | 2A | G/A | 756.21-759.45 | 4.47E-04 | 0.08 |
| FLB | 2 4 B | Kukri_c26697_366 | 2A | T/C | 776.55-780.71 | 3.12E-05 | 0.11 |
| FLB | 4 B | AX_109393283 | 2B | A/G | 2.41-10.62 | 8.10E-04 | 0.07 |
| FLB | 4 B | AX_108942936 | 2B | T/C | 30.27-30.27 | 3.13E-04 | 0.08 |
| FLB | 4 B | AX_94931753 | 2B | C/T | 283.29-283.29 | 9.79E-04 | 0.07 |
| FLB | 4 B | RFL_Contig5122_974 | 2B | A/G | 776.21-776.21 | 5.88E-04 | 0.07 |
| FLB | 4 B | RAC875_c35200_230 | 2D | G/A | 633.2-634.85 | 1.24E-04 | 0.09 |
| FLB | 2 4 B | Excalibur_c5097_1468 | 3A | C/T | 654.32-654.32 | 9.89E-04 | 0.07 |
| FLB | 2 4 B | AX_111005153 | 3A | G/A | 721.62-732.04 | 2.12E-05 | 0.12 |
| FLB | 1 2 4 B | AX_111511076 | 3B | G/A | 26.76-26.76 | 1.24E-04 | 0.09 |
| FLB | 4 B | Excalibur_c35645_587 | 3B | A/C | 43.28-43.28 | 2.21E-04 | 0.09 |
| FLB | 4 B | AX_109888602 | 3B | C/T | 59.92-68.30 | 6.21E-05 | 0.10 |
| FLB | 4 B | RFL_Contig738_557 | 3B | G/A | 140.43-141.13 | 2.59E-04 | 0.08 |
| FLB | 4 B | AX_110741718 | 3B | A/G | 625.19-625.57 | 8.72E-04 | 0.07 |
| FLB | 4 B | BS00010881_51 | 3D | G/A | 91.22-91.22 | 3.21E-04 | 0.08 |
| FLB | 4 B | BS00021850_51 | 3D | A/G | 596.09-596.71 | 6.03E-04 | 0.07 |
| FLB | 2 B | AX_110402183 | 4A | G/C | 612.07-612.07 | 4.86E-04 | 0.08 |
| FLB | 4 B | AX_109443083 | 4A | G/A | 628.00-628.00 | 5.82E-04 | 0.07 |
| FLB | 4 B | AX_94631782 | 4A | G/A | 738.75-739.07 | 2.03E-05 | 0.12 |
| FLB | 4 B | wsnp_Ex_c16389_24884851 | 4B | G/A | 14.12-14.12 | 4.15E-05 | 0.11 |
| FLB | 2 4 B | AX_89323611 | 4B | A/G | 32.9-35.63 | 1.23E-05 | 0.12 |
| FLB | 4 B | RAC875_c27160_307 | 4D | G/A | 7.83-10.26 | 6.54E-05 | 0.10 |
| FLB | 2 4 B | AX_111837027 | 4D | T/C | 31.4-43.46 | 2.99E-05 | 0.11 |
| FLB | 4 B | AX_110554000 | 4D | G/A | 499.10-499.10 | 4.57E-04 | 0.08 |
| FLB | 4 B | AX_109438774 | 5A | T/C | 384.43-384.43 | 8.36E-05 | 0.10 |
| FLB | 2 B | AX_111110219 | 5A | A/G | 503.05-503.05 | 4.50E-04 | 0.08 |
| FLB | 2 4 B | AX_94456702 | 5A | C/T | 706.93-706.96 | 1.97E-04 | 0.09 |
| FLB | 4 B | AX_109825762 | 5B | C/A | 258.50-258.50 | 4.13E-04 | 0.08 |
| FLB | 4 B | AX_111731367 | 5B | G/A | 445.19-445.19 | 4.69E-04 | 0.08 |
| FLB | 2 4 B | AX_94388289 | 5D | C/G | 319.22-319.22 | 2.27E-05 | 0.11 |
| FLB | 2 4 B | AX_111684798 | 6A | A/T | 544.18-545.65 | 6.62E-05 | 0.10 |
| FLB | 4 B | GENE_3980_221 | 6A | G/A | 570.38-570.38 | 6.29E-04 | 0.07 |
| FLB | 4 B | AX_94626844 | 6B | C/T | 233.03-233.03 | 5.92E-04 | 0.07 |
| FLB | 4 B | AX_108851753 | 6B | G/A | 472.41-472.61 | 1.69E-04 | 0.09 |
| FLB | 4 B | Excalibur_c20083_433 | 6B | C/A | 537.46-537.46 | 1.57E-04 | 0.09 |
| FLB | 4 B | AX_111182870 | 6B | G/C | 549.42-549.42 | 7.41E-04 | 0.07 |
| FLB | 4 B | BobWhite_c33227_82 | 6B | G/A | 630.90-643.79 | 2.73E-04 | 0.08 |
| FLB | 4 B | Tdurum_contig75811_1629 | 7A | A/G | 1.70-14.19 | 8.91E-07 | 0.16 |
| FLB | 4 B | AX_108858189 | 7A | G/C | 45.42-45.42 | 5.44E-04 | 0.08 |
| FLB | 2 4 B | AX_110986916 | 7A | G/A | 115.32-116.60 | 3.46E-05 | 0.11 |
| FLB | 4 B | AX_109465513 | 7B | G/T | 676.48-702.32 | 3.65E-04 | 0.08 |
| FLB | 4 B | RAC875_c26706_504 | 7D | C/A | 4.52-15.78 | 1.51E-05 | 0.12 |
| FSLA | 4 B | AX_111578749 | 1A | T/A | 450.67-456.28 | 2.71E-04 | 0.08 |
| FSLA | 4 B | AX_109883429 | 1A | A/C | 512.67-512.67 | 6.09E-04 | 0.07 |
| FSLA | 4 B | AX_94820947 | 1A | A/G | 551.98-551.98 | 4.29E-04 | 0.08 |
| FSLA | 4 B | AX_109292633 | 1B | T/C | 272.97-272.97 | 8.49E-04 | 0.07 |
| FSLA | 4 B | AX_109898292 | 1B | G/T | 299.80-302.78 | 3.50E-04 | 0.08 |
| FSLA | 4 B | AX_110027217 | 1B | A/C | 310.19-314.57 | 5.22E-05 | 0.10 |
| FSLA | 4 B | AX_108977581 | 1B | G/C | 340.43-345.39 | 1.18E-04 | 0.09 |
| FSLA | 4 B | AX_109412901 | 1B | A/G | 357.92-364.02 | 1.75E-04 | 0.09 |
| FSLA | 2 B | AX_94510790 | 1B | T/G | 427.53-427.53 | 9.78E-04 | 0.07 |
| FSLA | 4 B | AX_109395396 | 1B | T/C | 494.23-494.23 | 7.31E-04 | 0.07 |
| FSLA | 4 B | AX_95255439 | 1D | C/T | 460.79-460.79 | 2.49E-04 | 0.08 |
| FSLA | 4 B | AX_94563363 | 3A | A/C | 730.28-730.81 | 3.05E-04 | 0.08 |
| FSLA | 1 4 B | AX_111511076 | 3B | G/A | 24.56-27.29 | 4.48E-05 | 0.10 |
| FSLA | 4 B | AX_108896845 | 3B | C/T | 40.53-40.53 | 2.24E-04 | 0.08 |
| FSLA | 4 B | BS00077528_51 | 3B | A/G | 59.92-68.30 | 1.64E-04 | 0.09 |
| FSLA | 4 B | wsnp_Ex_c1097_2105311 | 3B | C/T | 79.95-82.45 | 9.57E-05 | 0.09 |
| FSLA | 4 B | AX_109554130 | 3B | T/C | 125.93-128.36 | 5.32E-04 | 0.07 |
| FSLA | 4 B | wsnp_Ex_c4888_8714379 | 3B | C/T | 133.82-140.69 | 3.97E-05 | 0.11 |
| FSLA | 4 B | AX_109998261 | 3B | C/T | 161.30-161.30 | 5.95E-04 | 0.07 |
| FSLA | 1 2 | AX_89744241 | 3B | C/T | 493.65-493.65 | 2.88E-04 | 0.08 |
| FSLA | 4 B | AX_109951787 | 3B | A/G | 627.71-627.74 | 7.95E-04 | 0.07 |
| FSLA | 4 B | AX_110594258 | 3B | G/A | 788.40-788.40 | 8.00E-04 | 0.07 |
| FSLA | 4 B | BS00010881_51 | 3D | G/A | 84.91-91.22 | 2.44E-04 | 0.08 |
| FSLA | 4 B | Kukri_c20012_1362 | 4A | G/A | 628.33-628.33 | 7.04E-06 | 0.13 |
| FSLA | 4 B | AX_111067369 | 4A | G/T | 641.82-641.82 | 4.21E-04 | 0.08 |
| FSLA | 4 B | AX_109433453 | 4B | G/A | 4.30-4.30 | 8.76E-04 | 0.07 |
| FSLA | 4 B | AX_94787034 | 5A | C/T | 466.99-466.99 | 1.17E-04 | 0.09 |
| FSLA | 4 B | AX_110468146 | 5A | G/A | 520.10-528.78 | 2.03E-04 | 0.09 |
| FSLA | 4 B | AX_109940500 | 5B | T/C | 635.92-635.92 | 4.14E-04 | 0.08 |
| FSLA | 4 B | BS00029540_51 | 5B | A/C | 660.37-660.37 | 4.71E-04 | 0.08 |
| FSLA | 4 B | AX_95118822 | 5D | A/G | 320.89-320.89 | 8.01E-04 | 0.07 |
| FSLA | 4 B | AX_111609556 | 6B | G/A | 445.01-450.90 | 1.14E-04 | 0.09 |
| FSLA | 4 B | AX_109877327 | 6B | A/C | 461.86-462.02 | 1.44E-04 | 0.09 |
| FSLA | 4 B | AX_95684596 | 6B | C/T | 640.91-640.91 | 9.86E-04 | 0.07 |
| FSLA | 1 3 | AX_110389349 | 6B | C/A | 705.57-705.57 | 2.78E-04 | 0.08 |
| FSLA | 4 B | AX_110373305 | 7A | A/G | 33.51-33.66 | 1.93E-05 | 0.11 |
| FSLA | 4 B | BobWhite_c20735_255 | 7B | G/A | 2.79-2.79 | 4.99E-04 | 0.07 |
| FSLA | 4 B | AX_111022287 | 7D | G/A | 576.75-588.62 | 4.17E-04 | 0.08 |
| FLANG | 4 B | AX_89741392 | 1A | G/T | 593.31-593.31 | 7.35E-04 | 0.05 |
| FLANG | 3 4 B | wsnp_Ex_c1440_2764269 | 1B | G/A | 551.03-551.18 | 2.05E-04 | 0.06 |
| FLANG | 4 B | AX_111761207 | 1B | G/A | 581.21-586.30 | 5.74E-06 | 0.10 |
| FLANG | 4 B | AX_108750578 | 1D | C/T | 100.09-100.09 | 8.97E-04 | 0.05 |
| FLANG | 3 B | AX_109915932 | 2A | G/A | 713.22-713.64 | 1.78E-04 | 0.07 |
| FLANG | 4 B | Kukri_c50391_219 | 2A | G/A | 750.57-754.47 | 2.81E-04 | 0.06 |
| FLANG | 1 B | tplb0060e06_691 | 2A | G/A | 767.78-767.78 | 3.36E-04 | 0.06 |
| FLANG | 3 B | AX_111455070 | 2B | T/C | 746.69-748.43 | 2.41E-04 | 0.06 |
| FLANG | 3 B | AX_110086276 | 2B | C/A | 753.95-762.06 | 1.49E-04 | 0.07 |
| FLANG | 4 B | BobWhite_c44561_338 | 2D | A/G | 621.06-621.06 | 1.87E-04 | 0.06 |
| FLANG | 1 2 | AX_109880438 | 3A | T/C | 526.52-526.52 | 3.76E-04 | 0.06 |
| FLANG | 3 B | AX_111232584 | 3B | T/C | 281.68-287.77 | 4.75E-04 | 0.06 |
| FLANG | 2 B | AX_108837674 | 3B | T/C | 298.68-298.68 | 6.88E-04 | 0.05 |
| FLANG | 2 B | AX_109283867 | 3B | A/C | 319.19-319.19 | 2.05E-04 | 0.06 |
| FLANG | 1 2 3 B | AX_109850122 | 3B | A/G | 371.25-378.35 | 2.23E-04 | 0.06 |
| FLANG | 3 4 B | AX_109036884 | 3B | C/T | 381.96-392.85 | 1.49E-05 | 0.09 |
| FLANG | 2 3 B | AX_110551935 | 3B | G/C | 403.96-413.84 | 4.50E-05 | 0.08 |
| FLANG | 1 B | AX_111112688 | 3B | G/A | 763.81-764.75 | 1.31E-04 | 0.07 |
| FLANG | 1 B | AX_109008933 | 4B | A/G | 9.95-9.95 | 8.90E-04 | 0.05 |
| FLANG | 1 B | AX_108806641 | 4B | C/T | 649.60-649.65 | 7.88E-04 | 0.05 |
| FLANG | 2 B | AX_109377374 | 5A | A/G | 3.52-3.52 | 5.23E-04 | 0.06 |
| FLANG | 1 B | AX_109937699 | 5A | G/C | 546.53-546.53 | 8.41E-04 | 0.05 |
| FLANG | 3 B | RAC875_c52775_537 | 5A | A/G | 646.21-646.21 | 1.48E-04 | 0.07 |
| FLANG | 4 B | GENE-2826_325 | 5A | A/C | 688.32-693.66 | 8.44E-04 | 0.05 |
| FLANG | 1 B | AX_111067225 | 5A | G/A | 704.61-704.61 | 6.32E-04 | 0.05 |
| FLANG | 3 B | AX_110651444 | 5B | T/G | 577.51-583.71 | 3.28E-04 | 0.06 |
| FLANG | 3 B | BobWhite_c967_516 | 5D | G/A | 519.65-519.65 | 6.56E-05 | 0.07 |
| FLANG | 2 3 B | BobWhite_c11042_284 | 6B | T/G | 117.85-127.32 | 5.94E-05 | 0.08 |
| FLANG | 3 B | BS00011624_51 | 6B | G/A | 164.39-164.39 | 8.72E-04 | 0.05 |
| FLANG | 3 B | IAAV8065 | 6B | A/G | 411.10-411.10 | 7.74E-05 | 0.07 |
| FLANG | 4 B | AX_110130742 | 6B | G/A | 708.19-708.19 | 2.57E-04 | 0.06 |
| FLANG | 3 4 B | AX_109834623 | 6D | G/A | 281.36-284.60 | 4.70E-04 | 0.06 |
| FLANG | 1 3 4 B | AX_111886404 | 6D | C/T | 323.83-329.85 | 3.45E-05 | 0.08 |
| FLANG | 4 B | AX_109651626 | 6D | G/A | 338.24-345.78 | 1.31E-04 | 0.07 |
| FLANG | 1 3 4 B | AX_109936507 | 6D | G/A | 352.84-357.01 | 1.46E-06 | 0.11 |
| FLANG | 1 3 B | AX_111263460 | 6D | A/G | 388.23-390.60 | 4.16E-05 | 0.08 |
| FLANG | 1 3 B | AX_111157528 | 7A | T/C | 78.47-78.47 | 2.60E-04 | 0.06 |
| FLANG | 1 B | AX_110414779 | 7B | A/C | 128.34-128.34 | 2.48E-04 | 0.06 |
| FLANG | 1 B | GENE-4790_279 | 7B | T/G | 162.12-164.10 | 1.80E-04 | 0.07 |
| FLANG | 2 B | AX_111015607 | 7B | G/A | 334.81-336.65 | 5.47E-04 | 0.06 |
| FLANG | 1 B | AX_110277705 | 7D | G/C | 195.65-195.65 | 7.18E-04 | 0.05 |
| FLANG | 3 B | AX_110388025 | 7D | A/G | 366.72-366.72 | 6.21E-04 | 0.05 |
| SPAD | 1 B | AX_110946052 | 1A | C/T | 579.15-580.09 | 4.05E-04 | 0.07 |
| SPAD | 1 B | BS00067567_51 | 1B | A/G | 40.88-41.64 | 3.22E-04 | 0.07 |
| SPAD | 1 B | IAAV2125 | 1B | A/C | 494.16-494.16 | 9.84E-04 | 0.06 |
| SPAD | 1 B | GENE-0120_431 | 1B | A/G | 551.01-551.01 | 8.86E-04 | 0.06 |
| SPAD | 1 B | BS00076405_51 | 1B | T/G | 563.54-563.54 | 9.21E-04 | 0.06 |
| SPAD | 1 B | BS00078897_51 | 1D | A/G | 26.41-27.87 | 1.50E-04 | 0.08 |
| SPAD | 2 B | RAC875_c28108_400 | 2B | G/A | 681.54-681.54 | 5.96E-04 | 0.06 |
| SPAD | 2 B | Ex_c16948_754 | 2B | A/G | 699.83-699.83 | 5.25E-04 | 0.07 |
| SPAD | 1 2 B | AX_108854529 | 3B | A/G | 20.82-20.82 | 8.87E-04 | 0.06 |
| SPAD | 1 B | BS00090227_51 | 3B | A/G | 133.82-133.82 | 4.43E-04 | 0.07 |
| SPAD | 3 B | AX_111059640 | 3B | C/A | 257.78-257.78 | 2.68E-05 | 0.10 |
| SPAD | 3 B | AX_109460386 | 3B | G/A | 270.56-279.86 | 3.86E-05 | 0.09 |
| SPAD | 3 B | AX_108935135 | 3B | C/T | 291.94-297.91 | 7.17E-05 | 0.09 |
| SPAD | 3 B | AX_110057844 | 3B | C/G | 309.37-312.66 | 5.64E-05 | 0.09 |
| SPAD | 3 B | AX_110543934 | 3B | C/A | 331.55-337.42 | 5.52E-05 | 0.09 |
| SPAD | 3 B | AX_111019875 | 3B | C/A | 350.65-354.28 | 1.85E-04 | 0.08 |
| SPAD | 3 B | AX_111517855 | 3B | T/C | 365.13-367.49 | 3.44E-05 | 0.10 |
| SPAD | 3 B | AX_109291420 | 3B | A/G | 375.49-375.49 | 1.14E-04 | 0.08 |
| SPAD | 1 B | AX_108997469 | 3B | C/T | 405.23-405.23 | 5.35E-04 | 0.07 |
| SPAD | 1 B | BS00095515_51 | 3B | A/G | 772.40-772.40 | 1.42E-04 | 0.08 |
| SPAD | 3 B | AX_109314274 | 3D | T/G | 226.61-226.61 | 7.64E-04 | 0.06 |
| SPAD | 2 B | AX_95089051 | 4A | T/C | 594.69-594.69 | 2.05E-04 | 0.08 |
| SPAD | 2 3 B | AX_94704307 | 4B | G/A | 16.06-16.06 | 2.89E-04 | 0.07 |
| SPAD | 1 2 B | BobWhite_c11512_157 | 5A | G/A | 514.09-522.07 | 1.24E-04 | 0.08 |
| SPAD | 4 B | RAC875_c26353_719 | 5A | A/G | 591.09-591.09 | 7.93E-04 | 0.06 |
| SPAD | 1 2 B | BS00065313_51 | 5B | G/A | 489.85-489.85 | 1.90E-04 | 0.08 |
| SPAD | 1 B | AX_108853112 | 6B | C/T | 157.15-157.15 | 4.08E-04 | 0.07 |
| SPAD | 3 B | wsnp_Ra_c13881_21836489 | 6D | G/A | 409.34-409.34 | 4.73E-04 | 0.07 |
| SPAD | 1 B | AX_108853112 | 6B | C/T | 157.15-157.15 | 4.08E-04 | 0.07 |
| SPAD | 2 B | AX_109486280 | 6B | G/A | 694.25-694.25 | 7.80E-04 | 0.06 |
| SPAD | 3 B | wsnp_Ra_c13881_21836489 | 6D | G/A | 409.34-409.34 | 4.73E-04 | 0.07 |
| SPAD | 3 4 B | BS00106233_51 | 6D | A/G | 464.68-466.95 | 4.11E-05 | 0.09 |
| SPAD | 3 B | Excalibur_rep_c69170_602 | 7A | G/A | 6.50-8.36 | 7.54E-06 | 0.11 |
| SPAD | 3 B | AX_111728563 | 7A | T/C | 703.56-703.56 | 6.81E-04 | 0.06 |
| Pn | 3 B | AX_109297511 | 1A | T/C | 517.37-517.51 | 2.51E-04 | 0.08 |
| Pn | 1 2 B | AX_110927197 | 2A | G/C | 31.83-31.92 | 4.56E-04 | 0.07 |
| Pn | 1 B | AX_111100751 | 3A | A/G | 127.66-127.66 | 6.39E-04 | 0.07 |
| Pn | 1 B | AX_111084276 | 3A | G/C | 137.59-137.59 | 2.40E-04 | 0.08 |
| Pn | 1 B | AX_110933812 | 3A | A/G | 503.02-504.06 | 4.15E-04 | 0.07 |
| Pn | 4 B | AX_108929498 | 3A | G/A | 621.88-624.06 | 1.86E-04 | 0.08 |
| Pn | 4 B | AX_95111995 | 3B | G/T | 791.29-791.29 | 1.72E-04 | 0.08 |
| Pn | 2 B | AX_94436454 | 3D | T/C | 571.23-571.55 | 7.33E-04 | 0.07 |
| Pn | 1 B | AX_110514195 | 4A | C/T | 37.05-37.30 | 3.67E-04 | 0.07 |
| Pn | 1 B | AX_109377374 | 5A | A/G | 3.52-3.52 | 6.70E-04 | 0.07 |
| Pn | 4 B | AX_108940459 | 5A | T/C | 117.39-117.39 | 2.79E-04 | 0.08 |
| Pn | 1 2 B | AX_108987083 | 5B | G/T | 575.97-576.92 | 8.40E-04 | 0.06 |
| Pn | 2 B | AX_111152478 | 6B | A/T | 614.43-614.43 | 8.27E-04 | 0.07 |
| Pn | 3 B | BobWhite_c11312_346 | 6D | A/G | 46.85-46.85 | 1.02E-04 | 0.09 |
| Pn | 2 B | AX_109568098 | 7A | C/G | 287.70-287.70 | 6.41E-06 | 0.12 |
| Pn | 2 B | AX_111235557 | 7A | G/T | 451.90-451.90 | 1.32E-04 | 0.09 |
| Pn | 2 B | AX_109329047 | 7A | G/A | 478.98-484.46 | 2.60E-04 | 0.08 |
| Pn | 3 B | AX_110898812 | 7D | T/C | 495.10-495.10 | 6.36E-04 | 0.07 |
| Pn | 4 B | AX_111745444 | 7D | T/C | 634.35-634.35 | 2.96E-05 | 0.10 |
| Gs | 2 B | AX_108731433 | 1A | A/C | 450.04-451.56 | 1.98E-04 | 0.08 |
| Gs | 2 B | AX_110520028 | 1A | G/T | 461.52-461.52 | 7.18E-04 | 0.06 |
| Gs | 3 B | BS00067567_51 | 1B | A/G | 40.88-40.88 | 9.86E-04 | 0.06 |
| Gs | 2 B | Kukri_c57942_177 | 1D | A/G | 7.87-7.87 | 1.38E-04 | 0.08 |
| Gs | 2 B | AX_110928042 | 2B | T/C | 419.96-419.96 | 9.83E-04 | 0.06 |
| Gs | 2 B | AX_86162339 | 2B | T/C | 453.27-453.27 | 9.13E-04 | 0.06 |
| Gs | 3 B | AX_111265322 | 2D | A/T | 9.35-9.35 | 3.50E-04 | 0.07 |
| Gs | 1 B | AX_110010295 | 2D | C/G | 415.60-415.60 | 8.20E-04 | 0.06 |
| Gs | 3 B | AX_94769054 | 3A | A/G | 23.28-23.28 | 1.53E-04 | 0.08 |
| Gs | 1 B | AX_111009532 | 3A | C/G | 429.63-430.07 | 7.98E-05 | 0.09 |
| Gs | 2 B | AX_110461571 | 3B | G/A | 240.52-240.52 | 4.24E-04 | 0.07 |
| Gs | 1 B | AX_111710971 | 5B | G/A | 587.12-587.12 | 4.58E-04 | 0.07 |
| Gs | 2 B | AX_111481734 | 6B | G/C | 581.94-581.94 | 3.66E-04 | 0.07 |
| Ci | 2 B | AX_94466632 | 1A | C/A | 402.94-403.14 | 3.31E-04 | 0.08 |
| Ci | 2 B | AX_110953451 | 1A | G/C | 424.27-424.27 | 7.85E-04 | 0.07 |
| Ci | 3 B | AX_94769054 | 3A | A/G | 23.28-23.33 | 3.18E-04 | 0.08 |
| Ci | 4 B | AX_108884671 | 3A | C/T | 538.01-538.01 | 4.02E-04 | 0.08 |
| Ci | 3 B | AX_109395414 | 3A | G/A | 633.95-633.95 | 3.81E-04 | 0.08 |
| Ci | 4 B | AX_111539354 | 4B | G/A | 650.58-650.58 | 9.27E-04 | 0.07 |
| Ci | 3 B | AX_110048734 | 7A | A/G | 12.75-12.91 | 3.81E-04 | 0.08 |
| Ci | 3 B | AX_110452418 | 7B | C/A | 648.15-648.15 | 8.32E-04 | 0.07 |
| Tr | 1 3 | CAP7_c3299_316 | 1A | A/G | 11.67-11.67 | 3.30E-04 | 0.08 |
| Tr | 1 B | AX_111510429 | 2B | T/C | 2.63-2.63 | 4.35E-04 | 0.07 |
| Tr | 3 B | AX_108911571 | 2B | C/T | 794.50-794.50 | 4.34E-04 | 0.07 |
| Tr | 4 B | AX_111441702 | 2D | G/A | 10.72-10.84 | 6.06E-05 | 0.09 |
| Tr | 1 B | AX_110822097 | 2D | C/T | 647.82-647.82 | 1.90E-04 | 0.08 |
| Tr | 3 B | AX_94769054 | 3A | A/G | 15.62-23.3 | 1.36E-04 | 0.08 |
| Tr | 1 B | AX_95222692 | 3D | C/T | 258.27-258.27 | 5.62E-04 | 0.07 |
| Tr | 3 B | AX_94659839 | 5A | C/G | 117.86-118.67 | 2.74E-04 | 0.08 |
| Tr | 1 2 | AX_111705127 | 5B | G/A | 580.09-580.10 | 2.96E-04 | 0.08 |
| Tr | 3 B | AX_109309794 | 6D | C/T | 435.77-435.77 | 5.18E-04 | 0.07 |
| Tr | 3 B | AX_110452418 | 7B | C/A | 648.15-648.15 | 3.76E-04 | 0.07 |
| Tr | 3 B | AX_94659839 | 5A | C/G | 117.86-118.67 | 2.74E-04 | 0.08 |
| Tr | 1 2 | AX_111705127 | 5B | G/A | 580.09-580.10 | 2.96E-04 | 0.08 |
| Tr | 3 B | AX_109309794 | 6D | C/T | 435.77-435.77 | 5.18E-04 | 0.07 |
| Tr | 3 B | AX_110452418 | 7B | C/A | 648.15-648.15 | 3.76E-04 | 0.07 |
| WUE | 3 B | AX_111173119 | 1A | A/G | 369.52-385.90 | 9.66E-05 | 0.10 |
| WUE | 4 B | AX_110509400 | 1B | G/A | 680.87-680.87 | 6.67E-04 | 0.07 |
| WUE | 3 B | AX_108770196 | 1D | A/G | 303.32-303.32 | 7.37E-04 | 0.07 |
| WUE | 3 B | AX_108969307 | 2D | C/T | 470.83-471.91 | 7.73E-04 | 0.07 |
| WUE | 1 B | AX_109009376 | 3B | C/T | 571.75-571.75 | 4.56E-04 | 0.08 |
| WUE | 3 B | BS00034174_51 | 4A | A/G | 562.41-562.41 | 4.97E-04 | 0.08 |
| WUE | 4 B | AX_111572782 | 6B | G/C | 32.5-34.93 | 9.14E-04 | 0.07 |
| WUE | 2 B | AX_111017845 | 6B | C/A | 120.83-121.74 | 4.44E-04 | 0.08 |
| WUE | 3 B | AX_110494348 | 7B | C/A | 649.02-649.02 | 3.80E-04 | 0.08 |
| iWUE | 3 B | BS00067567_51 | 1B | A/G | 40.88-40.88 | 9.22E-04 | 0.06 |
| iWUE | 4 B | AX_86177029 | 2B | C/T | 773.35-773.35 | 2.82E-04 | 0.08 |
| iWUE | 4 B | AX_108884671 | 3A | C/T | 538.01-539.84 | 1.11E-04 | 0.09 |
| iWUE | 3 B | wsnp_Ex_rep_c66685_65003254 | 3A | A/G | 571.41-571.41 | 2.35E-04 | 0.08 |
| iWUE | 2 B | AX_109299591 | 5A | A/G | 11.55-11.71 | 2.71E-04 | 0.08 |
| iWUE | 4 B | AX_109858444 | 6A | A/G | 0.77-0.77 | 1.45E-04 | 0.08 |
| Fv'/Fm' | 1 B | AX_110683989 | 2A | C/A | 739.31-739.38 | 4.69E-04 | 0.08 |
| Fv'/Fm' | 2 B | AX_109926462 | 3A | C/T | 574.51-574.99 | 1.00E-04 | 0.10 |
| Fv'/Fm' | 2 B | AX_110558769 | 3A | A/G | 580.42-580.42 | 6.63E-04 | 0.08 |
| Fv'/Fm' | 2 B | Tdurum_contig57552_327 | 3A | A/G | 644.80-645.61 | 2.21E-04 | 0.09 |
| Fv'/Fm' | 2 B | AX_111046831 | 3A | T/C | 659.86-662.81 | 6.46E-04 | 0.08 |
| Fv'/Fm' | 2 B | AX_110960937 | 3B | C/A | 615.88-615.88 | 4.50E-04 | 0.08 |
| Fv'/Fm' | 2 B | AX_110361552 | 3B | C/A | 818.52-818.75 | 1.06E-04 | 0.10 |
| PH | 2 B | AX_110389477 | 1A | G/A | 79.14-79.14 | 6.49E-04 | 0.06 |
| PH | 3 4 B | AX_109279684 | 1A | G/A | 108.32-114.79 | 1.90E-04 | 0.07 |
| PH | 3 4 B | AX_110168764 | 1A | G/A | 132.42-135.71 | 3.44E-04 | 0.06 |
| PH | 3 4 B | AX_109901254 | 1A | G/C | 142.57-150.90 | 5.14E-04 | 0.06 |
| PH | 3 4 B | AX_110172892 | 1A | C/T | 159.86-162.53 | 2.92E-04 | 0.06 |
| PH | 3 4 B | AX_109457230 | 1A | G/A | 171.02-180.04 | 1.15E-04 | 0.07 |
| PH | 3 4 B | AX_109576385 | 1A | C/G | 190.81-194.24 | 4.42E-04 | 0.06 |
| PH | 3 4 B | AX_111794740 | 1A | G/A | 204.42-209.71 | 2.15E-04 | 0.07 |
| PH | 3 4 B | AX_109077125 | 1A | G/A | 215.46-217.61 | 3.44E-04 | 0.06 |
| PH | 3 4 B | AX_111075006 | 1A | C/G | 220.84-230.78 | 3.44E-04 | 0.06 |
| PH | 3 B | AX_111561676 | 1A | C/T | 246.69-246.69 | 9.76E-04 | 0.05 |
| PH | 3 B | AX_108951092 | 1A | G/A | 353.37-355.33 | 7.49E-04 | 0.05 |
| PH | 3 4 B | AX_110393681 | 1A | C/G | 368.20-368.20 | 3.19E-04 | 0.06 |
| PH | 2 3 4 B | AX_111172275 | 1A | A/G | 386.79-396.25 | 5.31E-06 | 0.10 |
| PH | 2 3 4 B | AX_110577878 | 1A | A/G | 432.95-442.81 | 1.29E-04 | 0.07 |
| PH | 2 4 B | AX_109935428 | 1A | A/G | 477.73-478.90 | 3.13E-04 | 0.06 |
| PH | 1 2 4 B | AX_111652875 | 1A | C/T | 516.73-517.52 | 5.30E-05 | 0.08 |
| PH | 4 B | Kukri_c43972_367 | 1A | A/G | 534.05-534.05 | 8.95E-04 | 0.05 |
| PH | 1 2 B | BS00032825_51 | 1A | C/A | 557.10-557.10 | 9.71E-04 | 0.05 |
| PH | 1 2 | AX_111495928 | 1A | A/G | 570.98-571.9 | 1.54E-04 | 0.07 |
| PH | 1 2 3 B | BS00067567_51 | 1B | A/G | 32.77-40.88 | 1.56E-04 | 0.07 |
| PH | 1 3 B | AX_111532632 | 1B | A/G | 514.35-515.51 | 4.17E-04 | 0.06 |
| PH | 1 3 4 B | AX_94564150 | 1B | G/A | 532.67-540.84 | 3.67E-05 | 0.08 |
| PH | 1 2 B | AX_94505424 | 1B | T/C | 613.32-615.52 | 1.37E-04 | 0.07 |
| PH | 1 2 4 B | AX_109458178 | 1B | A/G | 668.10-674.81 | 5.89E-05 | 0.08 |
| PH | 2 3 4 B | AX_110445616 | 1B | G/A | 681.86-681.86 | 2.60E-04 | 0.06 |
| PH | 2 4 B | AX_94774611 | 1D | T/C | 26.40-27.87 | 3.40E-04 | 0.06 |
| PH | 1 2 3 B | AX_86177385 | 1D | T/G | 401.68-401.68 | 9.84E-05 | 0.07 |
| PH | 1 2 3 B | Kukri_c82086_387 | 1D | T/G | 455.80-455.80 | 9.62E-04 | 0.05 |
| PH | 1 2 3 B | AX_94638736 | 2A | A/C | 30.87-32.87 | 1.74E-04 | 0.07 |
| PH | 1 2 B | Tdurum_contig11960_209 | 2A | A/G | 156.62-156.62 | 5.99E-04 | 0.06 |
| PH | 1 B | AX_108840536 | 2A | G/T | 422.13-426.64 | 7.98E-04 | 0.05 |
| PH | 1 B | AX_111599471 | 2A | T/C | 455.34-455.34 | 8.11E-04 | 0.05 |
| PH | 1 2 B | AX_109324498 | 2A | C/G | 476.06-488.93 | 7.27E-04 | 0.05 |
| PH | 1 2 B | AX_108732902 | 2A | G/A | 492.68-496.47 | 2.34E-04 | 0.06 |
| PH | 1 2 B | AX_110073211 | 2A | G/A | 500.86-505.21 | 5.71E-04 | 0.06 |
| PH | 1 2 B | AX_109952849 | 2A | C/A | 511.12-514.40 | 5.18E-04 | 0.06 |
| PH | 1 2 B | AX_109851286 | 2A | T/G | 538.47-542.64 | 4.05E-04 | 0.06 |
| PH | 2 B | AX_110476771 | 2A | A/G | 713.02-713.02 | 6.39E-04 | 0.06 |
| PH | 4 B | Kukri_rep_c68159_218 | 2B | G/A | 11.76-11.76 | 3.10E-04 | 0.06 |
| PH | 1 2 B | Excalibur_c15696_1280 | 2B | G/A | 202.74-206.12 | 2.35E-04 | 0.06 |
| PH | 1 2 4 B | Ex_c4328_1408 | 2B | A/G | 212.30-216.47 | 4.72E-05 | 0.08 |
| PH | 2 B | AX_110996707 | 2B | G/C | 703.66-704.30 | 2.26E-04 | 0.06 |
| PH | 1 2 B | AX_109967403 | 2B | G/A | 725.14-725.14 | 4.04E-04 | 0.06 |
| PH | 1 4 B | AX_110934424 | 2B | T/C | 745.70-745.70 | 5.61E-04 | 0.06 |
| PH | 3 B | AX_109064967 | 2B | G/C | 767.32-769.93 | 5.94E-04 | 0.06 |
| PH | 1 2 4 B | GENE_1313_319 | 2D | G/A | 151.73-159.69 | 1.13E-04 | 0.07 |
| PH | 1 3 B | AX_110700052 | 2D | A/G | 626.65-626.65 | 2.42E-04 | 0.06 |
| PH | 1 2 3 B | AX_108859415 | 2D | A/G | 639.49-650.78 | 3.65E-04 | 0.06 |
| PH | 1 2 4 B | AX_108900466 | 3A | T/C | 717.65-721.34 | 6.42E-05 | 0.08 |
| PH | 1 2 3 B | AX_111015381 | 3A | A/T | 741.84-741.84 | 7.27E-04 | 0.05 |
| PH | 4 B | AX_108896845 | 3B | C/T | 40.53-40.53 | 8.83E-04 | 0.05 |
| PH | 1 2 B | BS00025114_51 | 3B | G/A | 79.95-82.45 | 7.83E-04 | 0.05 |
| PH | 1 2 B | AX_95176265 | 3B | T/C | 146.89-148.30 | 2.90E-04 | 0.06 |
| PH | 1 B | AX_109077546 | 3B | C/G | 156.62-158.69 | 8.29E-05 | 0.07 |
| PH | 1 2 B | AX_108845108 | 3B | C/A | 237.11-246.12 | 3.44E-04 | 0.06 |
| PH | 1 2 3 4 B | AX_111508679 | 3B | C/T | 280.11-288.09 | 1.10E-05 | 0.09 |
| PH | 1 2 3 4 B | AX_111072178 | 3B | G/A | 306.89-308.42 | 3.09E-05 | 0.08 |
| PH | 1 4 B | AX_110020743 | 3B | G/C | 314.2-318.47 | 1.96E-04 | 0.07 |
| PH | 1 2 3 B | AX_111147061 | 3B | C/T | 324.85-328.80 | 1.70E-04 | 0.07 |
| PH | 1 3 4 B | AX_111516763 | 3B | C/T | 358.13-362.52 | 1.17E-05 | 0.09 |
| PH | 1 3 B | AX_111574337 | 3B | A/G | 373.72-374.30 | 6.38E-05 | 0.08 |
| PH | 2 3 B | AX_108997469 | 3B | C/T | 405.23-405.23 | 8.07E-04 | 0.05 |
| PH | 1 2 B | AX_110372726 | 3B | C/T | 818.33-818.77 | 1.05E-05 | 0.09 |
| PH | 1 2 | AX_111481627 | 3D | T/C | 606.61-606.61 | 7.59E-04 | 0.05 |
| PH | 2 B | AX_108746526 | 4A | A/T | 24.77-29.06 | 4.62E-04 | 0.06 |
| PH | 1 2 B | AX_110030140 | 4A | A/G | 59.81-60.36 | 4.23E-04 | 0.06 |
| PH | 1 2 3 4 B | AX_109821180 | 4A | A/G | 381.25-381.25 | 3.00E-05 | 0.08 |
| PH | 2 B | Kukri_c35049_357 | 4A | A/G | 625.40-625.40 | 7.43E-04 | 0.05 |
| PH | 1 2 | AX_111114438 | 4A | G/C | 661.68-661.68 | 8.40E-04 | 0.05 |
| PH | 1 2 3 B | AX_109460468 | 4A | G/A | 735.30-735.30 | 2.72E-05 | 0.08 |
| PH | 1 2 B | IAAV8639 | 4B | G/A | 670.44-670.44 | 6.24E-04 | 0.06 |
| PH | 2 B | AX_89703298 | 4D | T/G | 16.75-19.29 | 4.25E-04 | 0.06 |
| PH | 2 B | AX_111663936 | 4D | C/T | 74.41-77.02 | 3.67E-04 | 0.06 |
| PH | 1 3 | AX_89743887 | 4D | A/G | 85.12-97.97 | 4.05E-04 | 0.07 |
| PH | 1 3 B | AX_109265317 | 4D | T/C | 373.59-373.59 | 1.52E-04 | 0.08 |
| PH | 1 2 3 B | AX_109311262 | 5A | G/A | 495.92-495.92 | 1.76E-04 | 0.07 |
| PH | 1 2 B | AX_111455418 | 5A | A/G | 520.76-521.82 | 4.94E-04 | 0.06 |
| PH | 2 B | AX_111477299 | 5A | G/A | 537.13-537.13 | 7.19E-04 | 0.05 |
| PH | 1 4 B | Tdurum_contig4576_1153 | 5A | C/A | 563.84-563.84 | 9.35E-05 | 0.07 |
| PH | 2 4 B | AX_111523027 | 5A | C/A | 631.67-631.67 | 3.09E-04 | 0.06 |
| PH | 1 2 4 B | BobWhite_c47401_491 | 5A | G/A | 703.20-708.77 | 4.64E-06 | 0.10 |
| PH | 1 2 B | AX_94603508 | 5B | G/A | 19.70-20.70 | 2.69E-04 | 0.06 |
| PH | 1 2 3 B | BS00063299_51 | 5B | C/A | 482.42-487.22 | 3.63E-05 | 0.08 |
| PH | 1 2 4 B | BobWhite_rep_c62475_70 | 5B | G/A | 531.15-533.49 | 7.18E-05 | 0.08 |
| PH | 3 4 B | AX_111475535 | 5B | G/A | 554.76-558.89 | 5.42E-05 | 0.08 |
| PH | 2 3 B | AX_94715126 | 5B | G/C | 577.23-578.59 | 2.38E-04 | 0.06 |
| PH | 2 3 | AX_111213250 | 5B | T/C | 595.64-595.64 | 2.81E-04 | 0.07 |
| PH | 2 4 B | AX_110946146 | 5B | C/T | 701.28-703.49 | 6.05E-05 | 0.08 |
| PH | 1 2 4 B | D_contig33819_563 | 5D | G/A | 249.79-249.79 | 8.89E-05 | 0.07 |
| PH | 1 4 B | Kukri_c9522_622 | 5D | G/A | 438.11-438.11 | 2.81E-04 | 0.06 |
| PH | 1 2 4 B | D_GBF1XID01CVZMX_132 | 5D | C/A | 483.52-483.52 | 2.14E-05 | 0.09 |
| PH | 2 4 B | AX_95222094 | 5D | G/C | 544.70-549.89 | 1.25E-04 | 0.07 |
| PH | 1 B | AX_111173364 | 5D | C/T | 559.92-560.01 | 7.24E-04 | 0.05 |
| PH | 1 4 B | BS00068513_51 | 6A | C/A | 184.82-184.82 | 1.20E-04 | 0.07 |
| PH | 4 B | AX_111644089 | 6A | G/A | 510.93-510.93 | 8.87E-04 | 0.05 |
| PH | 1 3 B | wsnp_Ex_rep_c67436_66026057 | 6A | A/G | 594.75-596.71 | 7.99E-06 | 0.10 |
| PH | 1 3 B | BS00011578_51 | 6A | A/G | 611.49-611.85 | 5.27E-04 | 0.06 |
| PH | 1 2 3 B | AX_108864685 | 6B | C/T | 0.49-8.89 | 1.04E-06 | 0.12 |
| PH | 1 2 3 B | AX_94915759 | 6D | T/C | 0.81-0.81 | 3.62E-05 | 0.08 |
| PH | 1 2 B | AX_109852543 | 6D | G/A | 46.5-56.55 | 4.90E-05 | 0.08 |
| PH | 1 2 3 B | AX_109928196 | 6D | C/G | 327.54-338.24 | 1.02E-04 | 0.07 |
| PH | 3 4 | AX_109331000 | 6D | A/G | 389.61-396.55 | 3.63E-04 | 0.07 |
| PH | 1 4 B | D_GCE8AKX01DVTYJ_306 | 6D | G/A | 434.61-434.61 | 1.85E-04 | 0.07 |
| PH | 1 4 B | wsnp_Ex_c14654_22713386 | 7A | A/G | 11.10-11.10 | 8.17E-05 | 0.07 |
| PH | 4 B | AX_109384874 | 7A | C/T | 557.65-557.65 | 3.96E-04 | 0.06 |
| PH | 1 2 3 | AX_110543412 | 7A | T/C | 662.97-663.45 | 2.15E-04 | 0.07 |
| PH | 3 B | AX_110914860 | 7B | C/A | 167.68-167.69 | 9.78E-04 | 0.05 |
| PH | 4 B | AX_109582117 | 7B | G/A | 213.57-213.57 | 7.67E-04 | 0.05 |
| PH | 4 B | AX_111187871 | 7B | G/A | 234.91-238.53 | 2.83E-04 | 0.06 |
| PH | 1 2 | AX_110163437 | 7B | T/C | 625.37-625.37 | 8.16E-04 | 0.05 |
| PH | 1 2 3 | AX_109406465 | 7B | C/T | 630.58-632.34 | 6.74E-05 | 0.08 |
| PH | 1 2 B | AX_110586060 | 7B | A/C | 701.91-702.56 | 1.52E-04 | 0.07 |
| PH | 1 4 B | AX_109412361 | 7B | G/T | 725.20-725.52 | 9.26E-05 | 0.07 |
| PH | 1 2 B | AX_94814963 | 7D | T/C | 171-171 | 8.35E-04 | 0.05 |
| LAI | 3 4 B | AX_110477131 | 1A | G/A | 463.04-465.5 | 6.77E-05 | 0.10 |
| LAI | 4 B | AX_111458014 | 1A | C/T | 473.01-474.05 | 3.14E-04 | 0.08 |
| LAI | 4 B | AX_110482470 | 1B | A/G | 27.28-27.28 | 4.72E-04 | 0.07 |
| LAI | 4 B | AX_108943492 | 1B | A/T | 363.66-363.66 | 7.43E-04 | 0.07 |
| LAI | 4 B | AX_94659265 | 1B | C/G | 498.85-498.85 | 9.99E-04 | 0.06 |
| LAI | 4 B | AX_109529745 | 1D | A/G | 374.00-374.00 | 2.98E-04 | 0.08 |
| LAI | 3 4 | AX_110607364 | 2A | G/C | 490.42-490.42 | 1.52E-05 | 0.11 |
| LAI | 3 B | Kukri_c24064_2095 | 2A | C/A | 606.83-606.83 | 4.35E-04 | 0.07 |
| LAI | 3 B | AX_94689584 | 3A | C/T | 9.84-15.57 | 3.15E-04 | 0.08 |
| LAI | 1 2 | Ku_c766_2292 | 4A | C/A | 27.67-27.67 | 4.49E-04 | 0.08 |
| LAI | 1 2 B | BobWhite_c7260_197 | 4A | C/T | 40.28-40.28 | 4.54E-04 | 0.07 |
| LAI | 1 B | BS00073404_51 | 5A | C/T | 448.09-448.09 | 8.19E-04 | 0.07 |
| LAI | 1 B | BobWhite_c10901_578 | 5A | G/A | 460.52-476.60 | 5.68E-05 | 0.10 |
| LAI | 1 4 B | TA006419-0680 | 5A | T/C | 504.78-504.88 | 4.10E-06 | 0.13 |
| LAI | 1 2 4 B | wsnp_Ex_c49211_53875600 | 5A | G/A | 510.22-511.88 | 4.50E-06 | 0.13 |
| LAI | 4 B | Excalibur_c49597_398 | 5A | G/A | 521.93-521.93 | 3.10E-04 | 0.08 |
| LAI | 4 B | BS00068178_51 | 5A | A/G | 540.05-540.06 | 1.37E-04 | 0.09 |
| LAI | 4 B | AX_109823316 | 5B | G/T | 389.82-391.05 | 6.67E-04 | 0.07 |
| LAI | 2 4 | AX_111540406 | 5B | A/G | 412.60-412.60 | 8.77E-04 | 0.07 |
| LAI | 1 B | BobWhite_c34650_141 | 5B | G/A | 427.25-433.53 | 1.51E-04 | 0.09 |
| LAI | 1 B | GENE-3440_199 | 5B | T/C | 442.41-445.45 | 1.14E-04 | 0.09 |
| LAI | 4 B | AX_108921249 | 5B | G/C | 578.59-580.70 | 1.19E-04 | 0.09 |
| LAI | 1 B | AX_94928994 | 5D | G/C | 362.89-368.33 | 6.03E-04 | 0.07 |
| LAI | 2 4 B | RAC875_c49940_385 | 5D | A/G | 399.29-399.29 | 8.03E-06 | 0.12 |
| LAI | 4 B | AX_109525732 | 6A | T/G | 3.69-6.74 | 4.21E-05 | 0.10 |
| LAI | 3 B | AX_111657916 | 6B | C/A | 691.64-692.79 | 3.49E-04 | 0.08 |
| LAI | 4 B | AX_109853929 | 6D | G/A | 371.30-371.30 | 7.12E-04 | 0.07 |
| LAI | 3 4 | AX_109279546 | 7A | T/C | 557.84-560.88 | 4.69E-04 | 0.07 |
| LAI | 1 B | wsnp_Ex_rep_c67660_66321934 | 7A | G/A | 567.58-567.58 | 3.99E-04 | 0.08 |
| LAI | 1 B | wsnp_CAP11_c1761_958064 | 7A | A/G | 589.24-589.24 | 1.37E-05 | 0.12 |
| LAI | 2 B | wsnp_Ex_c5177_9174930 | 7A | A/G | 623.45-625.64 | 2.12E-05 | 0.11 |
| LAI | 3 B | Tdurum_contig56175_791 | 7A | A/C | 641.22-641.44 | 1.47E-06 | 0.14 |
| LAI | 1 3 B | AX_94531094 | 7B | T/G | 601.32-601.32 | 1.98E-06 | 0.14 |
| LAI | 2 B | CAP7_c4608_228 | 7D | A/G | 540.86-540.86 | 6.03E-05 | 0.10 |
| GFR | 2 B | AX_109419477 | 1A | C/T | 355.72-356.43 | 3.67E-04 | 0.08 |
| GFR | 2 B | AX_110544900 | 1A | C/T | 462.80-463.25 | 3.99E-04 | 0.08 |
| GFR | 1 B | AX_111031300 | 1A | G/A | 532.92-544.57 | 7.64E-04 | 0.07 |
| GFR | 1 B | AX_95255804 | 1A | C/G | 572.25-574.48 | 3.01E-04 | 0.08 |
| GFR | 1 B | AX_109984121 | 1B | C/T | 91.00-93.63 | 6.43E-04 | 0.07 |
| GFR | 2 B | AX_111615790 | 1B | C/A | 222.29-222.29 | 3.14E-04 | 0.08 |
| GFR | 1 2 B | AX_110556416 | 1B | C/T | 555.63-560.49 | 3.12E-05 | 0.11 |
| GFR | 1 2 | AX_94521596 | 1B | G/T | 578.09-579.94 | 9.60E-04 | 0.07 |
| GFR | 2 4 | AX_95202607 | 1B | A/G | 686.93-688.28 | 8.86E-04 | 0.07 |
| GFR | 2 B | AX_94432917 | 2A | G/T | 504.55-512.87 | 1.19E-04 | 0.10 |
| GFR | 4 B | AX_108942936 | 2B | T/C | 30.27-30.27 | 5.57E-04 | 0.08 |
| GFR | 1 B | AX_94694655 | 2B | G/T | 708.23-708.23 | 6.36E-04 | 0.07 |
| GFR | 2 B | AX_110121961 | 2D | C/T | 36.08-36.08 | 7.48E-04 | 0.07 |
| GFR | 2 4 B | AX_111680512 | 2D | G/A | 553.75-568.25 | 5.88E-05 | 0.10 |
| GFR | 1 B | AX_108898813 | 3A | T/C | 7.44-12.87 | 7.28E-04 | 0.07 |
| GFR | 2 B | RAC875_c2464_274 | 3A | G/A | 56.67-56.68 | 4.04E-04 | 0.08 |
| GFR | 2 B | AX_94675903 | 3A | A/T | 562.19-562.19 | 8.53E-04 | 0.07 |
| GFR | 2 4 B | AX_111586409 | 3A | G/A | 646.13-649.03 | 6.53E-04 | 0.07 |
| GFR | 2 B | AX_108793242 | 3A | C/G | 718.39-727.55 | 6.22E-04 | 0.07 |
| GFR | 2 4 B | AX_111517177 | 3B | G/A | 672.78-672.78 | 7.22E-04 | 0.07 |
| GFR | 2 B | AX_108847577 | 3B | C/A | 771.77-772.40 | 4.14E-04 | 0.08 |
| GFR | 2 4 | AX_94693080 | 3D | A/G | 511.32-511.32 | 4.44E-04 | 0.08 |
| GFR | 2 B | AX_108915490 | 3D | T/C | 574.55-574.55 | 6.68E-04 | 0.07 |
| GFR | 1 2 B | AX_109016295 | 4A | T/C | 45.12-45.14 | 1.39E-04 | 0.09 |
| GFR | 4 B | AX_110541173 | 4A | A/G | 572.56-572.56 | 4.17E-04 | 0.08 |
| GFR | 2 B | AX_108964399 | 4A | G/A | 594.20-597.69 | 7.00E-04 | 0.07 |
| GFR | 1 B | AX_111092380 | 4A | C/G | 741.09-741.09 | 9.58E-04 | 0.07 |
| GFR | 2 B | AX_109554304 | 4B | T/C | 31.88-32.90 | 5.92E-04 | 0.08 |
| GFR | 2 B | AX_94957045 | 4B | C/T | 46.62-46.62 | 4.90E-04 | 0.08 |
| GFR | 1 2 4 B | AX_111585906 | 4B | C/T | 643.65-645.32 | 9.89E-05 | 0.10 |
| GFR | 1 2 B | AX_110046962 | 4D | G/A | 9.56-15.42 | 1.89E-04 | 0.09 |
| GFR | 2 B | AX_110057465 | 4D | C/G | 458.98-458.98 | 5.48E-04 | 0.08 |
| GFR | 2 B | AX_110499545 | 5A | A/G | 555.81-562.91 | 4.51E-04 | 0.08 |
| GFR | 2 B | AX_111781019 | 5A | C/T | 666.16-666.38 | 3.16E-04 | 0.08 |
| GFR | 3 4 | Ra_c11529_647 | 5A | G/A | 682.90-685.56 | 9.27E-05 | 0.10 |
| GFR | 2 4 B | AX_111108557 | 5B | A/G | 387.31-402.13 | 8.90E-05 | 0.10 |
| GFR | 1 2 3 B | AX_110079722 | 5B | T/C | 470.41-477.73 | 7.11E-07 | 0.16 |
| GFR | 1 2 B | AX_109586052 | 5B | C/T | 491.41-492.61 | 2.91E-04 | 0.08 |
| GFR | 1 B | AX_109383338 | 5B | C/T | 679.33-679.35 | 2.87E-04 | 0.08 |
| GFR | 2 4 B | AX_109292377 | 5D | C/T | 47.01-51.69 | 1.37E-04 | 0.09 |
| GFR | 1 B | AX_110957026 | 5D | G/A | 447.72-453.82 | 3.33E-04 | 0.08 |
| GFR | 1 2 B | AX_111533863 | 5D | C/T | 549.64-557.66 | 2.28E-04 | 0.09 |
| GFR | 1 2 B | AX_111477118 | 6A | C/A | 1.27-12.96 | 3.31E-05 | 0.11 |
| GFR | 2 B | AX_111062157 | 6A | C/T | 10.93-16.51 | 1.81E-04 | 0.09 |
| GFR | 2 B | AX_111228417 | 6B | A/G | 54.21-62.15 | 2.38E-04 | 0.09 |
| GFR | 1 B | AX_110968554 | 6B | C/G | 70.99-73.65 | 3.58E-04 | 0.08 |
| GFR | 1 2 B | Tdurum_contig41142_267 | 6B | G/T | 664.38-664.46 | 9.20E-05 | 0.10 |
| GFR | 2 B | AX_111167330 | 6B | G/A | 707.03-708.22 | 2.59E-04 | 0.09 |
| GFR | 1 B | AX_111645948 | 6D | C/T | 2.27-5.82 | 8.34E-04 | 0.07 |
| GFR | 1 B | Excalibur_c4615_1610 | 6D | A/G | 391.92-391.92 | 9.65E-04 | 0.07 |
| GFR | 3 4 B | BobWhite_c17095_237 | 7A | G/A | 610.68-613.73 | 9.62E-04 | 0.07 |
| GFR | 2 B | Kukri_c64387_218 | 7A | G/A | 670.77-680.33 | 1.50E-04 | 0.09 |
| GFR | 2 B | AX_110966047 | 7B | T/G | 191.06-197.00 | 3.64E-04 | 0.08 |
| GFR | 4 B | AX_108891047 | 7B | A/G | 663.31-664.08 | 8.63E-04 | 0.07 |
| GFR | 1 B | AX_111274023 | 7D | T/C | 3.41-4.01 | 9.00E-04 | 0.07 |
| GFR | 2 B | AX_111245063 | 7D | T/C | 88.65-88.65 | 5.18E-04 | 0.08 |
| GFR | 1 2 B | AX_110281782 | 7D | T/C | 135.93-136.95 | 4.24E-04 | 0.08 |
| GFR | 2 3 | AX_109353712 | 7D | A/G | 564.01-567.93 | 2.29E-04 | 0.09 |
| SGT | 3 B | AX_111455838 | 1B | T/A | 43.18-43.18 | 2.47E-04 | 0.09 |
| SGT | 3 B | AX_109816719 | 1D | G/A | 439.76-439.87 | 4.16E-04 | 0.08 |
| SGT | 4 B | BS00022393_51 | 2A | A/G | 5.93-5.93 | 1.56E-04 | 0.09 |
| SGT | 2 3 | BS00021739_51 | 2A | G/A | 79.75-79.75 | 7.93E-05 | 0.10 |
| SGT | 4 B | Kukri_c45015_145 | 2A | C/A | 117.18-117.18 | 8.07E-05 | 0.10 |
| SGT | 4 B | Excalibur_c11437_75 | 2A | A/G | 275.93-275.93 | 2.84E-04 | 0.08 |
| SGT | 4 B | RAC875_c18698_550 | 2A | A/G | 341.63-341.63 | 1.52E-04 | 0.09 |
| SGT | 4 B | BS00045521_51 | 2A | G/A | 543.31-543.31 | 6.43E-05 | 0.10 |
| SGT | 1 2 | Excalibur_c7282_285 | 2A | G/A | 710.10-710.10 | 5.02E-04 | 0.08 |
| SGT | 3 B | Kukri_c62467_362 | 3A | A/G | 53.34-54.88 | 1.15E-04 | 0.10 |
| SGT | 3 B | AX_111690680 | 3B | G/T | 51.96-51.96 | 2.94E-04 | 0.08 |
| SGT | 3 B | AX_94726771 | 3B | G/C | 720.19-720.19 | 5.87E-04 | 0.07 |
| SGT | 3 B | GENE_1674_631 | 3D | G/A | 42.09-42.09 | 2.65E-04 | 0.08 |
| SGT | 3 4 B | AX_108981706 | 4B | T/C | 643.31-647.67 | 4.52E-05 | 0.11 |
| SGT | 3 B | AX_111763778 | 5A | T/C | 670.54-670.58 | 4.43E-04 | 0.08 |
| SGT | 4 B | Tdurum_contig43092_876 | 6B | G/A | 41.99-41.99 | 8.43E-04 | 0.07 |
| SGT | 3 B | wsnp_Ku_c14603_22966714 | 6B | A/G | 613.58-613.58 | 8.73E-05 | 0.10 |
| SGT | 3 B | AX_94488714 | 7A | T/C | 129.87-129.87 | 2.95E-04 | 0.08 |
| SGT | 3 B | Kukri_c717_98 | 7A | G/A | 227.09-227.09 | 2.39E-04 | 0.09 |
| SGT | 1 B | AX_109007207 | 7A | G/A | 693.84-693.84 | 3.76E-04 | 0.08 |
| SGT | 1 4 | Excalibur_c25991_184 | 7B | G/A | 35.67-36.49 | 9.00E-06 | 0.12 |
| SGT | 4 B | Tdurum_contig11521_102 | 7B | G/A | 377.16-377.16 | 4.00E-04 | 0.08 |
| SGT | 3 B | AX_110675387 | 7B | G/A | 721.82-730.73 | 2.06E-05 | 0.12 |
| SGT | 3 B | AX_109716298 | 7D | G/A | 4.63-5.42 | 7.96E-04 | 0.07 |
| TTF | 2 3 B | Tdurum_contig43943_1165 | 1A | A/G | 10.07-10.07 | 3.57E-04 | 0.08 |
| TTF | 3 B | AX_111711661 | 1A | A/G | 531.72-531.87 | 5.79E-04 | 0.08 |
| TTF | 2 B | AX_95122826 | 1B | A/C | 100.65-100.65 | 9.25E-04 | 0.07 |
| TTF | 3 B | AX_110045506 | 1B | G/A | 561.77-563.32 | 4.04E-04 | 0.08 |
| TTF | 2 B | GENE_4120_155 | 1D | A/C | 7.04-7.04 | 9.60E-04 | 0.07 |
| TTF | 1 B | RAC875_c102059_75 | 1D | G/A | 34.62-42.14 | 1.55E-05 | 0.12 |
| TTF | 1 3 B | AX_89353533 | 1D | T/C | 469.06-469.06 | 1.96E-04 | 0.09 |
| TTF | 3 4 B | BS00022393_51 | 2A | A/G | 5.93-5.93 | 5.85E-04 | 0.08 |
| TTF | 2 B | AX_111037158 | 2A | T/C | 27.33-38.83 | 9.38E-05 | 0.10 |
| TTF | 1 B | AX_110467270 | 2A | T/G | 760.73-770.41 | 4.92E-05 | 0.11 |
| TTF | 3 B | AX_110368515 | 2B | A/G | 29.16-29.16 | 6.46E-04 | 0.08 |
| TTF | 2 3 B | AX_109510875 | 2B | A/G | 105.99-108.68 | 9.01E-05 | 0.10 |
| TTF | 3 B | Kukri_c36026_68 | 2B | A/G | 133.01-140.85 | 2.98E-05 | 0.11 |
| TTF | 3 B | AX_110102953 | 2B | C/T | 179.14-179.14 | 5.00E-04 | 0.08 |
| TTF | 4 B | wsnp_Ku_c2562_4879681 | 2B | G/A | 190.23-190.23 | 1.65E-04 | 0.09 |
| TTF | 2 4 B | AX_94863664 | 2B | G/A | 643.68-644.15 | 7.55E-05 | 0.10 |
| TTF | 2 B | wsnp_CAP12_c455_248396 | 2D | T/G | 14.78-14.78 | 5.00E-04 | 0.08 |
| TTF | 2 B | AX_110375481 | 2D | G/T | 539.88-539.88 | 8.97E-04 | 0.07 |
| TTF | 3 B | AX_110991030 | 2D | C/T | 646.27-646.42 | 3.13E-04 | 0.08 |
| TTF | 1 2 B | BS00004158_51 | 3A | G/A | 2.66-13.50 | 1.07E-04 | 0.10 |
| TTF | 2 3 B | AX_108780082 | 3A | G/A | 27.58-36.48 | 2.13E-05 | 0.12 |
| TTF | 2 B | AX_111712738 | 3A | C/G | 686.73-686.73 | 3.45E-04 | 0.08 |
| TTF | 3 B | AX_94557434 | 3A | G/T | 704.97-704.97 | 2.97E-04 | 0.08 |
| TTF | 3 B | AX_109442431 | 3B | A/T | 357.78-357.78 | 1.45E-04 | 0.09 |
| TTF | 2 B | AX_110450939 | 3B | T/C | 506.63-508.52 | 3.79E-04 | 0.08 |
| TTF | 2 3 4 B | GENE_1785_118 | 3B | A/G | 759.18-760.14 | 4.58E-05 | 0.11 |
| TTF | 1 2 | AX_108742757 | 3B | T/C | 786.96-786.96 | 6.52E-05 | 0.10 |
| TTF | 1 2 B | BS00004149_51 | 3D | A/G | 2.61-8.70 | 1.97E-04 | 0.09 |
| TTF | 2 B | AX_110480044 | 3D | T/C | 26.22-26.26 | 1.79E-04 | 0.09 |
| TTF | 3 B | AX_95257733 | 3D | A/G | 570.57-572.83 | 1.54E-04 | 0.09 |
| TTF | 4 B | AX_110914376 | 4A | T/C | 21.33-21.33 | 6.00E-04 | 0.08 |
| TTF | 3 B | wsnp_Ku_c3081_5776947 | 4A | G/A | 596.82-596.82 | 1.79E-04 | 0.09 |
| TTF | 2 B | Excalibur_c26895_128 | 4A | A/G | 619.28-619.28 | 4.47E-04 | 0.08 |
| TTF | 3 B | AX_86171513 | 4B | T/G | 599.81-604.20 | 6.55E-04 | 0.07 |
| TTF | 3 B | AX_110646801 | 4D | G/A | 98.57-98.57 | 9.56E-04 | 0.07 |
| TTF | 2 B | AX_108758715 | 5A | C/T | 666.24-666.31 | 5.50E-04 | 0.08 |
| TTF | 2 B | AX_89340428 | 5A | G/A | 684.94-684.94 | 8.40E-04 | 0.07 |
| TTF | 3 B | GENE_3574_643 | 5B | A/G | 519.15-519.15 | 1.69E-04 | 0.09 |
| TTF | 3 B | AX_109824320 | 5B | A/G | 562.96-573.81 | 3.32E-05 | 0.11 |
| TTF | 1 2 B | AX_109819352 | 5B | T/C | 584.1-584.11 | 6.72E-04 | 0.07 |
| TTF | 2 B | Kukri_c1214_2316 | 5B | A/G | 692.64-692.64 | 4.94E-04 | 0.08 |
| TTF | 1 4 B | AX_109870424 | 5D | T/C | 473.34-475.38 | 1.88E-04 | 0.09 |
| TTF | 2 B | wsnp_JD_c825_1223506 | 5D | A/C | 544.70-544.70 | 6.15E-04 | 0.08 |
| TTF | 3 4 B | BobWhite_s67148_292 | 6A | G/A | 581.89-582.94 | 3.71E-06 | 0.14 |
| TTF | 1 3 B | AX_110653284 | 6B | A/G | 692.78-692.78 | 7.60E-04 | 0.07 |
| TTF | 4 B | Tdurum_contig49186_437 | 7A | A/G | 35.40-35.40 | 8.44E-04 | 0.07 |
| TTF | 2 B | AX_94980988 | 7A | C/T | 101.86-101.86 | 6.62E-04 | 0.07 |
| TTF | 3 B | AX_109522657 | 7A | T/G | 543.07-545.94 | 2.35E-04 | 0.09 |
| TTF | 1 2 B | AX_109926350 | 7A | A/G | 563.15-566.02 | 9.95E-04 | 0.07 |
| TTF | 4 B | RAC875_c26328_75 | 7B | A/G | 35.67-36.46 | 4.90E-04 | 0.08 |
| TTF | 2 4 B | Tdurum_contig11028_236 | 7B | G/A | 47.62-58.73 | 2.13E-04 | 0.09 |
| TTF | 2 B | AX_108981342 | 7B | C/G | 629.43-629.43 | 6.85E-04 | 0.07 |
| TTF | 2 B | AX_110894345 | 7D | C/G | 24.16-24.16 | 2.32E-04 | 0.09 |
| TTM | 3 B | Excalibur_c9196_313 | 1A | A/G | 10.07-21.62 | 1.79E-04 | 0.09 |
| TTM | 2 B | AX_95258714 | 1A | C/T | 38.73-38.73 | 5.15E-04 | 0.08 |
| TTM | 4 B | AX_111039130 | 1B | T/C | 549.78-549.78 | 9.91E-04 | 0.07 |
| TTM | 2 B | AX_109929813 | 1D | G/C | 463.50-463.50 | 3.61E-04 | 0.08 |
| TTM | 1 2 B | AX_110467270 | 2A | T/G | 768.73-769.52 | 2.02E-04 | 0.09 |
| TTM | 3 4 B | BS00065994_51 | 2B | G/A | 132.58-140.85 | 1.90E-04 | 0.09 |
| TTM | 2 B | AX_94603670 | 2B | G/C | 184.13-184.13 | 3.21E-04 | 0.08 |
| TTM | 4 B | RFL_Contig4790_605 | 2B | A/G | 793.58-793.58 | 5.22E-04 | 0.08 |
| TTM | 4 B | AX_111574926 | 2D | G/A | 13.25-13.25 | 5.95E-04 | 0.07 |
| TTM | 2 4 B | AX_111120304 | 2D | C/A | 381.88-391.28 | 1.38E-04 | 0.09 |
| TTM | 2 B | AX_108780082 | 3A | G/A | 35.5-36.48 | 5.37E-04 | 0.08 |
| TTM | 1 4 B | AX_109880438 | 3A | T/C | 526.52-528.53 | 9.10E-04 | 0.07 |
| TTM | 4 B | AX_111064528 | 3B | T/C | 507.75-508.12 | 8.30E-04 | 0.07 |
| TTM | 2 B | AX_111145064 | 3B | G/A | 586.14-586.14 | 5.41E-04 | 0.08 |
| TTM | 1 2 B | AX_109843665 | 3B | A/C | 759.18-764.35 | 1.00E-04 | 0.10 |
| TTM | 1 2 B | AX_94434309 | 3D | G/T | 2.61-2.61 | 2.14E-04 | 0.09 |
| TTM | 1 2 B | BS00068556_51 | 3D | A/C | 164.22-164.22 | 7.79E-04 | 0.07 |
| TTM | 2 B | wsnp_Ku_c3081_5776947 | 4A | G/A | 596.82-596.82 | 5.03E-04 | 0.08 |
| TTM | 1 2 B | Ra_c36562_708 | 4B | A/C | 501.71-501.71 | 9.44E-05 | 0.10 |
| TTM | 2 B | AX_110164271 | 4D | G/A | 97.44-97.44 | 5.16E-04 | 0.08 |
| TTM | 4 B | AX_109745073 | 4D | A/G | 162.00-162.00 | 7.88E-04 | 0.07 |
| TTM | 2 B | wsnp_Ra_c2228_4309233 | 5A | G/A | 91.12-92.18 | 5.27E-04 | 0.08 |
| TTM | 1 2 | wsnp_Ex_c14812_22928900 | 5A | T/C | 383.88-383.88 | 2.27E-04 | 0.08 |
| TTM | 3 4 | Kukri_s112978_54 | 5A | A/G | 539.95-548.86 | 4.46E-05 | 0.11 |
| TTM | 4 B | AX_109824320 | 5B | A/G | 573.81-573.81 | 3.42E-04 | 0.08 |
| TTM | 3 4 B | IAAV5014 | 5B | A/G | 671.20-672.59 | 7.73E-04 | 0.07 |
| TTM | 3 B | AX_109902431 | 5B | A/G | 685.31-685.31 | 3.40E-04 | 0.08 |
| TTM | 1 3 B | AX_110371497 | 5D | G/A | 453.02-465.11 | 6.39E-05 | 0.10 |
| TTM | 4 B | Tdurum_contig10194_765 | 6A | T/G | 549.23-549.23 | 5.69E-04 | 0.08 |
| TTM | 1 B | AX_89316595 | 6B | C/A | 692.03-692.78 | 1.20E-04 | 0.09 |
| TTM | 2 B | AX_109408002 | 7A | T/C | 33.06-33.06 | 5.84E-04 | 0.07 |
| TTM | 1 B | AX_110995472 | 7A | A/G | 545.55-545.55 | 2.50E-04 | 0.09 |
| TTM | 1 B | AX_108737847 | 7A | C/T | 568.54-568.54 | 4.61E-04 | 0.08 |
| TTM | 1 B | BobWhite_c44404_312 | 7B | A/G | 17.66-17.66 | 9.29E-04 | 0.07 |
| SS | 1 B | AX_109989656 | 1A | C/T | 457.34-457.34 | 3.78E-04 | 0.07 |
| SS | 1 B | AX_109360552 | 1A | C/T | 473.63-479.71 | 3.83E-04 | 0.07 |
| SS | 1 B | AX_94586745 | 1A | G/A | 592.10-592.10 | 5.52E-05 | 0.09 |
| SS | 4 B | AX_108765328 | 1B | G/A | 39.62-39.62 | 1.53E-04 | 0.08 |
| SS | 1 B | AX_111094096 | 1B | G/A | 642.96-642.96 | 2.65E-04 | 0.08 |
| SS | 3 B | AX_108770672 | 1B | C/T | 686.92-686.92 | 5.02E-05 | 0.09 |
| SS | 1 B | AX_111735063 | 1D | C/G | 281.40-281.40 | 7.84E-05 | 0.09 |
| SS | 1 B | AX_111450701 | 2A | C/T | 759.49-759.49 | 9.88E-04 | 0.06 |
| SS | 2 B | AX_111634754 | 2B | C/T | 106.00-108.56 | 1.54E-04 | 0.08 |
| SS | 1 B | AX_111072441 | 2B | T/C | 200.56-200.57 | 5.71E-05 | 0.09 |
| SS | 3 B | AX_110035074 | 2D | C/T | 61.45-61.45 | 7.85E-04 | 0.06 |
| SS | 1 B | AX_109625085 | 2D | G/C | 79.99-80.82 | 4.59E-05 | 0.09 |
| SS | 2 B | AX_110500639 | 2D | A/G | 509.94-509.94 | 8.78E-04 | 0.06 |
| SS | 1 B | AX_111483435 | 3A | A/T | 25.36-25.36 | 5.09E-04 | 0.07 |
| SS | 4 B | AX_109296862 | 3A | G/C | 111.24-111.24 | 7.75E-05 | 0.09 |
| SS | 4 B | AX_110371489 | 3A | A/G | 119.46-126.31 | 4.93E-05 | 0.09 |
| SS | 1 B | AX_110926015 | 3A | A/G | 137.10-140.80 | 4.64E-04 | 0.07 |
| SS | 4 B | AX_109388257 | 3A | A/G | 245.60-245.60 | 5.68E-04 | 0.07 |
| SS | 3 B | AX_94594057 | 3B | A/C | 507.51-507.51 | 6.28E-04 | 0.07 |
| SS | 1 B | AX_111186153 | 4B | C/A | 657.25-657.25 | 3.70E-05 | 0.10 |
| SS | 1 B | AX_111115803 | 4D | A/C | 14.66-14.66 | 3.39E-04 | 0.07 |
| SS | 1 B | AX_110955910 | 4D | A/G | 389.52-389.52 | 4.86E-05 | 0.09 |
| SS | 1 2 B | AX_111710915 | 4D | T/C | 509.39-509.44 | 4.35E-07 | 0.15 |
| SS | 1 B | AX_108896311 | 5A | C/T | 3.52-3.52 | 2.05E-04 | 0.08 |
| SS | 1 B | AX_111189679 | 5A | A/G | 661.32-662.33 | 2.84E-04 | 0.07 |
| SS | 2 B | AX_110651786 | 5A | T/C | 684.94-685.79 | 1.48E-04 | 0.08 |
| SS | 1 2 B | AX_111477805 | 5A | G/A | 697.89-699.48 | 9.71E-07 | 0.14 |
| SS | 1 B | AX_111603341 | 5B | A/G | 43.90-43.90 | 2.52E-05 | 0.10 |
| SS | 1 B | AX_110984791 | 5B | C/T | 576.92-576.92 | 4.89E-04 | 0.07 |
| SS | 1 B | AX_111705875 | 5D | C/T | 155.84-155.84 | 3.60E-05 | 0.10 |
| SS | 2 B | AX_108993926 | 5D | C/T | 303.77-309.68 | 5.05E-04 | 0.07 |
| SS | 4 B | AX_109870424 | 5D | T/C | 473.34-473.34 | 2.91E-04 | 0.07 |
| SS | 1 B | AX_111481072 | 6A | T/A | 1.66-1.89 | 2.89E-04 | 0.07 |
| SS | 4 B | AX_94757955 | 6A | G/A | 611.86-611.86 | 6.74E-04 | 0.06 |
| SS | 1 B | AX_111591586 | 6D | A/G | 334.81-334.81 | 7.91E-05 | 0.09 |
| SS | 4 B | AX_109312992 | 6D | T/C | 465.21-465.96 | 2.12E-04 | 0.08 |
| SS | 1 B | IACX2471 | 7A | A/G | 669.73-669.73 | 1.97E-04 | 0.08 |
| SS | 1 B | AX_109580728 | 7B | T/A | 636.40-636.40 | 2.37E-04 | 0.08 |
| SS | 2 B | AX_110399029 | 7B | T/G | 715.85-715.85 | 8.38E-04 | 0.06 |
| LWC | 4 B | AX_95200832 | 1A | G/T | 10.25-10.25 | 8.61E-04 | 0.06 |
| LWC | 4 B | AX_95104798 | 1A | A/G | 551.98-551.98 | 1.04E-05 | 0.11 |
| LWC | 4 B | Excalibur_c88792_93 | 1A | T/C | 574.34-574.34 | 1.15E-05 | 0.11 |
| LWC | 4 B | AX_108824325 | 1B | T/C | 41.00-41.09 | 2.86E-04 | 0.08 |
| LWC | 3 B | AX_110387520 | 1B | C/A | 338.02-338.02 | 9.03E-04 | 0.06 |
| LWC | 4 B | AX_94653610 | 1B | G/C | 633.14-633.14 | 1.38E-05 | 0.11 |
| LWC | 4 B | AX_110922710 | 1D | G/A | 460.79-460.86 | 1.43E-05 | 0.11 |
| LWC | 1 B | AX_110507732 | 2B | A/G | 12.59-19.46 | 1.62E-04 | 0.08 |
| LWC | 4 B | AX_112287597 | 3A | C/T | 733.97-733.97 | 2.05E-04 | 0.08 |
| LWC | 4 B | AX_110567511 | 3D | A/G | 551.02-551.02 | 1.01E-04 | 0.09 |
| LWC | 4 B | AX_108760431 | 4B | A/G | 90.51-90.51 | 6.64E-04 | 0.07 |
| LWC | 4 B | GENE-2129_76 | 4B | G/A | 156.31-158.28 | 1.74E-04 | 0.08 |
| LWC | 4 B | Tdurum_contig57516_269 | 4B | G/T | 165.02-171.62 | 2.31E-04 | 0.08 |
| LWC | 4 B | IACX8023 | 4B | C/T | 405.37-405.37 | 2.59E-04 | 0.08 |
| LWC | 4 B | BS00065714_51 | 5A | G/A | 691.03-691.03 | 8.01E-04 | 0.06 |
| LWC | 4 B | AX_109966447 | 5B | G/C | 346.31-346.31 | 1.83E-04 | 0.08 |
| LWC | 4 B | Ku_c37271_957 | 5B | A/G | 483.00-483.00 | 4.38E-04 | 0.07 |
| LWC | 4 B | IACX5859 | 5B | G/C | 515.19-515.58 | 2.90E-04 | 0.08 |
| LWC | 4 B | RFL_Contig2304_1569 | 5B | G/A | 527.26-530.65 | 1.71E-04 | 0.08 |
| LWC | 4 B | tplb0027f13_452 | 5B | A/G | 546.83-546.83 | 6.18E-04 | 0.07 |
| LWC | 4 B | AX_109725811 | 5D | G/A | 440.35-447.78 | 2.42E-04 | 0.08 |
| LWC | 4 B | BS00051998_51 | 6B | A/G | 166.11-166.11 | 2.47E-04 | 0.08 |
| LWC | 4 B | AX_109474660 | 6B | C/T | 462.31-467.46 | 1.24E-04 | 0.09 |
| LWC | 4 B | GENE-4183_1109 | 6B | C/T | 606.43-606.43 | 7.13E-04 | 0.07 |
| LWC | 4 B | AX_111634483 | 7A | T/G | 484.53-484.53 | 8.95E-04 | 0.06 |
| LWC | 4 B | BobWhite_c5970_731 | 7B | A/G | 9.70-9.70 | 8.55E-04 | 0.06 |
| LWC | 3 4 B | CAP12_rep_c4929_51 | 7B | G/A | 450.64-451.49 | 7.72E-05 | 0.09 |
| LWC | 4 B | AX_110555929 | 7D | T/G | 61.35-61.35 | 2.96E-04 | 0.08 |
| SPWC | 4 B | AX_111834161 | 1A | T/G | 450.67-452.94 | 9.07E-04 | 0.07 |
| SPWC | 1 3 | AX_110563305 | 1A | C/G | 463.92-463.92 | 7.49E-05 | 0.10 |
| SPWC | 1 3 | AX_109371451 | 2A | G/T | 770.40-770.40 | 8.54E-04 | 0.07 |
| SPWC | 4 B | BS00093755_51 | 2B | A/G | 44.42-44.42 | 3.75E-04 | 0.08 |
| SPWC | 1 3 | AX_111688248 | 2B | T/A | 106.10-108.68 | 1.94E-04 | 0.09 |
| SPWC | 4 B | Kukri_c13830_601 | 3A | G/A | 14.85-21.79 | 2.49E-06 | 0.14 |
| SPWC | 1 3 | AX_109420398 | 3A | A/G | 750.59-750.59 | 2.51E-04 | 0.09 |
| SPWC | 4 B | IAAV1383 | 4A | A/C | 646.78-646.78 | 4.98E-04 | 0.08 |
| SPWC | 1 B | AX_94561875 | 4A | T/C | 659.13-661.65 | 2.48E-04 | 0.08 |
| SPWC | 1 B | AX_111001310 | 5A | G/C | 432.43-432.43 | 1.17E-04 | 0.09 |
| SPWC | 1 3 | AX_111763778 | 5A | T/C | 670.58-670.58 | 1.62E-04 | 0.09 |
| SPWC | 4 B | Ra_c11529_647 | 5A | G/A | 685.54-685.56 | 1.65E-04 | 0.09 |
| SPWC | 4 B | AX_109842839 | 5B | C/A | 491.5-491.81 | 3.47E-04 | 0.08 |
| SPWC | 4 B | AX_109081425 | 5B | C/T | 494.79-502.26 | 4.03E-05 | 0.11 |
| SPWC | 4 B | AX_108987749 | 6A | G/C | 22.70-22.70 | 7.98E-04 | 0.07 |
| SPWC | 4 B | AX_110436320 | 6B | C/T | 454.04-454.04 | 8.13E-04 | 0.07 |
| SPWC | 4 B | AX_111583351 | 6B | G/A | 462.31-469.07 | 2.99E-04 | 0.08 |
| SPWC | 4 B | AX_110961512 | 7A | C/T | 34.72-34.75 | 5.73E-04 | 0.07 |
| SPWC | 1 B | AX_110581816 | 7A | A/C | 48.06-48.74 | 2.41E-05 | 0.11 |
| SPWC | 1 3 B | RAC875_c50676_588 | 7A | T/G | 725.93-725.93 | 3.40E-05 | 0.11 |
| SPWC | 1 3 B | AX_109511112 | 7B | T/C | 133.50-133.74 | 8.07E-05 | 0.10 |
| SPWC | 1 B | Excalibur_rep_c105595_185 | 7B | A/G | 732.65-732.65 | 9.22E-05 | 0.10 |
| SPWC | 4 B | AX_111568850 | 7D | A/G | 71.59-76.94 | 4.44E-05 | 0.11 |
| STWC | 3 B | AX_108827772 | 2D | C/T | 633.61-633.61 | 6.59E-04 | 0.07 |
| STWC | 1 2 | AX_110959791 | 5A | G/A | 11.15-11.23 | 2.42E-04 | 0.08 |
| STWC | 1 2 | Tdurum_contig17318_413 | 6A | A/C | 404.70-404.70 | 3.55E-05 | 0.11 |
| STWC | 1 2 | AX_109320292 | 6B | C/T | 445.01-461.95 | 3.35E-05 | 0.11 |
| STWC | 1 2 | AX_109828154 | 7A | A/G | 444.00-444.00 | 5.28E-04 | 0.08 |
| STWC | 3 B | RAC875_c50676_588 | 7A | T/G | 725.93-725.93 | 3.40E-05 | 0.11 |
| LDWS | 1 2 B | AX_94778726 | 1A | C/T | 530.17-530.17 | 1.22E-04 | 0.09 |
| LDWS | 1 3 B | AX_95104798 | 1A | A/G | 551.98-551.98 | 2.96E-04 | 0.08 |
| LDWS | 2 4 B | AX_109984121 | 1B | C/T | 91.00-93.63 | 3.25E-05 | 0.11 |
| LDWS | 2 4 B | AX_111615790 | 1B | C/A | 222.29-222.29 | 4.11E-04 | 0.07 |
| LDWS | 2 B | AX_110387520 | 1B | C/A | 334.55-339.58 | 3.71E-05 | 0.10 |
| LDWS | 2 B | AX_110038927 | 1B | C/T | 348.03-348.03 | 1.96E-04 | 0.08 |
| LDWS | 1 2 B | AX_112287814 | 1B | C/T | 582.91-582.91 | 1.37E-04 | 0.09 |
| LDWS | 2 4 B | TA001769-0538 | 1B | T/C | 633.14-633.33 | 1.81E-04 | 0.08 |
| LDWS | 3 4 B | AX_110941446 | 1B | T/C | 648.03-648.03 | 9.64E-04 | 0.06 |
| LDWS | 4 B | AX_111011205 | 1B | C/G | 681.11-681.11 | 9.12E-04 | 0.07 |
| LDWS | 1 2 3 4 B | Ra_c42714_1137 | 2A | A/G | 605.80-605.80 | 3.55E-06 | 0.13 |
| LDWS | 2 B | AX_110913236 | 2A | A/G | 676.23-677.37 | 5.88E-04 | 0.07 |
| LDWS | 4 B | AX_109841008 | 2A | C/T | 742.72-746.75 | 4.79E-04 | 0.07 |
| LDWS | 4 B | AX_111453952 | 2A | A/G | 761.05-761.05 | 6.42E-04 | 0.07 |
| LDWS | 2 4 B | AX_108942936 | 2B | T/C | 30.27-30.27 | 3.08E-04 | 0.08 |
| LDWS | 4 B | RAC875_c45600_228 | 2B | G/A | 339.17-339.17 | 9.43E-04 | 0.07 |
| LDWS | 2 B | AX_109490219 | 2B | A/G | 572.32-572.32 | 8.45E-04 | 0.07 |
| LDWS | 2 3 B | AX_111235991 | 2B | A/C | 619.14-626.54 | 6.42E-04 | 0.07 |
| LDWS | 4 B | GENE_0675_104 | 2B | A/G | 647.11-647.11 | 8.30E-04 | 0.07 |
| LDWS | 1 B | AX_94569403 | 2D | T/C | 461.30-461.30 | 6.61E-04 | 0.07 |
| LDWS | 4 B | AX_111680512 | 2D | G/A | 566.16-566.16 | 8.58E-04 | 0.07 |
| LDWS | 4 B | AX_108898813 | 3A | T/C | 10.16-10.17 | 2.10E-04 | 0.08 |
| LDWS | 2 4 B | AX_109890813 | 3A | T/C | 647.36-649.6 | 1.20E-04 | 0.09 |
| LDWS | 1 3 B | AX_110474783 | 3A | G/C | 721.65-731.73 | 3.76E-05 | 0.10 |
| LDWS | 4 B | AX_111511076 | 3B | G/A | 26.76-27.37 | 4.46E-04 | 0.07 |
| LDWS | 2 4 B | Excalibur_c35645_587 | 3B | A/C | 43.28-43.28 | 5.34E-05 | 0.10 |
| LDWS | 2 4 B | AX_111452231 | 3B | G/A | 98.30-98.30 | 5.51E-04 | 0.07 |
| LDWS | 4 B | BS00091643_51 | 3B | A/G | 603.45-603.45 | 9.38E-04 | 0.07 |
| LDWS | 2 4 B | AX_109917617 | 3B | T/G | 739.91-744.35 | 3.61E-04 | 0.08 |
| LDWS | 2 4 B | AX_109270894 | 3D | C/A | 11.42-11.42 | 1.76E-04 | 0.08 |
| LDWS | 4 B | AX_94693080 | 3D | A/G | 511.32-511.32 | 9.31E-04 | 0.07 |
| LDWS | 2 B | AX_110567511 | 3D | A/G | 551.02-551.02 | 3.87E-04 | 0.08 |
| LDWS | 4 B | AX_110541173 | 4A | A/G | 572.56-572.56 | 5.23E-04 | 0.07 |
| LDWS | 2 4 | AX_111633660 | 4A | G/A | 618.86-619.39 | 4.27E-04 | 0.07 |
| LDWS | 2 B | AX_110512399 | 4B | A/C | 16.80-16.80 | 4.36E-04 | 0.07 |
| LDWS | 2 4 B | AX_89708030 | 4B | G/A | 28.95-37.87 | 1.55E-05 | 0.11 |
| LDWS | 2 B | AX_94957045 | 4B | C/T | 46.62-46.62 | 5.51E-04 | 0.07 |
| LDWS | 2 B | AX_109399379 | 4B | C/A | 649.53-653.21 | 1.22E-04 | 0.09 |
| LDWS | 2 B | AX_94943082 | 4D | G/A | 15.42-24.34 | 1.77E-04 | 0.08 |
| LDWS | 3 4 | tplb0057m23_1318 | 5A | A/G | 475.47-475.47 | 3.69E-05 | 0.10 |
| LDWS | 3 B | AX_94456702 | 5A | C/T | 706.93-706.93 | 6.42E-04 | 0.07 |
| LDWS | 1 2 4 B | AX_111109252 | 5B | A/G | 387.31-394.74 | 1.37E-04 | 0.09 |
| LDWS | 2 4 B | AX_111486576 | 5B | G/T | 445.95-445.95 | 2.63E-06 | 0.14 |
| LDWS | 2 B | AX_110910348 | 5B | C/T | 460.27-460.27 | 9.89E-04 | 0.06 |
| LDWS | 2 4 B | AX_108820026 | 5B | T/C | 470.41-477.73 | 4.94E-06 | 0.13 |
| LDWS | 4 B | AX_108932230 | 5B | G/T | 492.00-492.78 | 2.14E-04 | 0.08 |
| LDWS | 3 4 B | BS00029540_51 | 5B | A/C | 660.37-660.37 | 9.98E-04 | 0.06 |
| LDWS | 2 4 B | AX_110538449 | 5B | T/G | 689.93-692.85 | 3.56E-04 | 0.08 |
| LDWS | 2 3 4 B | AX_110464564 | 5D | C/T | 46.85-57.69 | 9.00E-07 | 0.15 |
| LDWS | 1 4 B | AX_94388289 | 5D | C/G | 319.22-335.24 | 3.80E-04 | 0.08 |
| LDWS | 2 B | AX_94976637 | 5D | A/G | 371.27-371.27 | 1.47E-04 | 0.09 |
| LDWS | 2 B | AX_108964976 | 5D | A/G | 547.83-556.55 | 1.58E-04 | 0.09 |
| LDWS | 2 B | AX_94585210 | 6A | C/T | 5.88-11.57 | 5.27E-04 | 0.07 |
| LDWS | 2 4 B | GENE_3980_221 | 6A | G/A | 570.38-570.38 | 2.95E-04 | 0.08 |
| LDWS | 2 B | AX_108746331 | 6A | C/T | 613.66-614.81 | 3.23E-04 | 0.08 |
| LDWS | 4 B | AX_94626844 | 6B | C/T | 233.03-233.03 | 3.93E-04 | 0.08 |
| LDWS | 2 B | AX_109956242 | 6B | A/G | 362.57-362.57 | 9.20E-04 | 0.07 |
| LDWS | 2 4 B | GENE-3980_654 | 6B | T/C | 640.9-641.20 | 4.11E-05 | 0.10 |
| LDWS | 2 B | AX_111645948 | 6D | C/T | 5.82-5.82 | 2.32E-04 | 0.08 |
| LDWS | 4 B | Excalibur_c4615_1610 | 6D | A/G | 391.92-391.92 | 4.21E-04 | 0.07 |
| LDWS | 3 B | AX_111548564 | 6D | C/T | 471.15-471.5 | 6.75E-04 | 0.07 |
| LDWS | 2 B | AX_109285046 | 7A | C/A | 12.91-12.91 | 9.36E-04 | 0.07 |
| LDWS | 2 4 B | AX_111486899 | 7A | G/C | 191.86-191.86 | 7.35E-04 | 0.07 |
| LDWS | 1 B | AX_111529243 | 7A | C/T | 486.15-487.85 | 4.16E-04 | 0.07 |
| LDWS | 2 4 B | BobWhite_c17095_237 | 7A | G/A | 611.63-613.73 | 2.95E-05 | 0.11 |
| LDWS | 4 B | AX_111659426 | 7A | A/G | 696.73-696.76 | 7.69E-04 | 0.07 |
| LDWS | 2 B | AX_110095270 | 7D | C/T | 34.14-34.14 | 9.04E-04 | 0.07 |
| LDWS | 2 4 B | AX_111132781 | 7D | C/A | 129.97-137.01 | 1.13E-04 | 0.09 |
| SPDWS | 2 4 B | AX_109493015 | 1A | A/G | 493.92-497.34 | 1.82E-04 | 0.09 |
| SPDWS | 3 B | AX_110954897 | 1A | C/G | 567.37-572.35 | 3.49E-04 | 0.08 |
| SPDWS | 4 B | AX_111615790 | 1B | C/A | 222.29-222.29 | 2.30E-04 | 0.08 |
| SPDWS | 4 B | RAC875_rep_c106876_558 | 1B | A/G | 573.57-573.57 | 5.00E-04 | 0.07 |
| SPDWS | 2 B | BS00110586_51 | 2A | A/G | 676.23-676.25 | 9.50E-04 | 0.07 |
| SPDWS | 4 B | AX_110495113 | 2B | A/C | 13.88-13.88 | 6.93E-04 | 0.07 |
| SPDWS | 2 4 B | CAP8_c3982_23 | 2B | A/G | 202.74-210.65 | 1.38E-04 | 0.09 |
| SPDWS | 4 B | RAC875_c45600_228 | 2B | G/A | 339.17-339.17 | 3.49E-04 | 0.08 |
| SPDWS | 4 B | RAC875_c27297_2153 | 2B | G/A | 385.19-385.19 | 9.58E-05 | 0.09 |
| SPDWS | 4 B | wsnp_BF473744B_Ta_2_2 | 2B | C/A | 648.08-648.08 | 8.54E-04 | 0.07 |
| SPDWS | 2 B | AX_110121961 | 2D | C/T | 36.08-36.08 | 9.94E-04 | 0.07 |
| SPDWS | 2 4 B | AX_111382753 | 2D | G/T | 89.02-89.02 | 5.45E-05 | 0.10 |
| SPDWS | 2 3 4 B | AX_111705751 | 2D | G/A | 96.58-110.05 | 1.76E-05 | 0.11 |
| SPDWS | 2 4 B | AX_111169886 | 2D | T/C | 119.83-119.83 | 1.87E-04 | 0.08 |
| SPDWS | 2 4 B | AX_111287239 | 2D | C/T | 153.06-164.36 | 5.85E-04 | 0.07 |
| SPDWS | 2 4 | AX_111797841 | 2D | G/A | 197.84-197.84 | 7.24E-04 | 0.07 |
| SPDWS | 4 B | AX_110694091 | 2D | G/A | 240.37-240.37 | 6.24E-04 | 0.07 |
| SPDWS | 4 B | AX_111830766 | 2D | G/A | 263.39-263.39 | 6.49E-04 | 0.07 |
| SPDWS | 2 4 B | AX_111260957 | 2D | C/T | 358.46-358.46 | 1.32E-04 | 0.09 |
| SPDWS | 4 B | AX_111838609 | 2D | C/T | 378.91-378.91 | 6.24E-04 | 0.07 |
| SPDWS | 4 B | AX_111555631 | 2D | C/T | 553.75-553.75 | 8.47E-04 | 0.07 |
| SPDWS | 3 4 B | AX_108744914 | 2D | C/G | 566.16-573.38 | 2.09E-05 | 0.11 |
| SPDWS | 4 B | AX_111079825 | 3A | G/A | 10.16-10.16 | 2.48E-04 | 0.08 |
| SPDWS | 4 B | AX_109384914 | 3A | A/C | 647.36-654.32 | 2.79E-04 | 0.08 |
| SPDWS | 1 4 B | AX_110613078 | 3A | T/G | 724.54-731.73 | 2.83E-04 | 0.08 |
| SPDWS | 4 B | AX_94602255 | 3B | C/T | 6.38-6.38 | 4.14E-04 | 0.08 |
| SPDWS | 4 B | AX_111511076 | 3B | G/A | 26.76-26.79 | 2.67E-04 | 0.08 |
| SPDWS | 4 B | wsnp_Ex_c3130_5790163 | 3B | G/A | 40.65-40.65 | 6.37E-04 | 0.07 |
| SPDWS | 4 B | BS00091643_51 | 3B | A/G | 602.36-603.45 | 3.05E-04 | 0.08 |
| SPDWS | 2 4 B | Excalibur_c48047_90 | 3B | G/A | 617.68-617.68 | 3.54E-04 | 0.08 |
| SPDWS | 2 B | AX_94692319 | 3B | G/A | 636.45-636.45 | 3.60E-04 | 0.08 |
| SPDWS | 4 B | AX_109318897 | 3B | C/T | 744.60-744.60 | 9.95E-04 | 0.07 |
| SPDWS | 3 B | AX_108847577 | 3B | C/A | 771.53-773.05 | 2.72E-04 | 0.08 |
| SPDWS | 1 3 B | AX_108862674 | 3B | T/C | 810.33-810.57 | 4.32E-04 | 0.07 |
| SPDWS | 4 B | IACX7741 | 3D | G/A | 455.87-456.63 | 3.37E-04 | 0.08 |
| SPDWS | 4 B | AX_94693080 | 3D | A/G | 511.32-511.32 | 7.61E-04 | 0.07 |
| SPDWS | 2 3 B | AX_111640784 | 4B | C/T | 12.09-12.51 | 9.04E-05 | 0.09 |
| SPDWS | 4 B | AX_89323611 | 4B | A/G | 32.90-32.90 | 1.26E-04 | 0.09 |
| SPDWS | 3 B | Tdurum_contig42366_893 | 4B | C/A | 456.36-456.36 | 7.65E-04 | 0.07 |
| SPDWS | 3 4 B | AX_95084501 | 4B | T/C | 653.32-653.32 | 4.13E-04 | 0.08 |
| SPDWS | 2 4 B | AX_109987236 | 4D | G/A | 47.32-47.32 | 7.62E-05 | 0.10 |
| SPDWS | 2 4 B | BobWhite_c10901_578 | 5A | G/A | 454.83-476.67 | 1.98E-04 | 0.08 |
| SPDWS | 2 4 B | RAC875_c46811_128 | 5A | G/A | 663.28-663.28 | 4.89E-05 | 0.10 |
| SPDWS | 2 4 B | AX_109487407 | 5B | T/C | 387.31-394.25 | 6.30E-04 | 0.07 |
| SPDWS | 4 B | AX_110979631 | 5B | G/A | 402.84-403.18 | 8.17E-04 | 0.07 |
| SPDWS | 2 4 B | AX_108995332 | 5B | T/A | 427.25-430.74 | 2.42E-04 | 0.08 |
| SPDWS | 2 4 B | AX_110573299 | 5B | A/G | 439.06-477.68 | 2.94E-05 | 0.11 |
| SPDWS | 4 B | AX_111183518 | 5B | A/G | 470.41-477.68 | 5.84E-06 | 0.13 |
| SPDWS | 4 B | AX_110498161 | 5B | A/G | 491.50-492.79 | 7.77E-06 | 0.12 |
| SPDWS | 2 4 | wsnp_Ex_rep_c67783_66469848 | 5B | G/A | 679.35-679.35 | 9.83E-04 | 0.07 |
| SPDWS | 2 3 4 B | AX_110914088 | 5B | C/T | 692.13-692.13 | 4.80E-05 | 0.10 |
| SPDWS | 2 4 B | AX_110464564 | 5D | C/T | 49.18-49.18 | 2.62E-06 | 0.14 |
| SPDWS | 2 B | AX_95200547 | 5D | T/A | 373.77-373.77 | 7.18E-04 | 0.07 |
| SPDWS | 2 4 B | AX_94524902 | 5D | A/C | 547.5-557.66 | 2.59E-06 | 0.14 |
| SPDWS | 2 B | AX_111480329 | 6A | G/A | 5.21-12.41 | 4.86E-04 | 0.07 |
| SPDWS | 2 4 B | BS00063479_51 | 6A | A/G | 256.75-256.75 | 3.25E-04 | 0.08 |
| SPDWS | 4 B | Tdurum_contig12123_1650 | 6A | G/A | 611.56-611.56 | 5.17E-04 | 0.07 |
| SPDWS | 4 B | AX_110910168 | 6B | G/C | 113.51-117.52 | 8.67E-05 | 0.09 |
| SPDWS | 4 B | Tdurum_contig41142_267 | 6B | G/T | 664.46-664.46 | 9.92E-04 | 0.07 |
| SPDWS | 2 B | AX_110130742 | 6B | G/A | 708.19-709.53 | 5.03E-04 | 0.07 |
| SPDWS | 2 4 B | AX_94965830 | 6D | C/T | 5.57-5.57 | 7.28E-05 | 0.10 |
| SPDWS | 2 4 | Excalibur_c4615_1610 | 6D | A/G | 391.92-391.92 | 9.85E-05 | 0.10 |
| SPDWS | 4 B | wsnp_CAP11_c1761_958064 | 7A | A/G | 589.24-589.24 | 5.89E-04 | 0.07 |
| SPDWS | 4 B | AX_111008688 | 7A | A/G | 611.63-615.37 | 1.97E-05 | 0.11 |
| SPDWS | 3 B | wsnp_Ex_c7071_12171619 | 7A | G/A | 680.33-680.33 | 4.66E-04 | 0.07 |
| SPDWS | 3 4 B | AX_111046029 | 7D | A/T | 75.92-89.54 | 4.70E-04 | 0.07 |
| SPDWS | 4 B | AX_111634150 | 7D | G/A | 127.76-137.92 | 1.24E-04 | 0.09 |
| STDWS | 2 4 B | AX_110040067 | 1A | G/A | 127.93-127.93 | 3.36E-04 | 0.08 |
| STDWS | 2 B | AX_110998172 | 1A | G/A | 242.01-253.26 | 2.00E-04 | 0.09 |
| STDWS | 2 4 B | AX_111509016 | 1A | T/C | 352.26-356.43 | 1.60E-04 | 0.09 |
| STDWS | 4 B | AX_109493015 | 1A | A/G | 494.75-495.60 | 6.28E-04 | 0.07 |
| STDWS | 2 B | AX_94778726 | 1A | C/T | 530.17-530.17 | 4.61E-04 | 0.08 |
| STDWS | 3 B | AX_110954897 | 1A | C/G | 572.34-572.35 | 8.43E-04 | 0.07 |
| STDWS | 2 3 4 B | AX_109984121 | 1B | C/T | 91.00-93.63 | 5.51E-05 | 0.10 |
| STDWS | 2 3 4 B | AX_111615790 | 1B | C/A | 222.29-222.29 | 3.21E-04 | 0.08 |
| STDWS | 2 B | AX_112287814 | 1B | C/T | 582.91-582.91 | 5.42E-04 | 0.07 |
| STDWS | 2 B | BobWhite_c16206_317 | 1B | G/A | 603.22-603.22 | 9.30E-04 | 0.07 |
| STDWS | 2 3 4 B | TA001769-0538 | 1B | T/C | 622.57-633.33 | 4.04E-05 | 0.11 |
| STDWS | 3 B | AX_110941446 | 1B | T/C | 648.03-650.49 | 4.37E-04 | 0.08 |
| STDWS | 3 4 | BS00075524_51 | 2A | A/G | 42.39-47.92 | 8.81E-05 | 0.10 |
| STDWS | 2 3 4 B | Ra_c42714_1137 | 2A | A/G | 605.80-605.80 | 5.71E-06 | 0.13 |
| STDWS | 2 4 B | AX_109841008 | 2A | C/T | 746.75-746.75 | 1.17E-04 | 0.09 |
| STDWS | 3 4 B | AX_108942936 | 2B | T/C | 30.27-30.27 | 4.47E-04 | 0.08 |
| STDWS | 2 3 4 B | RAC875_c45600_228 | 2B | G/A | 339.17-339.17 | 2.57E-04 | 0.08 |
| STDWS | 2 3 4 B | RAC875_c27297_2153 | 2B | G/A | 385.19-385.19 | 2.17E-04 | 0.09 |
| STDWS | 3 B | AX_95186628 | 2B | T/C | 599.28-599.28 | 7.60E-04 | 0.07 |
| STDWS | 2 3 4 B | wsnp_BF473744B_Ta_2_2 | 2B | C/A | 648.08-648.08 | 4.88E-04 | 0.08 |
| STDWS | 2 4 B | Ra_c19225_591 | 2B | A/G | 748.97-748.97 | 4.76E-05 | 0.10 |
| STDWS | 2 4 B | AX_110121961 | 2D | C/T | 36.08-36.08 | 2.79E-04 | 0.08 |
| STDWS | 2 3 4 B | AX_111079825 | 3A | G/A | 10.16-10.16 | 5.08E-05 | 0.10 |
| STDWS | 2 3 4 B | Kukri_c10210_1387 | 3A | G/A | 648.87-648.87 | 5.61E-05 | 0.10 |
| STDWS | 3 4 B | AX_109580486 | 3A | C/A | 718.75-718.75 | 2.97E-04 | 0.08 |
| STDWS | 3 4 B | AX_108833035 | 3A | C/A | 725.11-731.46 | 1.63E-04 | 0.09 |
| STDWS | 2 3 4 B | AX_94602255 | 3B | C/T | 6.38-6.38 | 2.93E-04 | 0.08 |
| STDWS | 2 3 4 B | AX_111511076 | 3B | G/A | 26.76-26.76 | 1.75E-04 | 0.09 |
| STDWS | 2 B | Excalibur_c35645_587 | 3B | A/C | 43.28-43.28 | 5.55E-04 | 0.07 |
| STDWS | 4 B | BS00091643_51 | 3B | A/G | 603.45-603.45 | 6.44E-04 | 0.07 |
| STDWS | 2 4 B | AX_111231933 | 3B | C/A | 742.25-742.25 | 3.99E-04 | 0.08 |
| STDWS | 2 4 | AX_89576720 | 3D | C/T | 481.57-481.57 | 5.10E-05 | 0.11 |
| STDWS | 3 B | AX_94693080 | 3D | A/G | 511.32-511.32 | 8.38E-04 | 0.07 |
| STDWS | 2 B | AX_95107898 | 3D | A/G | 549.91-551.02 | 6.46E-04 | 0.07 |
| STDWS | 4 B | AX_110541173 | 4A | A/G | 572.56-572.56 | 6.76E-04 | 0.07 |
| STDWS | 3 B | AX_110649024 | 4B | C/T | 12.09-14.12 | 2.09E-04 | 0.09 |
| STDWS | 2 3 4 B | AX_89323611 | 4B | A/G | 32.25-37.87 | 2.55E-06 | 0.14 |
| STDWS | 2 3 B | AX_94957045 | 4B | C/T | 42.05-46.62 | 2.66E-04 | 0.08 |
| STDWS | 2 B | AX_109283891 | 4B | A/C | 643.65-644.83 | 3.66E-04 | 0.08 |
| STDWS | 3 B | AX_109399379 | 4B | C/A | 649.47-653.21 | 4.87E-04 | 0.08 |
| STDWS | 4 B | AX_111979632 | 4D | C/T | 7.83-24.34 | 1.26E-04 | 0.09 |
| STDWS | 3 4 | wsnp_Ex_rep_c67292_65834396 | 5A | G/A | 456.61-456.61 | 5.85E-04 | 0.07 |
| STDWS | 2 4 B | AX_108877126 | 5A | C/T | 471.69-476.67 | 2.44E-04 | 0.08 |
| STDWS | 4 B | RAC875_c46811_128 | 5A | G/A | 663.28-663.28 | 7.90E-04 | 0.07 |
| STDWS | 4 B | GENE-2826_325 | 5A | A/C | 688.32-688.32 | 3.51E-04 | 0.08 |
| STDWS | 3 B | AX_94456702 | 5A | C/T | 706.93-706.93 | 1.18E-04 | 0.09 |
| STDWS | 4 B | AX_108774619 | 5B | C/T | 387.31-394.74 | 3.18E-04 | 0.08 |
| STDWS | 4 B | AX_108995332 | 5B | T/A | 427.25-427.82 | 6.90E-04 | 0.07 |
| STDWS | 2 4 B | AX_111486576 | 5B | G/T | 439.25-447.45 | 1.61E-06 | 0.15 |
| STDWS | 1 4 B | AX_108820026 | 5B | T/C | 470.41-477.73 | 1.73E-07 | 0.18 |
| STDWS | 2 4 B | AX_108745156 | 5B | T/C | 491.41-492.79 | 8.37E-05 | 0.10 |
| STDWS | 2 B | AX_94467817 | 5B | A/C | 595.66-595.66 | 5.97E-04 | 0.07 |
| STDWS | 3 4 B | BS00029540_51 | 5B | A/C | 660.37-660.37 | 4.63E-04 | 0.08 |
| STDWS | 2 4 B | AX_110464564 | 5D | C/T | 46.85-57.69 | 1.70E-06 | 0.15 |
| STDWS | 4 B | AX_94505455 | 5D | C/T | 335.24-335.24 | 8.22E-04 | 0.07 |
| STDWS | 4 B | AX_94976637 | 5D | A/G | 371.27-372.30 | 1.01E-05 | 0.12 |
| STDWS | 3 4 | AX_94524902 | 5D | A/C | 547.5-556.72 | 4.16E-04 | 0.08 |
| STDWS | 2 4 B | AX_108760795 | 6A | C/A | 5.21-12.96 | 3.20E-05 | 0.11 |
| STDWS | 3 4 B | wsnp_Ex_c22089_31270755 | 6A | A/G | 64.32-65.07 | 5.63E-04 | 0.07 |
| STDWS | 2 4 | AX_109895078 | 6B | C/T | 113.77-115.95 | 4.48E-04 | 0.08 |
| STDWS | 2 4 | AX_110395789 | 6B | G/T | 125.41-125.41 | 6.38E-04 | 0.07 |
| STDWS | 2 4 B | AX_108941919 | 6B | A/G | 487.55-487.55 | 4.70E-04 | 0.08 |
| STDWS | 2 3 4 B | GENE-3980_654 | 6B | T/C | 640.9-643.79 | 8.01E-05 | 0.10 |
| STDWS | 3 4 B | Tdurum_contig41142_267 | 6B | G/T | 664.27-664.46 | 5.72E-05 | 0.10 |
| STDWS | 2 B | AX_110992705 | 6B | G/A | 704.86-708.19 | 4.69E-04 | 0.08 |
| STDWS | 2 B | Kukri_c48283_78 | 6B | G/A | 716.26-717.86 | 5.39E-04 | 0.07 |
| STDWS | 2 4 B | AX_94965830 | 6D | C/T | 5.57-5.82 | 2.77E-05 | 0.11 |
| STDWS | 2 4 B | Excalibur_c4615_1610 | 6D | A/G | 391.92-391.92 | 8.56E-05 | 0.10 |
| STDWS | 1 4 B | AX_111529243 | 7A | C/T | 487.85-487.85 | 5.42E-04 | 0.07 |
| STDWS | 3 4 B | wsnp_CAP11_c1761_958064 | 7A | A/G | 589.24-589.24 | 9.51E-04 | 0.07 |
| STDWS | 4 B | BobWhite_c17095_237 | 7A | G/A | 611.63-613.73 | 3.17E-06 | 0.14 |
| STDWS | 1 4 | AX_110044150 | 7A | A/G | 623.45-623.45 | 1.53E-04 | 0.09 |
| STDWS | 2 B | AX_111132781 | 7D | C/A | 129.97-129.97 | 7.89E-04 | 0.07 |
| TDWS | 4 B | AX_109419477 | 1A | C/T | 352.85-356.43 | 2.76E-04 | 0.08 |
| TDWS | 4 B | AX_109493015 | 1A | A/G | 494.45-495.71 | 3.37E-04 | 0.08 |
| TDWS | 3 B | AX_110954897 | 1A | C/G | 572.24-572.35 | 4.86E-04 | 0.07 |
| TDWS | 2 4 B | AX_109984121 | 1B | C/T | 91.00-93.63 | 6.75E-05 | 0.10 |
| TDWS | 2 4 B | AX_111615790 | 1B | C/A | 222.29-222.29 | 2.08E-04 | 0.08 |
| TDWS | 4 B | BobWhite_c16206_317 | 1B | G/A | 603.22-603.22 | 5.21E-04 | 0.07 |
| TDWS | 2 3 4 B | RFL_Contig2826_548 | 1B | T/C | 622.57-622.57 | 3.57E-04 | 0.08 |
| TDWS | 2 4 B | TA001769-0538 | 1B | T/C | 633.33-633.33 | 9.43E-05 | 0.09 |
| TDWS | 3 4 B | AX_110941446 | 1B | T/C | 648.03-648.03 | 7.67E-04 | 0.07 |
| TDWS | 2 3 4 B | Ra_c42714_1137 | 2A | A/G | 605.80-605.80 | 2.06E-05 | 0.11 |
| TDWS | 2 4 | RFL_Contig3780_644 | 2A | G/A | 676.24-676.25 | 7.93E-04 | 0.07 |
| TDWS | 4 B | AX_109841008 | 2A | C/T | 746.75-746.75 | 2.06E-04 | 0.08 |
| TDWS | 3 4 | AX_108942936 | 2B | T/C | 30.27-30.27 | 1.01E-04 | 0.09 |
| TDWS | 2 3 4 B | RAC875_c45600_228 | 2B | G/A | 339.17-339.17 | 2.55E-04 | 0.08 |
| TDWS | 2 3 4 B | RAC875_c27297_2153 | 2B | G/A | 385.19-385.19 | 1.36E-04 | 0.09 |
| TDWS | 2 3 | AX_111235991 | 2B | A/C | 626.54-626.54 | 7.63E-04 | 0.06 |
| TDWS | 2 4 B | wsnp_BF473744B_Ta_2_2 | 2B | C/A | 647.11-648.08 | 3.83E-04 | 0.08 |
| TDWS | 2 4 B | Ra_c19225_591 | 2B | A/G | 748.97-748.97 | 1.75E-04 | 0.09 |
| TDWS | 2 4 B | AX_110121961 | 2D | C/T | 36.08-36.08 | 2.15E-04 | 0.08 |
| TDWS | 4 B | AX_111680512 | 2D | G/A | 566.16-566.16 | 5.75E-04 | 0.07 |
| TDWS | 2 3 4 B | AX_111079825 | 3A | G/A | 7.28-10.22 | 7.36E-05 | 0.10 |
| TDWS | 3 4 B | AX_111586409 | 3A | G/A | 647.36-654.32 | 7.78E-05 | 0.10 |
| TDWS | 3 4 B | AX_108833035 | 3A | C/A | 721.66-731.46 | 1.30E-04 | 0.09 |
| TDWS | 2 3 4 B | AX_94602255 | 3B | C/T | 6.38-6.38 | 2.70E-04 | 0.08 |
| TDWS | 2 3 4 B | AX_111511076 | 3B | G/A | 26.76-26.76 | 1.42E-04 | 0.09 |
| TDWS | 2 B | Excalibur_c35645_587 | 3B | A/C | 40.65-43.28 | 4.39E-04 | 0.08 |
| TDWS | 2 B | AX_109407099 | 3B | C/T | 140.13-140.13 | 9.11E-04 | 0.07 |
| TDWS | 4 B | BS00091643_51 | 3B | A/G | 603.45-603.45 | 3.55E-04 | 0.08 |
| TDWS | 2 4 B | AX_111231933 | 3B | C/A | 740.93-744.35 | 4.27E-04 | 0.08 |
| TDWS | 3 B | AX_109816383 | 3B | C/G | 810.31-810.31 | 9.14E-04 | 0.07 |
| TDWS | 4 B | AX_89576720 | 3D | C/T | 481.57-481.57 | 9.43E-04 | 0.07 |
| TDWS | 3 4 B | AX_94693080 | 3D | A/G | 511.32-511.32 | 5.50E-04 | 0.07 |
| TDWS | 4 B | AX_110541173 | 4A | A/G | 572.56-572.56 | 4.93E-04 | 0.07 |
| TDWS | 2 3 B | AX_111640784 | 4B | C/T | 12.09-12.51 | 1.98E-04 | 0.09 |
| TDWS | 2 4 B | AX_89323611 | 4B | A/G | 32.25-37.58 | 5.96E-06 | 0.13 |
| TDWS | 2 B | AX_94957045 | 4B | C/T | 42.05-46.62 | 5.33E-04 | 0.07 |
| TDWS | 4 B | AX_109283891 | 4B | A/C | 643.92-643.92 | 5.64E-04 | 0.07 |
| TDWS | 3 B | AX_94490737 | 4B | T/C | 652.27-653.32 | 3.09E-04 | 0.08 |
| TDWS | 2 3 B | AX_111979632 | 4D | C/T | 15.42-24.34 | 2.32E-04 | 0.08 |
| TDWS | 2 4 B | AX_109987236 | 4D | G/A | 47.32-47.32 | 9.63E-04 | 0.07 |
| TDWS | 3 4 | wsnp_Ex_rep_c67292_65834396 | 5A | G/A | 456.61-467.38 | 3.76E-04 | 0.07 |
| TDWS | 2 4 B | AX_109817762 | 5A | T/G | 471.69-476.67 | 8.83E-05 | 0.10 |
| TDWS | 2 4 B | RAC875_c46811_128 | 5A | G/A | 663.28-663.28 | 3.87E-04 | 0.08 |
| TDWS | 4 B | GENE-2826_325 | 5A | A/C | 688.32-688.32 | 3.71E-04 | 0.08 |
| TDWS | 3 B | AX_94456702 | 5A | C/T | 706.93-706.93 | 4.74E-04 | 0.08 |
| TDWS | 4 B | AX_111109252 | 5B | A/G | 387.31-394.74 | 1.31E-04 | 0.09 |
| TDWS | 4 B | AX_109415761 | 5B | A/G | 399.77-399.77 | 6.34E-04 | 0.07 |
| TDWS | 4 B | AX_108995332 | 5B | T/A | 427.25-427.82 | 4.81E-04 | 0.07 |
| TDWS | 2 4 B | AX_111486576 | 5B | G/T | 439.06-447.45 | 1.91E-06 | 0.14 |
| TDWS | 1 2 4 B | AX_108820026 | 5B | T/C | 470.41-477.73 | 4.06E-07 | 0.16 |
| TDWS | 4 B | AX_110522400 | 5B | C/T | 491.5-492.79 | 3.29E-06 | 0.14 |
| TDWS | 2 3 4 B | AX_110464564 | 5D | C/T | 49.18-49.18 | 8.69E-07 | 0.15 |
| TDWS | 4 B | AX_94505455 | 5D | C/T | 335.24-335.24 | 6.14E-04 | 0.07 |
| TDWS | 2 4 B | AX_94976637 | 5D | A/G | 371.27-371.27 | 3.65E-05 | 0.11 |
| TDWS | 2 4 B | AX_94524902 | 5D | A/C | 547.50-547.50 | 1.65E-04 | 0.09 |
| TDWS | 2 4 B | RAC875_c63933_184 | 5D | A/G | 556.43-556.72 | 2.24E-04 | 0.08 |
| TDWS | 2 4 B | AX_111477118 | 6A | C/A | 5.21-12.96 | 6.82E-05 | 0.10 |
| TDWS | 4 B | AX_110052843 | 6B | G/T | 113.77-115.95 | 7.46E-04 | 0.07 |
| TDWS | 4 B | AX_108941919 | 6B | A/G | 487.55-487.55 | 5.88E-04 | 0.07 |
| TDWS | 2 3 4 B | GENE-3980_654 | 6B | T/C | 640.9-643.79 | 1.50E-04 | 0.09 |
| TDWS | 2 3 4 B | Tdurum_contig41142_267 | 6B | G/T | 664.46-664.46 | 1.22E-04 | 0.09 |
| TDWS | 2 B | AX_110130742 | 6B | G/A | 704.86-708.22 | 2.48E-04 | 0.08 |
| TDWS | 2 B | AX_94965830 | 6D | C/T | 5.57-5.82 | 2.54E-05 | 0.11 |
| TDWS | 2 4 B | Excalibur_c4615_1610 | 6D | A/G | 391.92-391.92 | 1.10E-04 | 0.09 |
| TDWS | 3 4 B | wsnp_CAP11_c1761_958064 | 7A | A/G | 589.24-589.24 | 8.36E-04 | 0.07 |
| TDWS | 4 B | BobWhite_c17095_237 | 7A | G/A | 611.63-613.73 | 4.11E-06 | 0.13 |
| TDWS | 1 4 | AX_110044150 | 7A | A/G | 621.86-623.45 | 1.69E-04 | 0.09 |
| TDWS | 3 4 | AX_111568850 | 7D | A/G | 75.92-86.98 | 5.30E-04 | 0.07 |
| TDWS | 2 4 B | AX_111132781 | 7D | C/A | 129.97-135.37 | 2.38E-04 | 0.08 |
| TDWS | 3 4 | AX_111577151 | 7D | T/C | 480.4-480.4 | 4.41E-04 | 0.07 |
| SN | 2 4 B | CAP7_c3299_316 | 1A | A/G | 11.67-11.67 | 1.90E-04 | 0.08 |
| SN | 2 B | BobWhite_c46007_582 | 1A | A/G | 512.66-512.66 | 6.83E-04 | 0.07 |
| SN | 2 B | BS00023084_51 | 1B | A/G | 7.48-7.48 | 9.29E-04 | 0.07 |
| SN | 4 B | BS00067567_51 | 1B | A/G | 40.88-40.88 | 9.71E-04 | 0.07 |
| SN | 2 B | CAP12_c2858_94 | 1B | A/G | 572.29-572.29 | 9.42E-04 | 0.07 |
| SN | 2 B | Ra_c7005_420 | 1B | A/G | 588.63-588.77 | 1.15E-04 | 0.09 |
| SN | 2 B | AX_111547803 | 1D | C/T | 29.49-29.49 | 2.44E-05 | 0.11 |
| SN | 2 B | AX_110993075 | 2A | C/T | 766.47-773.63 | 1.10E-04 | 0.09 |
| SN | 3 B | CAP11_c527_306 | 3A | C/A | 10.22-13.09 | 2.29E-04 | 0.08 |
| SN | 4 B | AX_108833035 | 3A | C/A | 731.43-733.96 | 6.49E-04 | 0.07 |
| SN | 2 B | AX_109864825 | 3B | G/C | 581.08-581.08 | 6.86E-04 | 0.07 |
| SN | 4 B | GENE_1785_626 | 3B | A/G | 760.14-770.68 | 2.22E-04 | 0.08 |
| SN | 2 B | AX_94855510 | 3D | G/A | 22.77-22.77 | 9.81E-04 | 0.07 |
| SN | 2 B | AX_110567511 | 3D | A/G | 551.02-551.02 | 4.37E-04 | 0.07 |
| SN | 4 B | D_GA8KES402JVT1Y_74 | 3D | G/A | 571.55-571.55 | 2.79E-04 | 0.08 |
| SN | 2 4 B | AX_110960365 | 4A | G/C | 619.38-621.43 | 6.88E-04 | 0.07 |
| SN | 2 B | AX_110951465 | 4B | G/A | 12.09-13.84 | 1.93E-04 | 0.08 |
| SN | 2 4 | AX_111056917 | 4B | G/A | 569.33-569.33 | 4.70E-04 | 0.08 |
| SN | 2 B | AX_109924771 | 5A | G/T | 368.18-368.18 | 3.10E-04 | 0.08 |
| SN | 3 B | AX_110965703 | 5A | T/C | 706.22-706.22 | 9.96E-04 | 0.06 |
| SN | 2 B | Ex_c5594_2818 | 5B | A/G | 520.89-520.89 | 3.38E-04 | 0.08 |
| SN | 2 B | wsnp_Ku_c3151_5892200 | 5B | C/A | 680.85-680.85 | 5.87E-04 | 0.07 |
| SN | 2 4 B | IAAV1981 | 5B | A/G | 692.63-701.52 | 6.01E-04 | 0.07 |
| SN | 2 4 B | AX_110464564 | 5D | C/T | 47.28-49.18 | 2.95E-04 | 0.08 |
| SN | 2 B | AX_111670328 | 6A | A/C | 550.08-550.08 | 6.28E-04 | 0.07 |
| SN | 2 B | AX_109933853 | 6B | T/C | 447.47-447.47 | 2.50E-04 | 0.08 |
| SN | 2 B | wsnp_Ex_c24766_34017588 | 6B | T/C | 569.03-574.66 | 3.18E-04 | 0.08 |
| SN | 2 B | Tdurum_contig46925_285 | 6B | G/A | 582.34-582.34 | 3.67E-04 | 0.08 |
| SN | 2 B | Tdurum_contig47269_904 | 6B | G/A | 593.26-595.29 | 2.78E-04 | 0.08 |
| SN | 2 B | wsnp_Ex_c18744_27620403 | 6B | G/A | 603.36-606.43 | 1.75E-04 | 0.09 |
| SN | 2 B | BobWhite_c13435_700 | 6D | T/C | 471.01-471.01 | 6.25E-04 | 0.07 |
| SN | 2 B | AX_111468930 | 7A | A/C | 73.81-73.81 | 4.36E-04 | 0.07 |
| SN | 2 B | AX_110598508 | 7B | G/C | 744.60-744.60 | 7.01E-04 | 0.07 |
| SN | 2 4 | AX_110773195 | 7D | A/G | 129.28-129.28 | 4.80E-04 | 0.08 |
| SN | 2 B | AX_110425263 | 7D | T/A | 592.86-592.86 | 4.72E-05 | 0.10 |
| KN | 2 B | AX_108873053 | 1A | C/T | 29.05-29.05 | 7.41E-04 | 0.07 |
| KN | 2 B | AX_109483727 | 1A | G/A | 39.64-39.64 | 6.11E-04 | 0.08 |
| KN | 4 B | Kukri_c16271_894 | 1A | C/A | 566.48-572.35 | 5.50E-04 | 0.08 |
| KN | 2 4 | Ra_c69176_1308 | 1B | A/C | 107.14-107.14 | 5.53E-04 | 0.07 |
| KN | 2 4 | RAC875_c51459_311 | 2A | A/G | 779.67-779.67 | 5.06E-04 | 0.07 |
| KN | 2 B | AX_109874896 | 2B | T/C | 733.19-733.2 | 4.04E-04 | 0.08 |
| KN | 2 4 | RAC875_c35200_230 | 2D | G/A | 634.47-634.47 | 1.55E-04 | 0.09 |
| KN | 2 B | AX_110940627 | 3A | A/G | 719.27-719.60 | 2.84E-04 | 0.09 |
| KN | 4 B | AX_108833035 | 3A | C/A | 731.43-731.43 | 9.52E-04 | 0.07 |
| KN | 2 B | AX_111355929 | 3D | C/A | 514.28-514.28 | 2.77E-04 | 0.09 |
| KN | 4 B | Excalibur_c14401_404 | 4B | G/A | 6.15-6.15 | 8.55E-05 | 0.10 |
| KN | 2 4 | AX_110554000 | 4D | G/A | 499.10-499.10 | 2.17E-05 | 0.11 |
| KN | 1 B | AX_110529756 | 5A | T/C | 29.38-29.38 | 5.59E-04 | 0.08 |
| KN | 2 B | AX_95085353 | 5A | C/T | 564.60-564.60 | 7.21E-04 | 0.07 |
| KN | 2 4 | BS00041063_51 | 5A | G/A | 594.35-594.96 | 3.88E-06 | 0.13 |
| KN | 2 B | AX_109590833 | 5B | G/C | 545.27-545.27 | 7.66E-04 | 0.07 |
| KN | 4 B | GENE-3980_654 | 6B | T/C | 640.98-640.98 | 8.13E-04 | 0.07 |
| KN | 2 4 B | Tdurum_contig41142_267 | 6B | G/T | 664.38-664.46 | 1.37E-04 | 0.09 |
| KN | 2 B | Tdurum_contig10729_734 | 6B | T/C | 720.98-720.98 | 8.03E-04 | 0.07 |
| KN | 3 B | AX_95089256 | 6D | T/G | 462.63-462.63 | 9.72E-04 | 0.07 |
| KN | 4 B | BS00022757_51 | 7A | G/A | 515.20-515.20 | 9.17E-06 | 0.13 |
| KN | 4 B | AX_94617779 | 7B | A/G | 133.75-133.75 | 9.98E-04 | 0.07 |
| KN | 4 B | AX_108814420 | 7B | T/C | 156.43-156.43 | 8.97E-04 | 0.07 |
| KN | 4 B | AX_111502711 | 7B | T/C | 191.21-191.21 | 2.36E-04 | 0.09 |
| KN | 4 B | AX_111517872 | 7B | A/T | 337.53-337.53 | 1.83E-04 | 0.09 |
| TGW | 3 4 B | AX_108753536 | 1A | C/G | 429.71-429.71 | 4.72E-04 | 0.06 |
| TGW | 4 B | AX_109002944 | 1A | G/A | 517.38-517.38 | 7.23E-04 | 0.05 |
| TGW | 2 B | AX_108939577 | 1A | G/A | 554.24-556.80 | 1.48E-04 | 0.07 |
| TGW | 3 B | AX_110587308 | 1A | G/A | 587.84-587.84 | 9.37E-04 | 0.05 |
| TGW | 1 3 | AX_110675625 | 1B | C/T | 510.06-510.06 | 3.50E-04 | 0.07 |
| TGW | 2 B | AX_109410086 | 1B | G/C | 581.2-581.21 | 5.80E-04 | 0.05 |
| TGW | 4 B | AX_111496323 | 1D | C/G | 11.40-11.40 | 7.60E-04 | 0.05 |
| TGW | 3 B | AX_111005064 | 1D | A/G | 430.04-430.04 | 9.45E-04 | 0.05 |
| TGW | 2 B | AX_108899874 | 2A | G/A | 2.66-2.82 | 3.00E-04 | 0.06 |
| TGW | 3 B | AX_111037158 | 2A | T/C | 27.33-27.33 | 5.33E-04 | 0.05 |
| TGW | 2 B | BS00065993_51 | 2B | G/A | 133.7-140.85 | 5.50E-04 | 0.05 |
| TGW | 1 2 B | AX_111102999 | 2B | A/G | 418.62-427.55 | 3.58E-04 | 0.06 |
| TGW | 2 3 B | AX_109862024 | 2B | A/G | 748.98-748.98 | 1.44E-04 | 0.07 |
| TGW | 3 4 B | AX_110021697 | 2B | C/T | 773.46-773.56 | 3.81E-04 | 0.06 |
| TGW | 2 4 B | AX_110938283 | 2D | C/T | 62.61-68.80 | 1.77E-04 | 0.06 |
| TGW | 2 3 4 B | AX_110926015 | 3A | A/G | 140.80-140.80 | 1.53E-04 | 0.07 |
| TGW | 1 2 B | AX_109313803 | 3A | G/A | 715.81-725.74 | 5.17E-04 | 0.06 |
| TGW | 3 B | CAP8_c8016_120 | 3B | A/G | 139.78-139.78 | 8.78E-04 | 0.05 |
| TGW | 2 B | AX_94855940 | 3B | C/T | 338.61-338.61 | 7.94E-04 | 0.05 |
| TGW | 2 3 B | AX_109983327 | 3B | T/A | 704.74-704.74 | 1.37E-04 | 0.07 |
| TGW | 2 3 B | GENE_1785_118 | 3B | A/G | 760.14-760.14 | 3.92E-04 | 0.06 |
| TGW | 1 2 3 B | D_GA8KES402JVT1Y_74 | 3D | G/A | 571.55-571.55 | 1.99E-04 | 0.06 |
| TGW | 4 B | AX_109994105 | 3D | G/C | 588.85-588.85 | 9.29E-04 | 0.05 |
| TGW | 1 3 | RAC875_c14911_1282 | 4A | A/G | 68.49-68.56 | 1.80E-04 | 0.07 |
| TGW | 2 3 B | Kukri_c34633_69 | 4B | A/G | 21.38-27.24 | 1.32E-04 | 0.07 |
| TGW | 1 2 4 B | AX_111548406 | 4B | T/A | 40.44-44.95 | 1.67E-05 | 0.09 |
| TGW | 2 B | AX_108770543 | 4B | C/T | 50.73-57.48 | 1.14E-04 | 0.07 |
| TGW | 2 3 B | AX_110471449 | 4B | A/C | 103.13-114.95 | 2.57E-04 | 0.06 |
| TGW | 1 2 B | TA002919-0864 | 4B | C/T | 530.21-530.21 | 8.08E-04 | 0.05 |
| TGW | 2 3 B | IAAV3758 | 4D | A/G | 11.57-11.57 | 2.69E-04 | 0.06 |
| TGW | 4 B | wsnp_Ex_c7383_12655992 | 5A | G/A | 481.9-482.12 | 3.22E-04 | 0.06 |
| TGW | 2 4 B | AX_89340428 | 5A | G/A | 684.94-684.94 | 2.70E-04 | 0.06 |
| TGW | 1 2 B | AX_110958315 | 5A | G/A | 706.22-708.02 | 1.78E-04 | 0.06 |
| TGW | 2 4 B | AX_110528649 | 5B | T/G | 21.17-28.45 | 6.87E-05 | 0.07 |
| TGW | 1 3 4 B | AX_95202139 | 5B | C/T | 378.85-378.85 | 3.86E-04 | 0.06 |
| TGW | 1 B | GENE-3574_643 | 5B | T/C | 519.15-527.26 | 6.11E-04 | 0.05 |
| TGW | 2 3 B | AX_108769612 | 5B | C/G | 696.29-696.40 | 1.69E-04 | 0.07 |
| TGW | 2 B | AX_111607084 | 6A | G/T | 48.88-48.88 | 9.13E-04 | 0.05 |
| TGW | 1 B | AX_110553720 | 6B | T/C | 157.11-157.11 | 5.82E-04 | 0.05 |
| TGW | 4 B | AX_109334927 | 6B | A/C | 190.18-197.06 | 3.66E-04 | 0.06 |
| TGW | 4 B | AX_108953754 | 6B | C/T | 213.36-213.83 | 6.08E-04 | 0.05 |
| TGW | 1 4 B | AX_110084655 | 6B | T/A | 226.39-226.39 | 6.19E-04 | 0.05 |
| TGW | 4 B | AX_94761192 | 6B | A/G | 452.05-453.93 | 2.00E-04 | 0.06 |
| TGW | 4 B | AX_109923836 | 6B | T/C | 488.55-498.91 | 4.87E-04 | 0.06 |
| TGW | 1 2 3 B | AX_108929087 | 6B | G/C | 673.95-677.48 | 3.52E-06 | 0.10 |
| TGW | 1 2 B | Excalibur_c4615_1610 | 6D | A/G | 391.92-391.92 | 1.04E-04 | 0.07 |
| TGW | 1 3 4 B | AX_109419278 | 7A | T/C | 88.83-88.83 | 1.32E-04 | 0.07 |
| TGW | 1 3 4 B | AX_109359873 | 7A | G/A | 513.37-513.37 | 2.41E-04 | 0.06 |
| TGW | 1 3 | BobWhite_c5970_731 | 7B | A/G | 9.70-9.70 | 8.84E-04 | 0.06 |
| TGW | 1 2 B | Tdurum_contig11028_236 | 7B | G/A | 51.07-52.57 | 4.83E-04 | 0.06 |
| TGW | 1 B | AX_109851527 | 7B | G/T | 743.53-743.53 | 6.71E-04 | 0.05 |
| TGW | 4 B | AX_89425861 | 7D | A/G | 63.88-63.88 | 5.34E-04 | 0.05 |
| TGW | 3 B | AX_109577030 | 7D | G/T | 89.02-89.54 | 5.53E-04 | 0.05 |
| TGW | 2 B | AX_109421587 | 7D | G/A | 572.62-572.62 | 9.32E-04 | 0.05 |
| TGW | 1 B | AX_94436382 | 7D | T/C | 632.01-632.01 | 6.73E-04 | 0.05 |
| GY | 2 B | AX_109582739 | 1A | C/G | 109.32-109.32 | 9.38E-04 | 0.05 |
| GY | 2 B | AX_110172892 | 1A | C/T | 159.86-162.53 | 6.54E-04 | 0.05 |
| GY | 2 B | AX_109457230 | 1A | G/A | 177.46-177.46 | 1.99E-04 | 0.06 |
| GY | 2 B | AX_109101297 | 1A | G/A | 242.01-242.01 | 7.98E-04 | 0.05 |
| GY | 1 4 B | AX_109830195 | 1A | T/C | 399.48-412.22 | 2.56E-05 | 0.08 |
| GY | 1 4 B | AX_110953451 | 1A | G/C | 419.44-424.80 | 1.42E-05 | 0.09 |
| GY | 1 4 B | AX_110507437 | 1A | C/T | 429.71-440.41 | 3.23E-05 | 0.08 |
| GY | 3 4 B | AX_109602295 | 1A | A/G | 576.23-577.46 | 4.07E-04 | 0.06 |
| GY | 3 B | AX_111673781 | 1B | G/A | 34.42-41.53 | 2.97E-04 | 0.06 |
| GY | 4 B | AX_95133499 | 1B | G/C | 452.32-452.32 | 8.98E-04 | 0.05 |
| GY | 2 B | AX_110424417 | 1B | C/T | 478.04-478.04 | 2.38E-04 | 0.06 |
| GY | 2 B | AX_110986480 | 2A | T/C | 27.33-27.33 | 7.51E-04 | 0.05 |
| GY | 1 2 B | AX_110410927 | 2A | G/A | 60.61-60.61 | 7.20E-04 | 0.05 |
| GY | 4 B | AX_111681853 | 2A | G/A | 635.60-635.60 | 5.11E-04 | 0.05 |
| GY | 4 B | AX_109839131 | 2A | G/C | 693.30-694.59 | 2.58E-04 | 0.06 |
| GY | 4 B | AX_111197359 | 2A | C/T | 733.56-733.56 | 4.94E-04 | 0.05 |
| GY | 3 4 B | AX_94808625 | 2B | T/C | 24.91-31.63 | 2.12E-04 | 0.06 |
| GY | 1 2 | AX_109856434 | 2B | A/G | 106.00-108.68 | 8.10E-05 | 0.08 |
| GY | 1 B | AX_109851949 | 2B | T/C | 742.08-742.08 | 3.44E-04 | 0.06 |
| GY | 4 B | AX_110480872 | 2B | C/T | 776.45-776.45 | 7.82E-04 | 0.05 |
| GY | 1 3 B | AX_111585698 | 3A | C/T | 720.46-722.57 | 1.76E-05 | 0.09 |
| GY | 3 4 | AX_108981823 | 3B | C/G | 229.16-229.16 | 7.63E-04 | 0.06 |
| GY | 2 B | AX_109095224 | 3B | G/T | 279.14-279.14 | 7.97E-04 | 0.05 |
| GY | 2 B | AX_110645455 | 3B | G/A | 349.30-349.30 | 2.92E-04 | 0.06 |
| GY | 3 4 B | AX_109948496 | 3D | C/T | 576.26-576.26 | 4.42E-04 | 0.06 |
| GY | 3 B | AX_94867721 | 4A | G/C | 666.54-667.19 | 1.74E-04 | 0.06 |
| GY | 1 4 | AX_110641949 | 4B | C/T | 13.98-13.98 | 9.36E-04 | 0.06 |
| GY | 2 B | AX_111734448 | 4B | T/C | 642.92-642.92 | 2.79E-04 | 0.06 |
| GY | 4 B | AX_108765521 | 4B | A/G | 664.19-664.64 | 3.07E-04 | 0.06 |
| GY | 1 3 | AX_109076951 | 5A | C/A | 647.35-647.35 | 4.44E-04 | 0.07 |
| GY | 3 B | AX_111656541 | 5A | G/A | 698.46-698.46 | 7.35E-04 | 0.05 |
| GY | 1 B | wsnp_Ex_c2171_4074003 | 5A | G/A | 706.22-708.44 | 6.62E-04 | 0.05 |
| GY | 4 B | AX_110476637 | 5B | G/A | 48.57-48.57 | 8.23E-04 | 0.05 |
| GY | 3 4 B | AX_108777632 | 5B | A/G | 603.96-605.22 | 2.11E-05 | 0.08 |
| GY | 4 B | AX_94486057 | 5B | G/A | 622.63-622.63 | 5.48E-04 | 0.05 |
| GY | 2 B | AX_111192525 | 5B | G/T | 687.46-692.68 | 3.86E-04 | 0.06 |
| GY | 4 B | AX_94501458 | 5D | G/C | 489.89-489.89 | 1.53E-04 | 0.07 |
| GY | 3 B | wsnp_Ku_c2119_4098330 | 6B | A/C | 8.41-8.41 | 9.39E-04 | 0.05 |
| GY | 4 B | Kukri_c31995_1948 | 6D | A/G | 402.13-403.65 | 5.21E-04 | 0.05 |
| GY | 4 B | AX_109371456 | 7A | C/G | 33.50-33.54 | 5.12E-04 | 0.05 |
| GY | 3 B | AX_109309222 | 7B | C/T | 491.11-496.27 | 2.79E-04 | 0.06 |
| GY | 3 B | AX_109403607 | 7B | G/A | 504.89-510.63 | 5.90E-04 | 0.05 |
| GY | 1 3 4 B | AX_95235606 | 7B | A/G | 630.66-630.66 | 1.38E-04 | 0.07 |
| GY | 2 B | AX_111918348 | 7D | T/C | 77.23-80.12 | 2.51E-04 | 0.06 |
| GY | 2 B | AX_109577030 | 7D | G/T | 89.02-89.02 | 8.19E-04 | 0.05 |

Note: LWC, leaf water content; SPWC, spike water content; STWC, stem water content; LDWS, leaf dry weights; SPDWS, spike dry weights; STDWS, stem dry weights; TDWS, total dry weights; Pn, light-saturated net photosynthetic rate; Gs, stomatal conductance; Tr, transpiration rate; Ci, intercellular CO_2_ concentration; WUE, water use efficiency; iWUE, intrinsic water use efficiency; Fv′/Fm′, the maximum quantum yield of PSII photochemistry; PH, plant height; LAI, leaf area index; GFR, grain filling rate; SGT, stay green trait; TTF, thermal time from sowing to flowering stage; TTM, thermal time from sowing to maturity stage; SS, spike shape; FLL, flag leaf length; FLW, flag leaf width; FLA, flag leaf area; FLB, flag leaf biomass; FSLA, flag leaf specific leaf area; FLANG, flag leaf angle; SPAD, Chlorophyll content SPAD meter reading; SN, spikes number per square meter; KN, kernels number per spike; TGW, thousand-grain weight; GY, grain yield. The same below.

**Table S3**. Distribution of pleiotropic loci associated with three or more grain yield related traits on wheat chromosomes.

| Chr | Trait | Interval (Mb) | Representative marker |
| --- | --- | --- | --- |
| 1A | GY/STDWS/PH | 242.01-246.69 | AX_109101297 |
| 1A | GY/Ci/PH | 396.24-404.42 | AX_109905627 |
| 1A | Ci/GY/TGW/PH | 424.27-432.95 | AX_109097017 |
| 1A | FSLA/FLL/PH/Pn/TGW | 516.07-5177.52 | AX_109002944 |
| 1A | TGW/PH/KN/SPDWS | 554.24-567.45 | AX_108939577 |
| 1A | LWC/GFR/GY/SPAD | 574.34-579.15 | AX_109602295 |
| 1A | TGW/SS/FLANG | 587.84-593.31 | AX_110587308 |
| 1B | PH/GY/SS/FLW/Gs/iWUE/SN/SPAD/LWC | 38.83-43.18 | AX_108954078 |
| 1B | TGW/FLANG/LDWS/STDWS | 581.20-582.91 | AX_109410086 |
| 1D | TTF/Gs/FLL/FLA/TGW | 7.04-11.52 | AX_111496323 |
| 1D | TGW/FLL/FLA | 430.04-432.71 | AX_111005064 |
| 2A | TGW/SGT/TTF/FLA/FLB | 2.66-7.80 | AX_108899874 |
| 2A | TGW/TTF/GY/FLL/PH/Pn | 27.33-32.87 | AX_110986480 |
| 2A | FLL/GY/FLB | 731.25-734.35 | AX_111197359 |
| 2B | FLL/TTF/FLB/GFR/LDWS/STDWS/TDWS/GY | 29.14-31.63 | AX_110624590 |
| 2B | TTF/GY/SS/SPWC | 105.99-108.68 | AX_110974464 |
| 2B | GY/FLA/FLL/PH/FLANG/FLA/STDWS/TDWS/TGW | 742.08-748.98 | AX_109851949 |
| 2B | iWUE/TGW/FLB/GY | 773.35-776.45 | AX_110021697 |
| 3A | SS/Pn/TGW | 137.10-140.80 | AX_110926015 |
| 3A | FLB/TGW/STDWS/PH/GFR/KN/GY | 714.30-720.71 | AX_110982768 |
| 3B | FSLA/TGW/TDWS/FLB | 138.37-141.13 | CAP8_c8016_120 |
| 3B | SPAD/GY/PH/FLANG | 278.88-281.83 | AX_109095224 |
| 3B | SPAD/GY/FLANG | 348.49-349.98 | AX_110645455 |
| 3B | TTM/TTF/TGW/SN/FLANG | 759.18-764.75 | GENE_1785_626 |
| 3D | TTF/Pn/SN/TGW/GFR | 570.57-576.26 | AX_109994105 |
| 4A | SPWC/PH/FLL/GY | 659.13-667.19 | AX_94867721 |
| 4B | TGW/LDWS/GFR | 26.83-31.97 | AX_89498126 |
| 4B | TGW/STDWS/TDWS/GFR/LDWS | 44.95-46.62 | AX_110527790 |
| 4B | GY/SGT/STDWS/GFR/TDWS | 642.92-644.83 | AX_111734448 |
| 4B | SS/SPWC/GY | 657.25-664.64 | AX_108765521 |
| 4D | STDWS/FLB/GFR/TDWS/TGW | 9.31-11.57 | IAAV3758 |
| 5A | TGW/SS/TTF/SPWC/GFR | 684.94-685.79 | AX_89340428 |
| 5A | SS/FLL/GY | 698.21-699.48 | AX_111656541 |
| 5A | PH/FLANG/GY/TGW/STDWS/FLB/LDWS/STDWS/TDWS | 703.20-708.77 | AX_110514148 |
| 5B | PH/TGW/FLA | 19.70-21.40 | wsnp_Ex_c58091_59534826 |
| 5B | TTF/TGW/SN | 519.15-520.89 | GENE-3574_643 |
| 5B | LWC/TGW/FLW/FSLA/PH | 527.26-533.51 | RAC875_c18335_443 |
| 5B | GFR/SPDWS/SN/FLL/TTM/GY | 679.33-687.46 | AX_109476837 |
| 6B | FLA/GY/PH | 6.04-8.89 | wsnp_Ku_c2119_4098330 |
| 6B | TGW/LDWS/FLB | 226.39-233.03 | AX_109861222 |
| 6B | FSLA/STWC/TGW/SPWC | 449.23-454.04 | AX_94761192 |
| 6D | FLANG/PH/GFR/LDWS/SPDWS/STDWS/TDWS/TGW/FLL | 388.23-392.03 | Excalibur_c4615_1610 |
| 7A | TTM/GY/FSLA/FLW/SPWC/TTF | 33.06-35.40 | AX_109371456 |
| 7A | TGW/FLB/KN | 512.76-515.20 | AX_109818549 |
| 7B | TTF/PH/GY | 629.43-632.34 | AX_95235606 |
| 7D | SPDWS/TDWS/GFR/GY | 86.98-89.54 | AX_111046029 |
| 7D | GFR/TGW/FSLA | 566.55-576.75 | AX_109421587 |

Note: LWC, leaf water content; SPWC, spike water content; STWC, stem water content; LDWS, leaf dry weights; SPDWS, spike dry weights; STDWS, stem dry weights; TDWS, total dry weights; Pn, light-saturated net photosynthetic rate; Gs, stomatal conductance; Tr, transpiration rate; Ci, intercellular CO_2_ concentration; WUE, water use efficiency; iWUE, intrinsic water use efficiency; Fv’/Fm’, the maximum quantum yield of PSII photochemistry; PH, plant height; LAI, leaf area index; GFR, grain filling rate; SGT, stay green trait; TTF, thermal time from sowing to flowering stage; TTM, thermal time from sowing to maturity stage; SS, spike shape; FLL, flag leaf length; FLW, flag leaf width; FLA, flag leaf area; FLB, flag leaf biomass; FSLA, flag leaf specific leaf area; FLANG, flag leaf angle; SPAD, Chlorophyll content SPAD meter reading; SN, spikes number per square meter; KN, kernels number per spike; TGW, thousand-grain weight; GY, grain yield. The same below.

**Table S4**. 32 wheat traits values within each cluster (1, 2, 3, 4 and 5).

|  | Cluster1 | Cluster2 | Cluster3 | Cluster4 | Cluster5 |
| --- | --- | --- | --- | --- | --- |
| Pn | 23.71±1.51 | 22.15±1.48 | 19.71±2.10 | 20.05±1.39 | 21.01±1.64 |
| Gs | 0.44±0.05 | 0.44±0.06 | 0.35±0.05 | 0.32±0.06 | 0.38±0.06 |
| Tr | 4.65±0.46 | 4.50±0.50 | 3.83±0.42 | 3.57±0.52 | 3.80±0.50 |
| Ci | 277.48±10.51 | 284.71±13.47 | 273.52±12.46 | 266.52±12.84 | 274.39±12.40 |
| WUE | 5.21±0.50 | 4.96±0.59 | 5.14±0.75 | 5.85±0.49 | 5.64±0.59 |
| iWUE | 54.28±4.07 | 51.55±7.11 | 58.47±6.38 | 63.54±6.88 | 59.08±6.63 |
| Fvʹ/Fmʹ | 0.61±0.02 | 0.61±0.03 | 0.61±0.02 | 0.59±0.04 | 0.61±0.02 |
| PH | 89.80±6.26 | 87.24±5.77 | 97.01±14.22 | 113.03±22.46 | 92.56±5.50 |
| LAI | 4.22±0.47 | 4.54±0.33 | 4.68±0.36 | 4.38±0.54 | 4.60±0.33 |
| GFR | 0.004±0.001 | 0.003±0.001 | 0.003±0.001 | 0.003±0.001 | 0.003±0.001 |
| SGT | 0.41±0.18 | 0.38±0.12 | 0.37±0.15 | 0.48±0.02 | 0.34±0.10 |
| TTF | 1743.99±36.5 | 1741.12±34.49 | 1716.25±31.90 | 1786.85±90.47 | 1733.80±27.32 |
| TTM | 2416.08±47.20 | 2396.73±47.23 | 2353.31±67.75 | 2438.80±91.23 | 2382.84±49.96 |
| SS | 3.58±0.63 | 3.92±0.60 | 3.32±0.75 | 2.31±0.49 | 3.38±0.72 |
| LWC | 0.66±0.03 | 0.63±0.04 | 0.64±0.02 | 0.56±0.06 | 0.59±0.04 |
| SPWC | 0.58±0.03 | 0.55±0.04 | 0.55±0.03 | 0.50±0.06 | 0.52±0.04 |
| STWC | 0.67±0.03 | 0.67±0.02 | 0.67±0.02 | 0.67±0.04 | 0.65±0.04 |
| LDWS | 0.66±0.14 | 0.42±0.06 | 0.46±0.06 | 0.36±0.07 | 0.33±0.06 |
| SPDWS | 1.18±0.24 | 0.73±0.14 | 0.83±0.13 | 0.60±0.18 | 0.59±0.12 |
| STDWS | 2.15±0.58 | 1.20±0.20 | 1.43±0.30 | 1.18±0.29 | 0.99±0.21 |
| TDWS | 390±0.87 | 2.30±0.36 | 2.67±0.38 | 2.11±0.42 | 1.87±0.35 |
| FLL | 19.88±2.62 | 16.93±1.59 | 21.64±2.18 | 20.35±3.42 | 17.28±1.87 |
| FLW | 2.01±0.13 | 1.81±0.12 | 1.72±0.16 | 1.51±0.19 | 1.62±0.15 |
| FLA | 18.85±2.54 | 15.82±1.71 | 20.39±2.34 | 18.46±2.79 | 15.87±2.04 |
| FLB | 0.19±0.04 | 0.14±0.02 | 0.16±0.03 | 0.14±0.03 | 0.13±0.02 |
| FSLA | 110.29±14.01 | 125.24±11.52 | 138.35±19.49 | 146.83±17.48 | 140.82±14.70 |
| FLANG | 2.26±0.55 | 1.69±0.48 | 2.44±0.31 | 2.53±0.54 | 2.00±0.47 |
| SPAD | 56.22±2.22 | 55.88±2.07 | 54.14±1.88 | 52.44±1.76 | 53.88±2.21 |
| KN | 43.02±2.41 | 39.77±2.45 | 39.29±3.00 | 38.61±1.74 | 38.93±2.27 |
| SN | 511.34±63.18 | 640.17±53.07 | 645.33±51.52 | 719.16±72.12 | 684.97±81.55 |
| TGW | 44.65±3.40 | 43.00±3.32 | 43.04±4.40 | 34.68±5.31 | 40.21±3.86 |
| GY | 3.38±0.22 | 3.51±0.27 | 3.06±0.36 | 2.53±0.35 | 3.34±0.32 |

Note: Pn, light-saturated net photosynthetic rate; Gs, stomatal conductance; Tr, transpiration rate; Ci, intercellular CO_2_ concentration; WUE, water use efficiency; iWUE, intrinsic water use efficiency; Fv′/Fm′, the maximum quantum yield of PSII photochemistry; PH, plant height; LAI, leaf area index; GFR, grain filling rate; SGT, stay green trait; TTF, thermal time from sowing to flowering stage; TTM, thermal time from sowing to maturity stage; SS, spike shape; LWC, leaf water content; SPWC, spike water content; STWC, stem water content; LDWS, leaf dry weights; SPDWS, spike dry weights; STDWS, stem dry weights; TDWS, total dry weights; FLL, flag leaf length; FLW, flag leaf width; FLA, flag leaf area; FLB, flag leaf biomass; FSLA, flag leaf specific leaf area; FLANG, flag leaf angle; SPAD, Chlorophyll content SPAD meter reading; KN, kernels number per spike; SN, spikes number per square meter; TGW, thousand-grain weight; GY, grain yield.
